# Relations between large-scale brain connectivity and effects of regional stimulation depend on collective dynamical state

**DOI:** 10.1371/journal.pcbi.1008144

**Published:** 2020-09-04

**Authors:** Lia Papadopoulos, Christopher W. Lynn, Demian Battaglia, Danielle S. Bassett

**Affiliations:** 1 Department of Physics & Astronomy, University of Pennsylvania, Philadelphia, Pennsylvania, United States of America; 2 Université Aix-Marseille, INSERM UMR 1106, Institut de Neurosciences des Systèmes, F-13005, Marseille, France; 3 Department of Bioengineering, University of Pennsylvania, Philadelphia, Pennsylvania, United States of America; 4 Department of Electrical & Systems Engineering, University of Pennsylvania, Philadelphia, Pennsylvania, United States of America; 5 Department of Neurology, University of Pennsylvania, Philadelphia, Pennsylvania, United States of America; 6 Department of Psychiatry, University of Pennsylvania, Philadelphia, Pennsylvania, United States of America; 7 Santa Fe Institute, Santa Fe, New Mexico, United States of America; Ghent University, BELGIUM

## Abstract

At the macroscale, the brain operates as a network of interconnected neuronal populations, which display coordinated rhythmic dynamics that support interareal communication. Understanding how stimulation of different brain areas impacts such activity is important for gaining basic insights into brain function and for further developing therapeutic neurmodulation. However, the complexity of brain structure and dynamics hinders predictions regarding the downstream effects of focal stimulation. More specifically, little is known about how the collective oscillatory regime of brain network activity—in concert with network structure—affects the outcomes of perturbations. Here, we combine human connectome data and biophysical modeling to begin filling these gaps. By tuning parameters that control collective system dynamics, we identify distinct states of simulated brain activity and investigate how the distributed effects of stimulation manifest at different dynamical working points. When baseline oscillations are weak, the stimulated area exhibits enhanced power and frequency, and due to network interactions, activity in this excited frequency band propagates to nearby regions. Notably, beyond these linear effects, we further find that focal stimulation causes more distributed modifications to interareal coherence in a band containing regions’ baseline oscillation frequencies. Importantly, depending on the dynamical state of the system, these broadband effects can be better predicted by functional rather than structural connectivity, emphasizing a complex interplay between anatomical organization, dynamics, and response to perturbation. In contrast, when the network operates in a regime of strong regional oscillations, stimulation causes only slight shifts in power and frequency, and structural connectivity becomes most predictive of stimulation-induced changes in network activity patterns. In sum, this work builds upon and extends previous computational studies investigating the impacts of stimulation, and underscores the fact that both the stimulation site, and, crucially, the regime of brain network dynamics, can influence the network-wide responses to local perturbations.

## Author summary

Stimulation can be used to alter brain activity and is a therapeutic option for certain neurological conditions. However, predicting the distributed effects of local perturbations is difficult. Previous studies show that responses to stimulation depend on anatomical (or structural) coupling. In addition to structure, here we consider how stimulation effects also depend on the brain’s collective dynamical (or functional) state, arising from the coordination of rhythmic activity across large-scale networks. In a whole-brain computational model, we show that global responses to regional stimulation can indeed be contingent upon and differ across various dynamical working points. Notably, depending on the network’s oscillatory regime, stimulation can accelerate the activity of the stimulated site, and lead to widespread effects at both the new, excited frequency, as well as in a much broader frequency range including areas’ baseline frequencies. While structural connectivity is a good predictor of “excited band” changes, in some states “baseline band” effects can be better predicted by functional connectivity, which depends upon the system’s oscillatory regime. By integrating and extending past efforts, our results thus indicate that dynamical—in additional to structural—brain organization plays a role in governing how focal stimulation modulates interactions between distributed network elements.

## Introduction

The brain is a multiscale system composed of many dynamical units that interact to produce a vast array of functions. At a large scale, macroscopic regions—each containing tens of thousands of neurons—are linked by a physical web of white matter tracts that facilitate the propagation of activity between distributed network elements. At the level of large neuronal ensembles or brain areas, collective activity is often rhythmic in nature [1], and these rhythms can become temporally coordinated between distant regions, giving rise to so-called functional interactions [2]. Importantly, oscillations have been implicated in a number of cognitive processes [3–9], and coherent activity is hypothesized to play an important role in interareal communication and information transfer among distributed brain areas [5, 6, 10]. Nonetheless, despite progress in mapping and characterizing the brain’s anatomical pathways and measuring neural oscillations, a number of questions remain as to how individual components in a brain network shape and modulate system-wide dynamics.

Among these questions, understanding how large-scale, oscillatory brain dynamics respond to localized perturbations is of critical importance [7, 11–14]. Because the brain is not a closed or static system, such activity changes could be induced by sensory inputs to primary sensory areas [15, 16], different tasks [17, 18], or other internal or regulatory processes [19–22]. In addition to naturally-induced changes, stimulation techniques such as transcranial magnetic stimulation [23], direct current stimulation [24], and alternating current stimulation [25] can also be employed to invoke modulations of dynamics in a specific brain area. By combining these techniques with imaging methods like EEG and MEG [26–31], it is possible to examine how the act of exciting a particular network component modifies rhythmic neural activity. Furthermore, in addition to its utility for basic science, neurostimulation has emerged as a promising approach for treating a number of neurological and psychiatric conditions [32–34].

Yet, while prior work has often focused on characterizing the proximal effects of local perturbations, a growing body of literature indicates that regional changes to neural activity can have more widespread consequences [11–14]. The realization that stimulation can have network-wide effects necessitates further investigations into the operating principles underlying such phenomena [35–42]. Furthermore, a crucial but seemingly understudied point is that the effects of perturbing a particular brain area can depend not only on the nature or location of the perturbation, but also on the intrinsic dynamical state of the system at baseline [43–45]. In particular, recent efforts have investigated the state-dependent effects of stimulation via precise experiments [46, 47]—focusing largely on alpha-band activity in single cortical areas—and via modeling [48–50]. These studies have uncovered robust relationships between the endogenous state of rhythmic activity and the capacity of external stimulation to modulate cortical oscillations in a given brain area. However, a pivotal next step is to extend the notion of state-dependence to the case of whole-brain networks, which acknowledge the fact that regions do not operate in isolation. Rather, in the case of large-scale brain networks, the macroscopic dynamical regime of the system arises from an interplay between units’ local activity and long-range anatomical coupling [51], leading to the emergence of collective oscillatory modes [40, 52]. Although it is reasonable to hypothesize that the global state of brain network activity should play a role in determining how a focal perturbation will manifest and influence distributed functional interactions, these ideas have yet to be systematically examined.

Thus, there is now a need to concurrently investigate and merge two outstanding questions: *(1)* how regional stimulation spreads to induce distributed effects on brain network dynamics, and *(2)* how the global dynamical regime of the system impacts these effects. Here, we investigate these questions by constructing a biophysically-motivated model of large-scale, oscillatory brain activity, in which individual brain areas are modeled as Wilson-Cowan neural masses [53] coupled according to empirically-derived anatomical connectivity [51]. We first demonstrate that, in the absence of stimulation, the interareal coupling strength and the baseline excitation of the network transition the system between qualitatively distinct collective dynamical states. By providing additional excitation to a single brain area, we then systematically examine the consequences of such local stimulation on network activity. The primary contribution of this study is an exploration of how the effects of focal perturbations can depend not only on which area is stimulated, but also on the baseline dynamical regime of the non-linear model. Hence, this work builds upon previous whole-brain modeling efforts that have examined the effects of regional perturbations [35–37] with other work examining the state-dependent effects of stimulation in single cortical areas, but not large-scale networks [48, 49].

We find that in states of low baseline excitation, stimulation can significantly increase the frequency and power of regional activity, whereas in states of high background drive, local dynamics are less sensitive to perturbations. Importantly, these results show qualitative similarities and agreement with past work examining the focal effects of stimulation [48, 49]. We further find that, due to network interactions, regional perturbations can propagate and interact with brain areas’ ongoing rhythms. In particular, depending on the system working point, downstream areas that are strongly anatomically linked to the stimulated site also develop spectral components at the excited frequency of the stimulated region. Crucially, though, modifications to interareal phase-locking can additionally be induced in a broader frequency band comprising brain areas’ spontaneous, baseline oscillations, which may be well-separated from the excited frequency. Moreover, changing the dynamical regime of the system modulates the strength of associations between network-wide responses to perturbations in the baseline frequency band and structural or functional network connectivity. Hence, changing the collective oscillatory state of the system—which need not be entirely determined by the anatomical network—qualitatively changes the distributed effects of focal perturbations, and alters the relations between those effects and measures of either structural or dynamical organization. In sum, we use a simplfied, large-scale computational model to highlight that the effects of regional stimulation can depend both on the location of the perturbed site and on the global state of ongoing brain network dynamics. Though currently idealized, extending the reduced model to incorporate further biological realism and empirical constraints is an exciting direction for future work attempting to directly compare against experimental findings.

## Materials and methods

### Acquisition of empirical human structural brain data

Human anatomical brain networks were reconstructed by applying deterministic tractography algorithms to diffusion-weighted MRI. In this study, we used a group-representative composite network assembled from 30 subject-level networks [54–56]. The mean age of participants was 26.2 years, the standard deviation was 5.7 years, and 14 of the subjects were female. To map anatomical networks, diffusion spectrum and T1-weighted anatomical images were acquired for each individual. For the DSI scans, 257 directions were sampled using a Q5 half-shell acquisition scheme with a maximum *b*-value of 5000 s/mm^2^ and an isotropic voxel size of 2.4 mm. We used an axial acquisition with repetition time TR = 5 seconds, echo time TE = 138 ms, 52 slices, and field of view of [231, 231, 125]mm. The T1 sequences used a voxel size of [0.9, 0.9, 1.0]mm, repetition time TR = 1.85 seconds, echo time TE = 4ms, and field of view of [240, 180, 160]mm. This data was initially collected for an earlier study [57], and was first published in [58]. The same data has also been used in several other prior investigations (e.g., [54, 56, 59]).

**Fig 1:**
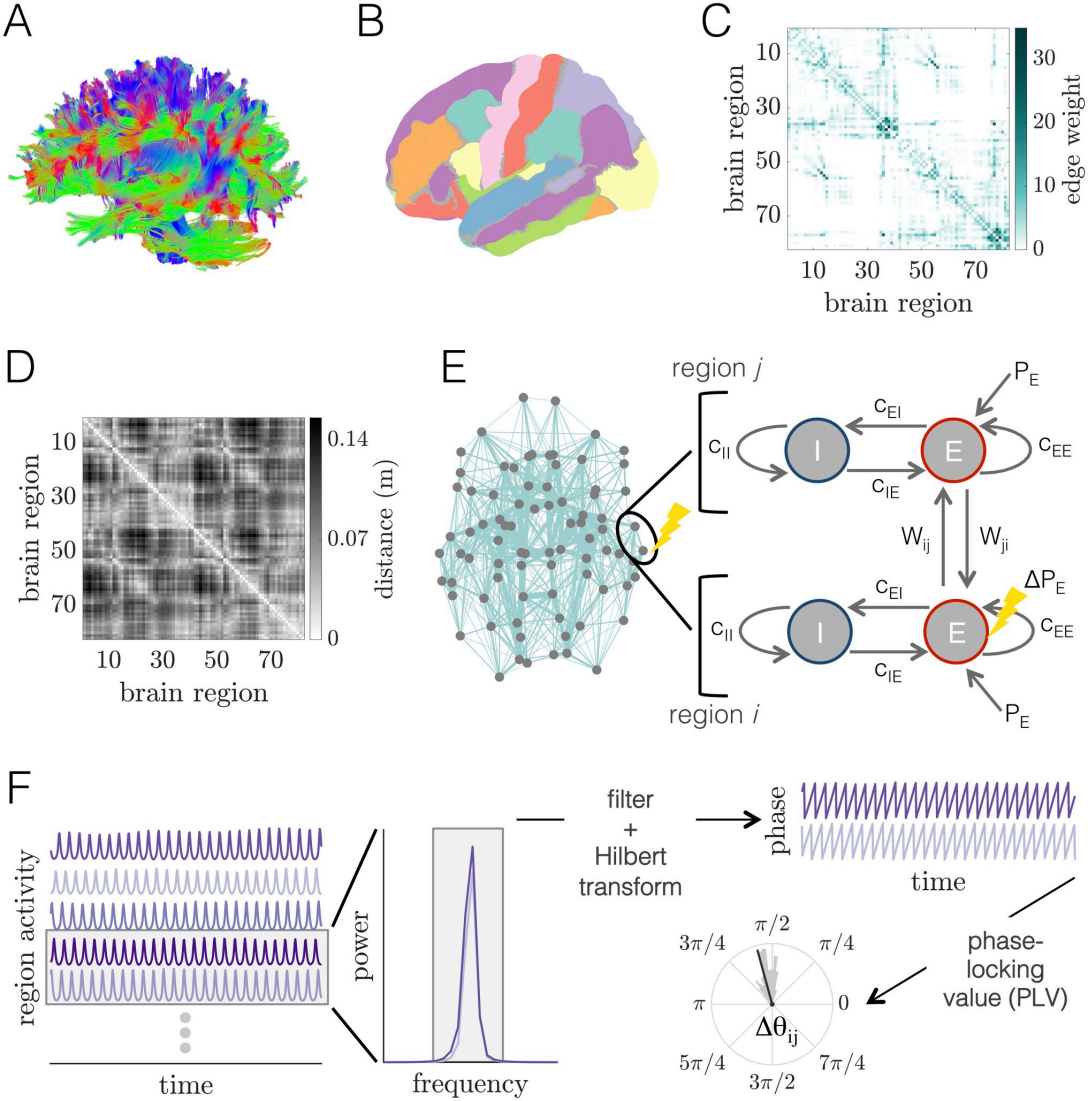
Whole-brain imaging data, computational model of large-scale brain dynamics, and schematic of analysis. *(A)* An example of white matter streamlines reconstructed from diffusion imaging and tractography of a human brain. *(B)* Noninvasive magnetic resonance imaging scans of human brain anatomy are used to segment the cortex and subcortex into 82 regions. *(C)* Adjacency matrix for a group-averaged structural brain network. Individual brain areas are represented as network nodes, and normalized white matter streamline counts between region pairs are represented as weighted network edges. *(D)* Matrix of Euclidean distances between the centers of mass of all region pairs. *(E)* Left: Structural brain network representation; location of gray circles correspond to region centers of mass, and teal lines show the strongest 20% of interareal connections, with line thickness proportional to connection strength. The two encircled nodes correspond to an unperturbed region *j* and an excited region *i* in the large-scale brain network, with the perturbed region indicated by the yellow lightning bolt. Right: Schematic of the computational model of large-scale brain dynamics. The activity of a given brain region *j* is modeled as a Wilson-Cowan neural mass, composed of interacting populations of excitatory *E* and inhibitory *I* neurons. Neural masses are then coupled through their excitatory pools according to the structure of the anatomical brain network. A perturbation to region *i* (pictorially represented with the lightning bolt) is modeled as an increase in its excitatory input from *P*_*E*_ → *P*_*E*_ + Δ*P*_*E*_. *(F)* The computational model generates oscillatory time-series of neural population activity for each brain region. These time-series can then be analyzed in Fourier space to determine relevant frequency bands for further analysis. After filtering time-series within the same frequency band of interest, functional interactions between brain region pairs are determined by extracting phase variables from each region’s filtered activity via the Hilbert transform, and then computing the phase-locking value to assess the consistency of phase relations over time and trials.

DSI Studio (www.dsi-studio.labsolver.org) was used to reconstruct DSI data using *q*-space diffeomorphic reconstruction (QSDR) [60], which reconstructs diffusion-weighted images in native space and computes the quantitative anisotropy (QA) of each voxel. Using the statistical parametric mapping nonlinear registration algorithm [61], the image is then warped to a template QA volume in Montreal Neurological Institute (MNI) space. Finally, spin-density functions were reconstructed with a mean diffusion distance of 1.25 mm with three fiber orientations per voxel. A modified FACT algorithm [62] was then used to perform deterministic fiber tracking with an angular cutoff of 55°, step size of 1.0 mm, minimum length of 10 mm, spin density function smoothing of 0.00, maximum length of 400 mm, and a QA threshold determined by DWI signal in the colony-stimulating factor [54–56, 58, 59, 63]. The algorithm terminated when 1,000,000 streamlines were reconstructed for each individual [54–56, 58, 59, 63] (Fig 1A).

T1 anatomical scans were segmented using FreeSurfer [64] and parcellated using the Connectome Mapping Toolkit (http://www.connectomics.org) according to an *N* = 82 area atlas [65] of 68 cortical and 14 subcortical areas (Fig 1B; Table A in S1 Text). The parcellation was registered to the *b*0 volume of each subject’s DSI data, and region labels were mapped from native space to MNI coordinates using a *b*0-to-MNI voxel mapping [54–56, 58, 59, 63]. While we use a relatively coarse-grained atlas, it aligns with atlas sizes used in other computational modeling studies (e.g., [35, 66–69]),and was chosen to reduce the computational costs of numerical simulations. However, we do mention limitations involved with this choice in the Discussion.

#### Ethics statement

All participants gave informed consent in writing and all protocols were authorized by the Institutional Review Board of the University of Pennsylvania.

### Network representation of anatomical brain data

To incorporate the structure of interareal connections into the model of large-scale brain activity, we represented the anatomical brain data as a network. This was achieved by first mapping each of the *N* = 82 regions to a unique node in a structural brain network **A** (see Table A in S1 Text for the mapping between node numbering and brain region labels). The structural edge weight *A*_*ij*_ between two brain areas (nodes) *i* and *j* was then defined as the total number of streamlines between the two areas divided by the geometric mean of their volumes [54, 56]. Note that due to limitations in the non-invasive techniques available for constructing human connectomes [70], the resulting structural network is weighted but undirected. Because the brain is a spatially-embedded system [71], each region *i* also has a location **r**_*i*_ = (*x*_*i*_, *y*_*i*_, *z*_*i*_) in real space. In the network representation, we defined the location of each node to be the center of mass of the corresponding region, allowing us to calculate matrix elements *D*_*ij*_ representing the Euclidean distance between nodes *i* and *j*. Assuming a fixed conduction speed, these interareal distances are then used to approximate time delays for signal transmission in the computational model [35, 66, 67, 72, 73].

In this study, we report results using a group-representative structural brain network derived by combining individual brain networks across multiple subjects. We used a previously-established consensus method for constructing the group representative network that preserves both the average binary connection density of the individual brain networks, as well as the approximate edge-length distribution of intra- and inter-hemispheric connections [54]. More details on this pooling procedure can be found in [55]. A group-representative interareal distance matrix was constructed by averaging the pairwise Euclidean distance matrices across subjects. In what follows, we assume that **A** (or *A*_*ij*_) refers to the group-representative structural brain network, and that **D** (or *D*_*ij*_) refers to the group-averaged interareal distance matrix. We show the group-representative anatomical connectivity matrix in Fig 1C, and we show the group-averaged distance matrix in Fig 1D.

### Biophysical model of large-scale brain dynamics

To model large-scale brain dynamics, we use a biophysically-motivated approach in which simulated activity is generated by a network of interacting neural masses [51]. In particular, the activity of each brain area is modeled as a Wilson-Cowan (WC) neural mass [53] and individual units are coupled according to the empirically-derived anatomical network. Importantly, these types of whole-brain computational models—which integrate non-linear, mean-field population dynamics with structural connectome architecture—have been utilized in a number of past efforts to gain insight into diverse neural phenomena [35, 66, 67, 69, 72–83].

Here, we employ such an approach to conduct a basic examination of how localized (regional) changes in neural activity affect dynamics across the brain. We offer a schematic of the model in Fig 1E. On the left, we show the structural brain network in real space. We focus on the two interconnected regions *i* and *j* encircled in black, of which the lower one (*i*) receives additional excitation (as denoted by the yellow lightning bolt). On the right, we show the setup of the coupled WC system for these two units. In the WC model, the activity of a particular brain region is defined by a coupled system of excitatory (*E*) and inhibitory (*I*) neuronal populations, and the dynamical variables are the mean firing rates of the *E* and *I* pools. The time-evolution of the average firing rates are in general governed by both intrinsic properties of the populations in a single region, as well as delayed, long-range input from other areas as dictated by the pattern of anatomical connectivity. In line with several previous studies [35, 66, 73–77, 81], we consider long-range connections to couple only the excitatory subpopulations of distinct brain areas.

The dynamics of the *j*^*th*^ brain area are governed by the following set of coupled differential equations:

$$\begin{array}{c} \begin{array}{cc} \tau_{E}\frac{dE_{j}\left(t\right)}{dt}= & -E_{j}\left(t\right)+\left[1-E_{j}\left(t\right)\right]S_{E}[c_{EE}E_{j}\left(t\right)-c_{IE}I_{j}\left(t\right)\\ & +C\sum_{i}W_{ij}E_{i}\left(t-\tau_{ij}\right)+P_{E,j}]+\sigma_{E}\xi \left(t\right) \end{array} \end{array}$$ (1)

and

$$\begin{array}{c} \begin{array}{cc} \tau_{I}\frac{dI_{j}\left(t\right)}{dt}= & -I_{j}\left(t\right)+\left[1-I_{j}\left(t\right)\right]S_{I}[c_{EI}E_{j}\left(t\right)-c_{II}I_{j}\left(t\right)\\ & +P_{I,j}]+\sigma_{I}\xi \left(t\right). \end{array} \end{array}$$ (2)

The variables *E*_*j*_(*t*) and *I*_*j*_(*t*) correspond to the firing rates of the excitatory and inhibitory populations of region *j*, and *τ*_*E*_ and *τ*_*I*_ are the excitatory and inhibitory time constants, respectively. The non-linear activation functions $S_{E}$ and $S_{I}$ of the excitatory and inhibitory pools are given by the sigmoidals

$$\begin{array}{c} S_{E}\left(x\right)=\frac{1}{1+e^{-a_{E}\left(x-\mu_{E}\right)}} \end{array}$$ (3)

and

$$\begin{array}{c} S_{I}\left(x\right)=\frac{1}{1+e^{-a_{I}\left(x-\mu_{I}\right)}}. \end{array}$$ (4)

The quantities *μ*_*E*_ and *μ*_*I*_ give the mean firing thresholds for each subpopulation, and the gain parameters *a*_*E*_ and *a*_*I*_ set the spread of the firing thresholds for the two groups.

Dynamics of the excitatory ensemble are driven by *(1)* the local interaction strength within the excitatory population *c*_*EE*_, *(2)* the interaction strength from the inhibitory population to the excitatory population *c*_*IE*_, *(3)* constant, non-specific background drive *P*_*E*,*j*_, and also *(4)* interactions *W*_*ij*_ corresponding to long-range excitatory inputs from different populations *i* that link to unit *j* via anatomical connectivity. Following [75–77, 84], we let $W_{ij}=\frac{A_{ij}}{\sum_{i}A_{ij}}$, which is simply the connection weight from *i* to *j*, normalized by the total input to region *j*. Furthermore, *C* is a global coupling that tunes the overall interaction strength between different brain areas, and *τ*_*ij*_ is a time delay between regions *i* and *j* that arises due to the spatial embedding of the brain network and the fact that signal transmission speeds are finite [35, 66, 67, 69, 72, 73]. We set $\tau_{ij}=\frac{D_{ij}}{v}$, where *D*_*ij*_ is the Euclidean distance between regions *i* and *j* and *v* is a constant signal conduction speed. Activity in the inhibitory ensemble depends on *(1)* the interaction strength *c*_*EI*_ from the excitatory population, *(2)* the local interaction strength within the inhibitory population *c*_*II*_, and *(3)* other possible non-specific inputs *P*_*I*,*j*_. Finally, to increase biological plausibility and incorporate the stochastic nature of neural dynamics, we add a term *σ*_*E*_ *ξ*(*t*) to Eq 1 and a term *σ*_*I*_ *ξ*(*t*) to Eq 2, which correspond to Gaussian white noise with zero mean and standard deviations *σ*_*E*_ and *σ*_*I*_, respectively [35, 73]. In what follows, we will take the excitatory population activities *E*_*j*_(*t*) of each brain area as the observables of interest [35, 66, 75, 76, 83].

#### Model parameters

Under appropriate parameter choices, the WC model can give rise to oscillatory dynamics [53]. Such rhythmic activity is ubiquitous in large-scale neural systems [1] and is the dynamical behavior of interest for this investigation. While oscillation frequencies observed in neural systems can span orders of magnitude [1], local neuronal populations often exhibit gamma band (30-90Hz) rhythms as a result of feedback between coupled excitatory and inhibitory neurons [4, 85, 86]. Furthermore, gamma oscillations and synchronization between distributed brain areas are associated with the flow of information between neuronal ensembles [10, 87, 88], are modulated by stimuli [15, 16], and are thought to underlie a number of cognitive processes [6]. Because gamma oscillations are robustly observed in excitatory-inhibitory circuits, we set parameters in the phenomenological WC model such that individual brain regions oscillate in the gamma band when coupled [73] (see Table 1). We also note that it may be interesting in future work to investigate other frequency bands or multiple frequency bands simultaneously [89].

**Table 1:** Parameter values for the large-scale Wilson-Cowan neural mass model and for the numerical simulations.

Parameter	Description	Value
*v*	propagation speed	10m/s
*C*	global coupling strength	0–5
*τ*_*E*_	excitatory time constant	2.5ms
*τ*_*I*_	inhibitory time constant	3.75ms
*a*_*E*_	excitatory gain	1.5
*a*_*I*_	inhibitory gain	1.5
*μ*_*E*_	excitatory firing threshold	3.0
*μ*_*I*_	inhibitory firing threshold	3.0
*c*_*EE*_	local E to E coupling	16
*c*_*IE*_	local I to E coupling	12
*c*_*EI*_	local E to I coupling	15
*c*_*II*_	local I to I coupling	3
$P_{E}^{\text{base}}$	baseline excitatory background drive	0.5–0.85
Δ*P*_*E*_	perturbation strength	0.1
*P*_*I*_	inhibitory background drive	0
*σ*_*E*_	excitatory noise strength	5 × 10^−5^
*σ*_*I*_	inhibitory noise strength	5 × 10^−5^
*dt*	integration time step	5 × 10^−5^s
*dt*_ds_	downsampled time step	1 × 10^−3^s

As discussed further in Sec. SI of S1 Text, the non-specific background input *P*_*E*_ is the typical control parameter used to tune the behavior of an isolated WC unit. At low values of *P*_*E*_, a single WC unit flows towards a low-activity steady-state (Fig. A, panel A in S1 Text), and at high values of *P*_*E*_, the system reaches a stable high-activity steady-state (Fig. A, panel C in S1 Text). At intermediate values of the excitatory drive, an isolated unit—with the parameters given in Table 1—will undergo a bifurcation and exhibit rhythmic activity in the gamma frequency band (Fig. A, panel B in S1 Text). Up to a point, increasing *P*_*E*_ within this intermediate region leads to oscillations with increasing amplitude and frequency (Fig. A, panels D–F in S1 Text).

The situation becomes more complex when multiple WC units are coupled via the structural connectome. In this scenario, an individual region’s dynamics are determined by a combination of the constant drive *P*_*E*_ and the strength of delayed inputs from other parts of the network, which are modulated by the coupling *C* and the structural connectivity **A**. To account for these two influences, we consider both *P*_*E*_ and *C* as tuning parameters, and examine working points at which the combination of *P*_*E*_ and *C* generate oscillatory activity in individual brain areas. Finally, we set the signal propagation speed to a fixed value of *v* = 10*m*/*s*, which is in the range of empirical observations and previous large-scale modeling efforts [35].

#### Incorporating local perturbations into the large-scale model

The baseline condition of the network corresponds to the situation in which all brain areas receive the same level of background drive, such that $P_{E,j}=P_{E}^{\text{base}}$ for all *j* ∈ {1, …, *N*}. To investigate how regional perturbations affect brain-wide dynamics, we examine the effects of increased excitation to a single brain area. This is modeled as a selective increase in drive to the excitatory population of the perturbed neural mass *i* such that *P*_*E*,*i*_ → *P*_*E*,*i*_ + Δ*P*_*E*_, where Δ*P*_*E*_ > 0 denotes the strength of the perturbation [35] (see Fig 1E for a schematic). The dynamics of the system in the baseline state can then be compared to the situation in which unit *i* receives additional input (i.e., where we have $P_{E,i}=P_{E}^{\text{base}}+\Delta P_{E}$ and $P_{E,j}=P_{E}^{\text{base}}$ for all *j* ≠ *i*).

We note that, phenomenologically, excitation of a given brain area could occur through a number of mechanisms, including sensory input to primary sensory regions, brain stimulation, or, alternatively, via internal processes that regulate inputs to or excitability levels of specific neuronal populations. The goal of this work is to study the effects of localized excitations generally, rather than to design a detailed model of a specific type of perturbation. For this reason, we choose to study the simplest case of constant excitation.

### Numerical methods and simulations

The equations governing the time evolution of the excitatory and inhibitory population activities form a system of coupled stochastic, delayed differential equations. We numerically integrate this system using the Euler-Mayurama method with a time step of *dt* = 5 × 10^−5^s. For the time delays, we round each *τ*_*ij*_ to the nearest multiple of the integration time step *dt*, and for the initial conditions, we assume a constant history for each unit’s activity of length equal to the longest delay in the system. After running a simulation, we discard the first *t*_burn_ = 1 second so that our analysis is not biased by transients or the specific choice of initial conditions. Each time-series is then downsampled to a resolution of *dt*_ds_ = 1 × 10^−3^s. The parameters for the numerical simulations are shown in Table 1.

### Power spectra

Useful characteristics of the simulated activity are apparent in the frequency domain (see Fig 1F). Here, we use Welch’s method (as implemented in MATLAB R2019a) to estimate the power spectral density (psd) of the excitatory population activities. We use window sizes of 1 second with 50% overlap, and subtract the mean of each time-series before computing the psd.

### Quantifying interareal phase-locking

To quantify the extent of temporal coordination between different brain areas, we use the phase-locking value (PLV) [90]. This measure is commonly used to assess the level of coherence between phases in a given frequency band. Importantly, because the state variables in the WC model are real-valued signals with possibly multiple spectral components, we compute PLVs for a given frequency band by *(1)* filtering all raw excitatory time-series within the same specified frequency range, and *(2)* extracting instantaneous phases for the given frequency band using the Hilbert transform (see Fig 1F). In the following two sections, we describe these steps in more detail.

#### Instantaneous phases from the Hilbert transform

Given a real-valued signal *X*(*t*), it is possible to define instantaneous phase and amplitude variables that describe the signal using the Hilbert transform. Importantly, although the Hilbert transform can theoretically be computed for an arbitrary signal *X*(*t*), the instantaneous amplitude *A*(*t*) and phase *θ*(*t*) are only physically meaningful for relatively narrowband signals [91]. It is therefore necessary to filter a signal before taking the Hilbert transform. Here, raw time-series were bandpass filtered in a frequency range *f*_*o*_ ± Δ*f* Hz using a 6th-order Butterworth filter in the forward and backward directions. In the results section, we describe how *f*_*o*_ and Δ*f* are determined during the presentation of various findings that depend on computing the Hilbert phase. Filtering was carried out in MATLAB using the ‘butter’ and ‘filtfilt’ functions. After filtering the simulated activity, the Hilbert transform was applied to extract instantaneous phases for the given frequency band. The Hilbert transform was implemented using the ‘hilbert’ function in MATLAB. More details on the Hilbert transform can be found in Sec. SXIII of S1 Text.

#### Functional connectivity from the phase-locking value

The outputs of the filtering and Hilbert transform processes described in the previous section are instantaneous phases *θ*_*i*_(*f*_*o*_, *t*) derived from the excitatory activity *E*_*i*_(*t*) of each brain region *i* at a given central frequency *f*_*o*_ and time *t* (Fig 1F). From these phases, we can quantify the extent of phase-coherence between brain areas’ signals in a given frequency band using the phase-locking value (PLV); see Fig 1F. The PLV—here denoted symbolically as *ρ*_*ij*_—between two phase time-series *θ*_*i*_(*t*) and *θ*_*j*_(*t*) is given by

$$\begin{array}{c} \rho_{ij}=|\frac{1}{T_{s}}\sum_{t=1}^{T_{s}}e^{i[\theta_{i}\left(t\right)-\theta_{j}\left(t\right)]}|, \end{array}$$ (5)

where *T*_*s*_ is the number of sample time points over which the phase-locking is computed. If the phase difference Δ*θ*_*ij*_(*t*) = *θ*_*i*_(*t*) − *θ*_*j*_(*t*) is constant over a given time window, *ρ*_*ij*_ will be equal to 1, whereas if the phase-differences are distributed uniformly, *ρ*_*ij*_ will be approximately 0; in this way, *ρ*_*ij*_ ∈ [0, 1].

We would also like to ensure that the PLV reflects the consistency of phase relations that arise from interactions (direct or indirect), and not locking arising from the fact that two regions happen to have the same frequency, but, possibly, a different phase relation in every trial. We therefore concatenate phase time-series from different trials before computing the PLV [92], where each trial is a simulation run with different random initial conditions and noise realizations. Accordingly, a high PLV indicates that across time *and* trials, the activity of the corresponding regions exhibits a consistent phase relationship within a particular frequency band.

As with structural connectivity, it is useful to think of a given *N* × *N* matrix of PLV values as a network where the element (edge) *ρ*_*ij*_ is the phase-coherence between region (node) *i* and region (node) *j*. In contrast to the structural network, this PLV-based network represents the presence of functional associations between brain regions’ activity. Following common terminology, we will thus often refer to phase-locking as “functional connectivity” and phase-locking matrices as “functional networks”.

### Statistical analyses

All data and statistical analysis was performed in MATLAB release R2019a. Statistical dependencies between two variables were assessed via the Spearman rank correlation, using the built-in MATLAB function ‘corr’. Throughout the text, we denote the Spearman correlation coefficient as *r*_*s*_. Rank correlations are considered statistically different from zero if the corresponding *p*-value is less than 0.05.

### Summary of computed quantities

Throughout the text we compute a number of different measures to characterize the behavior of the system at baseline and under focal perturbation. To aid the readability of the manuscript, we list these quantities in Table 2 with a brief summary. The measures are listed according to the section in which they first appear.

**Table 2:** List of computed measures.

Measure	Description
$\langle \bar{E(t)}\rangle$	time- and network-averaged firing rate
〈*f*_peak_〉	network-averaged peak frequency
$P_{E}^{*}\left(C\right)$	background drive at which oscillations emerge for a given coupling *C*
*ρ*_*ij*_	PLV between units *i* and *j* at baseline
*ρ*^global^	global phase-locking order parameter
*ρ*^local^	local phase-locking order parameter
$f_{i}^{\text{peak}}$	peak frequency of node *i* at baseline
$f_{i,\delta_{i}}^{\text{peak}}$	peak frequency of node *i* when stimulated
$\Delta f_{i,\delta_{i}}^{\text{peak}}$	change in peak frequency of node *i* when stimulated
〈psd〉_*j*≠*i*_	power spectra averaged over all units *j* ≠ *i*
${\langle \Delta {\text{psd}}_{j,\delta_{i}}\rangle }_{j\neq i}$	change in psd of node *j* induced by excitation of node *i*, averaged over all nodes *j* ≠ *i*
$s_{i}^{\text{struc}}$	structural strength of node *i*
$s_{i}^{\text{func}}$	functional strenth of node *i*
$\langle |\Delta \rho_{\delta_{i}}^{\text{base}}|\rangle$	average absolute change in baseline-band PLV induced by stimulation of node *i*
$\langle |\Delta \rho_{\delta_{i}}^{\text{exc}}|\rangle$	average absolute change in excited-band PLV induced by stimulation of node *i*
〈|Δ*ρ*^base^|〉	mean of the distribution of average absolute changes in baseline-band PLV induced by stimulation of each node
〈|Δ*ρ*^exc^|〉	mean of the distribution of average absolute changes in excited-band PLV induced by stimulation of each node
$\text{CoV}[\langle |\Delta \rho_{\delta_{i}}^{\text{base}}\left|\rangle \right]$	coefficient of variation of the distribution of average absolute changes in baseline-band PLV induced by stimulation of each node
$\text{std}[\langle |\Delta \rho_{\delta_{i}}^{\text{exc}}\left|\rangle \right]$	standard deviation of the distribution of average absolute changes in excited-band PLV induced by stimulation of each node

## Results

### Baseline dynamical regimes of the brain network model

Depending on the values of various parameters, the brain network model exhibits different qualitative behaviors. Importantly, different baseline states may in turn result in distinct modulations of brain-wide activity patterns in response to local perturbations. We thus begin by characterizing the behavior of the system at baseline (i.e. in the absence of regional stimulation). This initial study will provide context for our subsequent investigations examining how the effects of focal excitations depend upon the system’s baseline state.

We focus on two parameters of interest: *(1)* the level of generic background input to the excitatory populations $P_{E}^{\text{base}}$ and *(2)* the global coupling strength *C*. Recall that for an isolated WC unit, $P_{E}^{\text{base}}$ is a bifurcation parameter that transitions population activity between a quiescent and an oscillatory state [53, 93]. However, when examining a network of coupled neural masses, the dynamics of each element are also dictated by inputs from other units in the system. The parameter *C* is a second control parameter that globally scales the interaction strength between brain areas by tuning how much input a given region receives from its neighbors in the network. The nature of both local and network-wide dynamical behaviors will thus change depending on the combination of $P_{E}^{\text{base}}$ and *C*, allowing the system to exist in markedly different states. Though the tuning parameters in the network model are phenomenological, from a biological standpoint, global changes in these parameters could represent, for example, the effects of neuromodulation [94–96]—which exerts widespread influences across the brain [97]—or changes in brain state more generically.

To quantify how model behavior varies as a function of $P_{E}^{\text{base}}$ and *C*, we perform a sweep over a broad range of these parameters, considering values of $P_{E}^{\text{base}}\in \left[0.5,0.85\right]$ in steps of $\Delta P_{E}^{\text{base}}=0.05$, and values of *C* ∈ [0, 5] in steps of Δ*C* = 0.1. These ranges were chosen to allow for the exploration of multiple oscillatory regimes of the system. For each parameter combination, we run five, 2-second-long simulations. The values of all other parameters are defined in Table 1, with the exception that, for these sweeps, we run noiseless simulations in order to more precisely demarcate the boundaries between different dynamical modes of the model.

#### Long-range coupling strength and background drive tune baseline dynamical state

**Fig 2:**
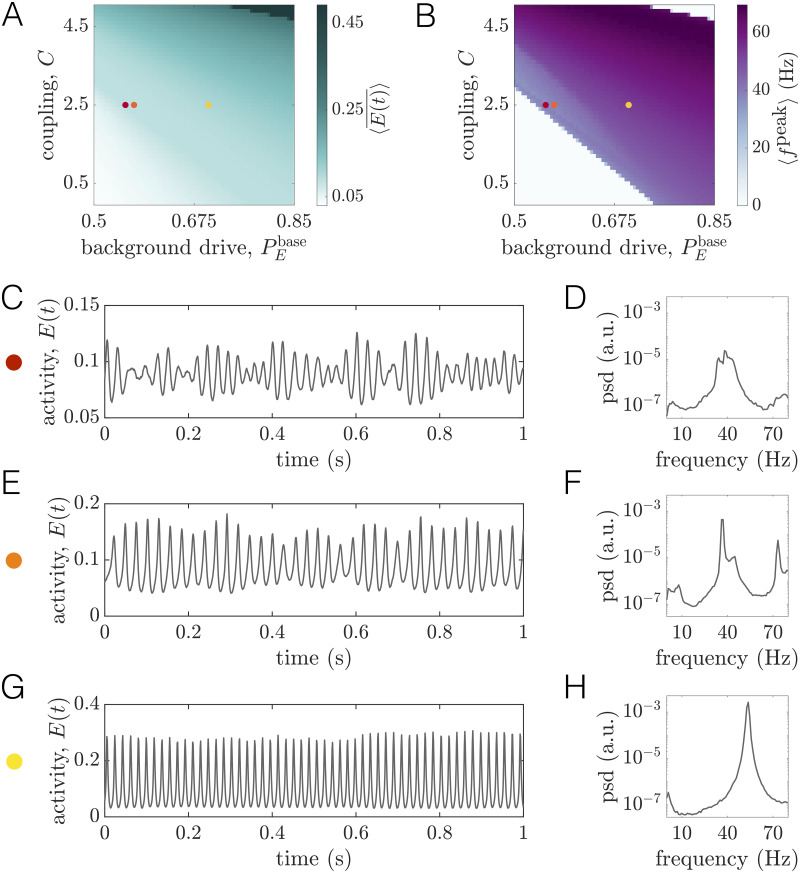
Long-range coupling strength *C* and background drive $P_{E}^{\text{base}}$ modulate firing rates and oscillation frequencies at baseline. *(A)* The time- and network-averaged population firing rate $\langle \bar{E(t)}\rangle$ as a function of *C* and $P_{E}^{\text{base}}$ (units are arbitrary). *(B)* The network-averaged peak frequency of regional activity 〈*f*^peak^〉 as a function of *C* and $P_{E}^{\text{base}}$. *(C)* A segment of the activity of one brain area and *(D)* the corresponding power spectra of the same area at the working point denoted by the red dot in panels *A* and *B* ($P_{E}^{\text{base}}=0.553$, *C* = 2.5). *(E)* A segment of the activity of one brain area and *(F)* the corresponding power spectra of the same area at the working point denoted by the orange dot in panels *A* and *B* ($P_{E}^{\text{base}}=0.57$, *C* = 2.5). *(G)* A segment of the activity of one brain area and *(H)* the corresponding power spectra of the same area at the working point denoted by the yellow dot in panels *A* and *B* ($P_{E}^{\text{base}}=0.7$, *C* = 2.5).

We begin by computing two measures that quantify regional dynamics: *(1)* the time-averaged firing rate $\bar{E(t)}$, and *(2)* the frequency at maximum power (peak frequency) *f*^peak^ of a given region. To obtain summary measures characterizing the state of the system as a whole, we compute network-averages of these quantities, denoted by angled-brackets. In studying $\langle \bar{E(t)}\rangle$ as a function of $P_{E}^{\text{base}}$ and *C*, we observe three principal regimes (Fig 2A). When both $P_{E}^{\text{base}}$ and *C* are low, the system settles to a state of low average firing rate (white region); this state corresponds to a non-oscillatory, low-activity equilibrium. In contrast, when $P_{E}^{\text{base}}$ and *C* are both high, the average firing rate saturates at a high level (dark green region); this state corresponds to a non-oscillatory, high-activity equilibrium. Finally, at intermediate values of these parameters, the mean firing rate varies between the low and high extremes, and the regional activity is oscillatory; because we wish to consider the rhythmic nature of brain activity, this is the relevant portion of parameter space.

Next we seek to understand how 〈*f*^peak^〉 varies in the $P_{E}^{\text{base}}$—*C* plane (Fig 2B). A clear wedge-shaped area marks parameter combinations that give rise to network-averaged peak frequencies in the gamma range. As with the firing rate, the peak frequency tends to increase (decrease) with either increasing (decreasing) background excitation or coupling strength. By comparing Fig 2B to 2A, we see that the white areas surrounding the purple wedge correspond to the regions of parameter space where the firing saturates at a fixed low or high value. In Sec. SII of S1 Text, we describe a systematic method for determining boundaries in the 2D space spanned by *C* and $P_{E}^{\text{base}}$ that indicate the onset or disappearance of oscillatory activity (see Fig. B in S1 Text). In what follows, we use $P_{E}^{*}\left(C\right)$ to denote the level of background drive at which oscillations begin to emerge for a fixed coupling strength *C*. We refer the reader to Sec. SII of S1 Text for a detailed description of how this value is determined from the simulations. Furthermore, we often plot quantities as functions of the relative drive $P_{E}^{\text{base}}-P_{E}^{*}\left(C\right)$, such that $P_{E}^{\text{base}}-P_{E}^{*}\left(C\right)=0$ indicates the transition point from a low-activity state to an oscillatory state at a coupling *C*.

To provide further intuition for how dynamics vary within this parameter space, we study example time-series and power spectra for three different baseline states (colored dots in Fig 2A and 2B). Note that these working points correspond to an intermediate coupling value of *C* = 2.5, but varying levels of the constant baseline input $P_{E}^{\text{base}}$. We begin with the working point $P_{E}^{\text{base}}=0.553$, which sits just beyond the boundary indicating the transition to sustained rhythmic activity. From the time-series, we observe that the activity is oscillating (Fig 2C), and the spectra indicates a peak frequency of ≈40Hz on a broadband background (Fig 2D). We next consider the working point $P_{E}^{\text{base}}=0.57$. In this state, each unit receives slightly more drive, leading to higher-amplitude oscillations (Fig 2E and 2F). However, although peak spectral power increases, amplitude modulations can still be seen in the corresponding time-series (Fig 2E). Finally, we consider the working point $P_{E}^{\text{base}}=0.7$. Here, the activity is characterized by regular, high-amplitude oscillations (Fig 2G). Furthermore, inspection of the power spectra indicates a single, narrow peak at a slightly higher frequency than the previous working point (Fig 2H).

#### Global phase-coherence is non-monotonically modulated by coupling strength and background drive

Both the firing rate and the power spectra are measures that quantify the nature of individual regions’ activity. For networks, it is also imperative to define measures that capture information about the extent of dynamical order in the system as a whole. Indeed, for networks of coupled units, the system’s “state” is defined not only by the behavior of individual units, but also by how their dynamics are interrelated. Here, we are interested in the degree to which regional dynamics are coherent, which we quantify via the PLV between regions’ activities. To compute PLVs for baseline conditions, we begin by filtering the activity of each unit in the same, common frequency band. This band is determined by first finding the peak frequency of each unit at the given working point. Hence, we obtain a set of *N* values $\left\{f_{i}^{\text{peak}}\right\}$ corresponding to the peak frequencies of all units *i*∈ {1, …, *N*} at baseline. We then filter the activity of every region in a frequency band spanning 10Hz above the maximum peak frequency in the network ($\max\{f_{i}^{\text{peak}}\}$) and 10Hz below the minimum peak frequency in the network ($\min\{f_{i}^{\text{peak}}\}$). After identically filtering each unit’s activity in this common band, we extract Hilbert phases from the filtered signals. Finally, PLVs between all pairs of brain areas are computed according to Eq 5, using 50 different simulations (trials) of 5 seconds each (with noise included).

**Fig 3:**
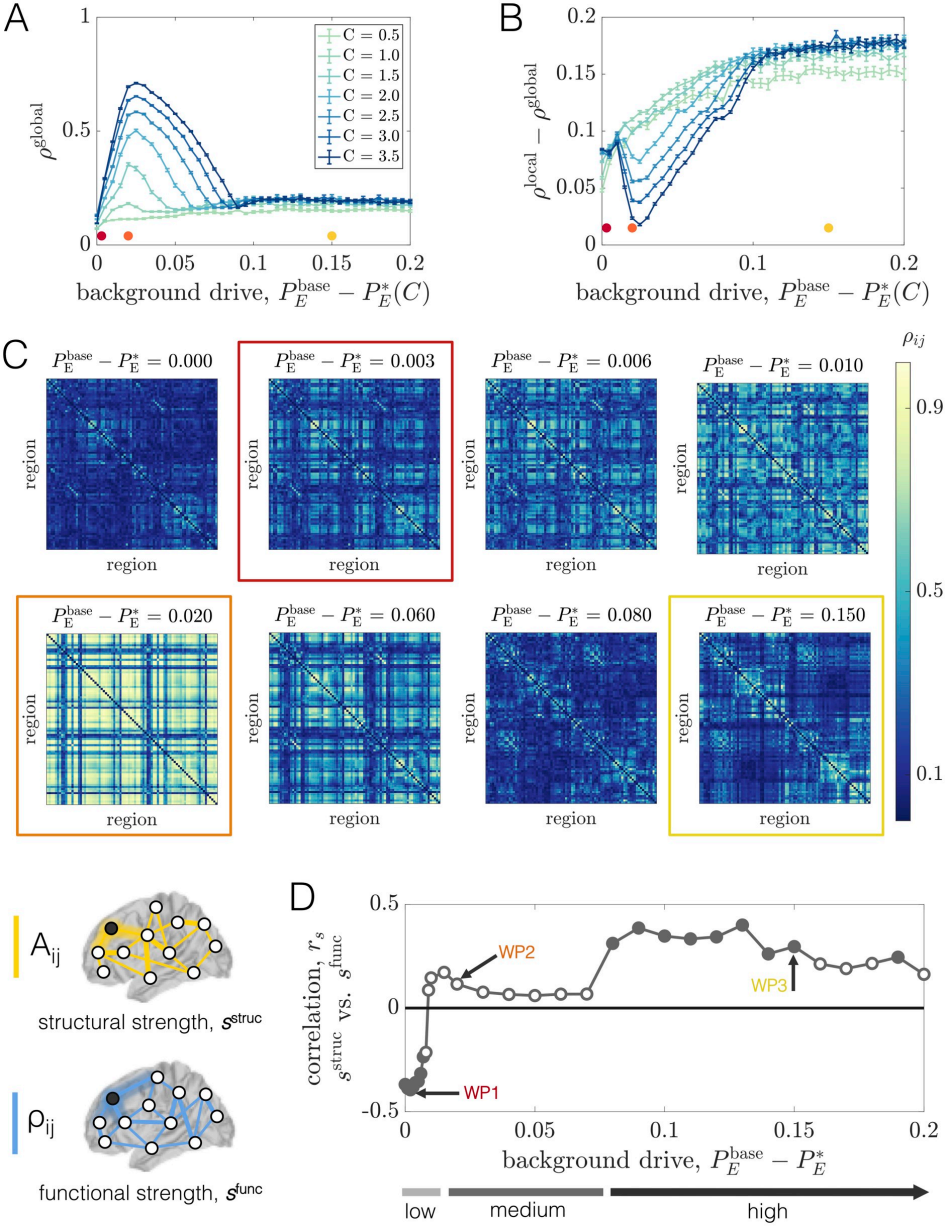
Long-range coupling strength *C* and background drive $P_{E}^{\text{base}}$ modulate network phase-coherence and relationships between structural and functional connectivity at baseline. *(A)* The global order parameter *ρ*^global^ *vs*. $P_{E}^{\text{base}}-P_{E}^{*}\left(C\right)$, for different fixed values of *C*. Error bars are estimated from 100 bootstrap samples of the simulations at each coupling and background drive, and correspond to ± one standard deviation of the bootstrap disribution of *ρ*^global^. *(B)* The difference between the global and local order parameters, *ρ*^global^ − *ρ*^local^, *vs*. $P_{E}^{\text{base}}-P_{E}^{*}\left(C\right)$, for different fixed values of *C*. Error bars are estimated from 100 bootstrap samples of the simulations at each coupling and background drive, and correspond to ± one standard deviation of the bootstrap distribution of *ρ*^global^ − *ρ*^local^. *(C)* Region-by-region PLV matrices for various values of $P_{E}^{\text{base}}-P_{E}^{*}\left(C\right)$ at fixed *C* = 2.5. The boxed matrices correspond to the red, orange, and yellow working points in Fig 2 and in panels A and B of this figure. *(D)* The Spearman correlation *r*_*s*_ between structural node strength *s*^struc^ and functional node strength *s*^func^ *vs*. $P_{E}^{\text{base}}-P_{E}^{*}$ at fixed *C* = 2.5. Empty circles indicate that the correlation was not statistically significant at the *p* = 0.05 level. The arrows mark three different working points—WP1, WP2, and WP3 (which correspond to the red, orange, and yellow dots/boxes in this figure)—that will be studied in detail.

To summarize how the overall level of coherence in the network varies as a function of the background drive and coupling strength, we defined a macroscopic order parameter as the average of the PLVs over all pairs of units in the network: *ρ*^global^ = 〈*ρ*_ij_〉. This quantity ranges between 0 and 1, where larger values indicate a more dynamically ordered state of the network. In general, we find that the background input and the coupling strength interdependently tune the level of coherence in the system (Fig 3A). At low coupling, brain areas cannot coordinate their dynamics and *ρ*^global^ remains at a relatively low value for a range of drives. In contrast, as the coupling is increased, we begin to see a qualitative change in behavior. For higher values of *C*, we observe that *ρ*^global^ varies non-monotonically (first increasing and then decreasing) as a function of the (relative) background drive. For a given coupling *C*, there appears to be a “critical” value at relatively small but non-zero $P_{E}^{\text{base}}-P_{E}^{*}\left(C\right)$ where the system develops a well-defined peak in global coherence. As the drive is increased further, *ρ*^global^ begins to decrease and then eventually plateaus, albeit with some fluctuations. More specifically, at levels of background drive well beyond the state of peak coherence, *ρ*^global^ relaxes to an intermediate value between its peak and its value at the lowest background input. In this regime, the system resides in a state of partial order. Increasing the coupling has the effect of amplifying the maximum value of *ρ*^global^ (although *ρ*^global^ remains well below 1 for all couplings considered), but does not appear to significantly affect the order parameter to the right side of the peak.

To provide further intuition for this behavior, we focus on an intermediate coupling of *C* = 2.5 and examine the pairwise coherence patterns *ρ*_*ij*_ for several values of the background drive (Fig 3C). At the lowest (relative) baseline input ($P_{E}^{\text{base}}-P_{E}^{*}\left(C\right)=0$), some organization can be seen in the PLV matrix, but the system is weakly coherent overall. In this state, units exhibit relatively low amplitude oscillations, and are therefore more influenced by noise. It is thus reasonable to expect little phase-locking at low background drive. However, with only a small increase in the non-specific input (e.g., $P_{E}^{\text{base}}-P_{E}^{*}\left(C\right)=0.003$), we observe distributed increases in coherence and a large spread of high, medium, and low coherence pairs dispersed throughout the network. Increasing the background drive slightly more (e.g., $P_{E}^{\text{base}}-P_{E}^{*}\left(C\right)=0.02$) leads to the emergence of large, highly-coherent blocks that span the system. This working point sits near the peak of *ρ*^global^ and represents a highly ordered state of the system. As the background drive is increased further, though, phase-locking begins to decrease widely throughout the network and the coherence pattern markedly changes into a more segregated architecture. In particular, for high $P_{E}^{\text{base}}-P_{E}^{*}\left(C\right)$, we observe the emergence of smaller phase-locked clusters (Fig 3C, Row 3). To understand this shift in behavior, it is important to note that increasing $P_{E}^{\text{base}}$ increases the extent to which regional activity is independently generated in each area *vs*. driven by long-range network interactions. The strengthening of regional oscillations and enhanced influence of local dynamics with increasing $P_{E}^{\text{base}}$ seems to eventually hinder the ability of units to adjust their rhythms and achieve widespread coherence. Note that phase-locking is also made especially difficult by the large variance in the distribution of interareal delays imposed by the connectome’s spatial embedding, and indeed, for high background drive conditions, more strongly connected and spatially nearby units are those able to maintain stronger coherence.

In general, our observations point to complex behavior in which the macroscopic order parameter varies non-monotonically as a function of the baseline input and network coupling strength (Fig 3A and 3C). Therefore, a variety of qualitatively different regimes exist, beyond just a simple binary separation into a disordered and ordered state. To more quantitatively distinguish network states before and after the point of peak coherence, we also considered a local order parameter $\rho^{\text{local}}=\sum_{ij}A_{ij}\rho_{ij}/\sum_{ij}A_{ij}$, which is a weighted average of *ρ*_*ij*_ with weights equal to the strength of structural network connections. In this way, *ρ*^local^ will be larger when more strongly connected brain areas are more phase-locked. In Fig 3B, we show *ρ*^local^ − *ρ*^global^ *vs*. $P_{E}^{\text{base}}-P_{E}^{*}\left(C\right)$ for different values of the coupling *C*. Beyond a certain point, the curves for all couplings exhibit a clear upward trend where the extent of local coherence increases relative to the extent of global coherence. This behavior indicates that the macroscopic state of the system becomes increasingly constrained by structure as the background drive increases. Hence, even though the level of global coherence can be similar to the left and right of peak *ρ*^global^, the system is in qualitatively different dynamical modes in the two regimes. Also note that for the higher couplings, *ρ*^local^ − *ρ*^global^ first decreases before consistently rising. This variation occurs because, for large enough coupling strengths, the level of global coherence is able to compete with the level of local coherence at background drives near peak *ρ*^global^.

As a final demonstration of the complexity of the structure-function landscape across operating points, we consider the relationship between brain areas’ structural and functional connectivity strengths as a function of $P_{E}^{\text{base}}-P_{E}^{*}\left(C\right)$ for a fixed coupling *C* = 2.5 (Fig 3D). The structural strength of node *j*, $s_{j}^{\text{struc}}=\sum_{i=1}^{N}A_{ij}$, is a common measure of a brain area’s importance in an anatomical network [98]. Similarly, the (baseline) functional strength of node *j*, $s_{j}^{\text{func}}=\sum_{i=1}^{N}\rho_{ij}$, quantifies how dynamically integrated that region is to the network as a whole. From Fig 3D, we observe that shifting the system’s working point can drastically alter how—and the extent to which—structural strength and functional strength are related. Specifically, while there tends to be a weak positive correlation between *s*^struc^ and *s*^func^ at high background drives (e.g. at WP3), the correlation disappears (e.g. at WP2) and then reverses in sign (e.g. at WP1) as the background drive is lowered. Critically, these transitions occur in the absence of any change to the anatomical connectome, and are instead driven by a global change in the behavior of brain areas’ dynamics (induced by changing the level of background input). Also note that when the correlations are significant, they are intermediately-valued. Together, these results indicate that while a given structural network may only be able to support specific patterns of coordinated activity, the relationships between the two are not trivial and are modulated by dynamic properties [99, 100]. In general, functional connectivity thus reflects a complex interplay between both anatomical connectivity and the system’s dynamical state.

It is crucial to remark that the behaviors seen here are more diverse than what tends to occur in simpler phase-oscillator models, where coupling strength is the main control parameter and typically induces a monotonic increase in synchrony. A critical difference between phase-based models and the more realistic WC model considered here is that, for the latter case, unit dynamics are described and coupled by real-valued signals that represent regional activity. Hence, widespread changes in the amplitude or stability of areas’ dynamics (in addition to changes in coupling strength) can affect the macroscopic state of the network. Indeed, the preceding analyses show that global modulations in the level of diffuse, constant input to the neural populations can push the system into very different oscillatory modes, beyond just a steady progression from an incoherent to a coherent state. In what follows, we will exploit this behavior to examine how the effects of focal perturbations depend not only on which region is targeted, but also on the baseline working point of brain network dynamics as a whole.

### Effects of regional perturbations on brain network activity and dependence on dynamical state

To investigate how local perturbations modulate brain network dynamics, and specifically how the effects may depend on the system’s collective state, we begin with an in-depth examination of three distinct working points. In particular, we focus on a fixed intermediate coupling strength *C* = 2.5 for which the system exhibits a clear peak in *ρ*^global^ (Fig 3A). We then examine two values of the background drive $P_{E}^{\text{base}}$ that place the system either in a state preceding (WP1) or following (WP3) the global coherence peak. In Sec. SIII of S1 Text, we also present results for a state in which the system is approximately at peak global coherence (WP2). We then proceed to more generally characterize the global impacts of stimulation as the background drive is varied across a wide range. Throughout the text, stimulation of a single brain area *i* is introduced by increasing its excitatory input by an amount Δ*P*_*E*,*i*_ = 0.1, while keeping all other regions at their working-point-specific baseline drive. Finally, in S1 Text, we verify that results hold for different values of $P_{E}^{\text{base}}$ in the vicinity of those studied in the main text (Sec. SVIII), we examine the effects of varying the perturbation strength (Sec. SX), and we consider an alternative value of the global coupling (Sec. SXI). Note that our goal is not to exhaustively analyze all possible parameter combinations, but rather to demonstrate that the network response to stimulation qualititatively varies for different dynamical regimes.

### Working point 1: Pre-global coherence peak

We begin with the working point WP1 located at *C* = 2.5 and $P_{E}^{\text{base}}-P_{E}^{*}\left(C\right)=0.003$, below peak coherence (Fig 3C, Row 1, Column 2). Here, the system is perched just past the boundary marking the transition between the quiescent state and the commencement of rhythmic dynamics. Hence, regional activity is oscillatory but of relatively low amplitude (see Fig 2C), and the power spectra is broad (see Fig 2D).

#### Local excitations induce distinct modifications to power spectra

**Fig 4:**
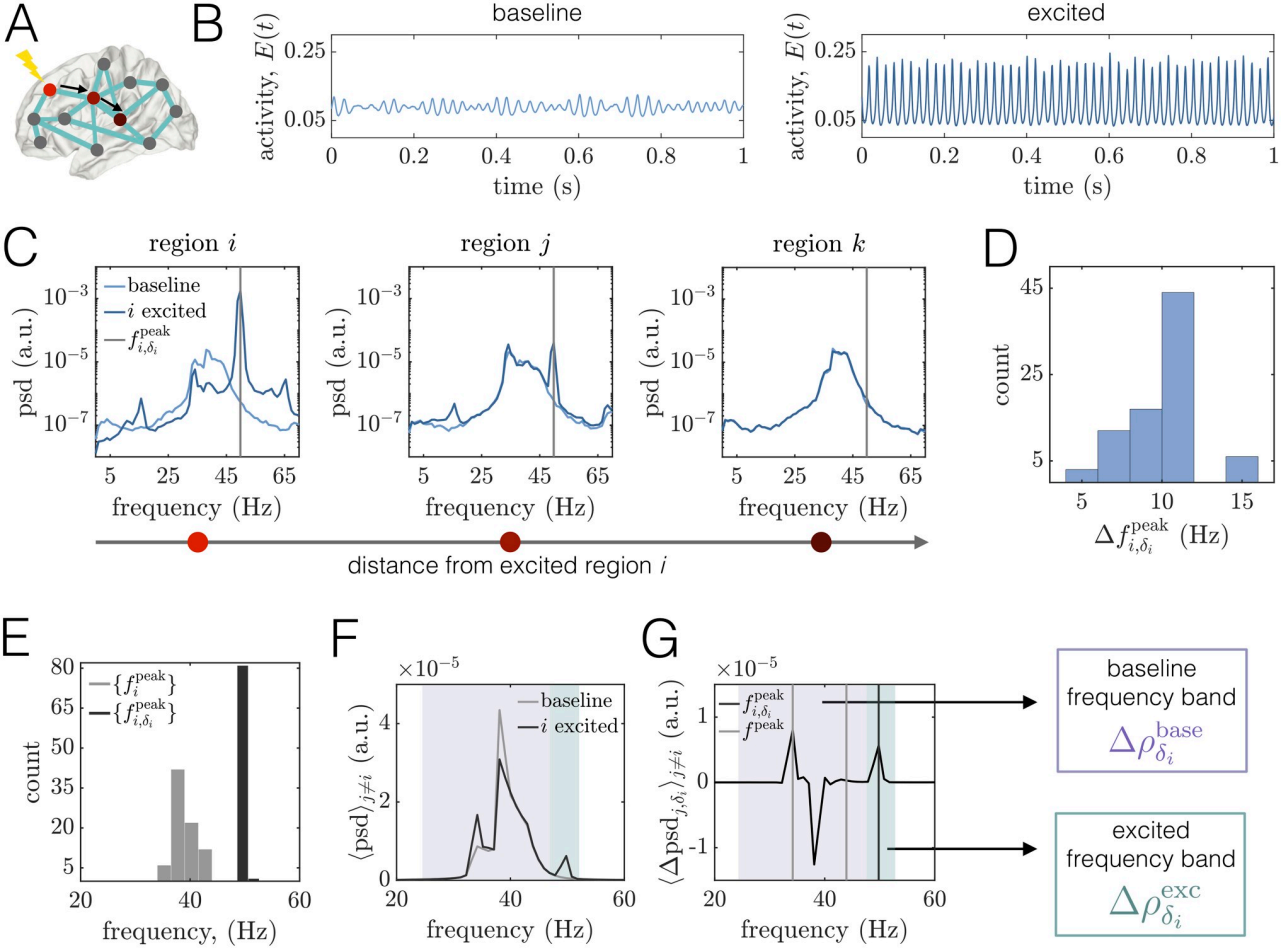
Regional excitation causes local and downstream changes to brain areas’ power spectra in different frequency bands at WP1. *(A)* Schematic of a brain network depicting the stimulated site *i* in brightest red. The black arrows point to two other regions *j* and *k* that lie at progressively further topological distances from the perturbed area in the structural network. In this figure, regions *i*, *j*, and *k* correspond to brain areas 1 (R–Lateral Orbitofrontal), 4 (R–Medial Orbitofrontal), and 10 (R–Precentral), respectively. *(B)* Left: A segment of region *i*’s activity time-course in the baseline condition. Right: A segment of region *i*’s activity time-course when it is stimulated. *(C)* Power spectra of area *i* and two other downstream regions *j* and *k*. In all three panels, the lighter curves correspond to the baseline condition, and the darker curves correspond to the state in which *i* is driven with additional input. The gray vertical lines indicate the peak frequency $f_{i,\delta_{i}}^{\text{peak}}$ of region *i* in the excited condition. *(D)* Histogram of the shift in peak frequency $\Delta f_{i,\delta_{i}}^{\text{peak}}$ induced by stimulating unit *i*, plotted over all choices of the perturbed area. *(E)* Distribution of peak frequencies of all units in the baseline condition $\left\{f_{i}^{\text{peak}}\right\}$ (light gray) and distribution of the peak frequency units acquire when directly excited $\left\{f_{i,\delta_{i}}^{\text{peak}}\right\}$ (dark gray). *(F)* Average power spectra 〈psd〉_*j*≠*i*_ over all units *j* ≠ *i* at baseline (light gray) and when unit *i* is perturbed with additional input (dark gray). *(G)* Average difference ${\langle \Delta {\text{psd}}_{j,\delta_{i}}\rangle }_{j\neq i}$ of the spectra of unit *j* ≠ *i* when unit *i* is excited and in the baseline condition, where the average is over all units *j* ≠ *i*. For reference, the light gray vertical lines denote the minimum and maximum peak frequency across units in the baseline state, and the dark gray line indicates the peak frequency acquired by the stimulated region *i*. Shaded boxes denote two frequency bands of interest: *(1)* the *baseline* band (purple) consisting of the main oscillation frequencies of brain areas under baseline conditions, and *(2)* the *excited* band (green) centered around the peak frequency that the stimulated region inherits. In subsequent analyses, we assess perturbation-induced changes in the PLV between brain areas in the baseline band, $\Delta \rho_{\delta_{i}}^{\text{base}}$ (purple), and in the excited band $\Delta \rho_{\delta_{i}}^{\text{exc}}$ (green).

We first examine the effects of a regional perturbation on areas’ time series and power spectra (Fig 4A–4C). In agreement with past experimental and modeling studies [16, 89, 101, 102], increased drive to the excitatory pool of region *i* increases the amplitude and frequency of its oscillations (Fig 4C, Left). In particular, stimulation causes an increase in the power, narrowing of the spectra (associated with an increase in periodicity of regional activity), and a shift of the peak frequency from ≈ 40Hz at baseline to ≈ 50Hz when excited. We also note the appearance of modulation sidebands in the excited spectra to the left and right of the peak frequency, which arise due to the modulation of the excited region’s time-series by the lower-frequency input it receives from other areas in the network [92]. This modulation also results in a spectral peak at ≈ 16Hz—which is the difference between the new, excited frequency and the sideband peaks, and is a marker of quasiperiodic amplitude modulation in the time-series. To more carefully quantify the effects of an excitation to region *i*, we consider the shift in the peak frequency of unit *i*, $\Delta f_{i,\delta_{i}}^{\text{peak}}=f_{i,\delta_{i}}^{\text{peak}}-f_{i}^{\text{peak}}$, between its excited and baseline states (Fig 4D). Calculating these differences for all choices of the stimulated brain area, we find that they range from about 6Hz to 16Hz, with an average value of $\langle \Delta f_{i,\delta_{i}}^{\text{peak}}\rangle \approx 10.5$ Hz. These perturbation-induced shifts thus yield excited peak frequencies that are well-separated from the range of peak frequencies in the baseline state (Fig 4E).

We next consider the power spectra of two other units *j* and *k* located at increasing topological distances from the excited region, where a shorter topological distance indicates that two areas are linked by a path of stronger structural connections [98]. (Fig 4C, Middle, Right). We observe that unit *j* maintains its initial frequency content, but also develops new peaks centered at the frequency of the excited region and at the difference of the excited frequency and the baseline peak. In contrast, the spectra of unit *k*—which is more weakly structurally connected to the stimulated site—is relatively unchanged. Hence, depending on the network structure, stimulation of region *i* can also cause alterations to other regions’ spectra. In general, the power modulation of a downstream area’s spectra at the peak frequency of the stimulated site decays with increasing topological distance between the dowstream area and the perturbed region (see Sec. SV in S1 Text). To summarize how the spectra of other brain areas are altered by driving region *i* with additional input, we compare the average power spectral density 〈psd〉_*j*≠*i*_ over all units *j* ≠ *i* at baseline and when unit *i* is stimulated (Fig 4F). At baseline, the network-averaged spectra is relatively broad and contains multiple peaks—a main one at 38Hz and a smaller peak around 34Hz. In addition, a local excitation produces complex and broadband alterations in power, as expected in a scenario of quasiperiodic entrainment between nonlinear oscillators [103]. For this example, we observe the appearance of an entirely new peak at 50Hz, but also an enhancement of the lowest baseline peak and a depression of the highest baseline peak. These changes are perhaps more apparent in Fig 4G, which shows the average difference ${\langle \Delta {\text{psd}}_{j,\delta_{i}}\rangle }_{j\neq i}$ in the spectra of unit *j* ≠ *i* between when unit *i* is excited and the baseline condition, where the average is over all units *j* ≠ *i*. In sum, we see that a regional enhancement of neural activity causes non-local modulations in power both at the frequency of the directly stimulated brain area, as well as at the system’s baseline oscillation frequencies. These analyses suggest that there are two relevant frequency bands to consider for subsequent analysis: *(1)* a relatively broad band containing the main frequencies of brain areas in the baseline state, and *(2)* a band centered around the peak frequency of the excited unit. In what follows, we will denote these two bands as “baseline” and “excited”, and consider changes in phase-locking, $\Delta \rho_{\delta_{i}}^{\text{base}}$ and $\Delta \rho_{\delta_{i}}^{\text{exc}}$, in each band induced by local perturbations.

#### Excitations of regional activity induce or alter interareal phase-locking in excited and baseline frequency bands

We are now prepared to study how focal perturbations alter the coordination of network-wide dynamics. Specifically, we examine changes in interareal phase-locking. We separate our analysis into two frequency bands—baseline and excited—by filtering regional activity in each band, extracting Hilbert phases from the filtered signals, and then calculating the PLV for each pair of regions within each band (see Fig 1E; Materials and methods). Since spectra are relatively broad at baseline, a single baseline frequency band for the network is determined by first finding the set of peak frequencies for each unit *i* in the baseline state, $\left\{f_{i}^{\text{peak}}\right\}$. Next, the lower frequency for the common baseline band is set to $\min\{f_{i}^{\text{peak}}\}-10$ Hz, and the upper frequency is set to $\max\{f_{i}^{\text{peak}}\}+10$ Hz. A region-by-region PLV matrix corresponding to the single baseline band is then computed after identically filtering each unit’s activity in this frequency range. To examine phase-locking between units within the much narrower excited band corresponding to a given stimulated region *i*, we first extracted the peak frequency of region *i* when it is stimulated, $f_{i,\delta_{i}}^{\text{peak}}$. A PLV matrix corresponding to unit *i*’s excited frequency is then computed after filtering each region’s activity in the same frequency band ranging from $f_{i,\delta_{i}}^{\text{peak}}-1.5$ Hz to $f_{i,\delta_{i}}^{\text{peak}}+1.5$ Hz. This range was chosen to contain the majority of the excited band peak, while including as little of the original baseline band as possible. If the peak frequency of the stimulated area was not more than 3.5Hz above the largest baseline peak frequency, then we only examined PLV changes in the baseline frequency band. Our choices are motivated by the following observation: the notion of an excited frequency band is only meaningful when a perturbation introduces a new spectral peak into the system that is separated from the frequencies present in the baseline condition. Also note that, unlike in the baseline band, areas exhibit little power at the excited frequency prior to stimulation; hence, we use changes in excited-band PLV as a measure of how effectively induced activity at the excited frequency spreads in the network.

**Fig 5:**
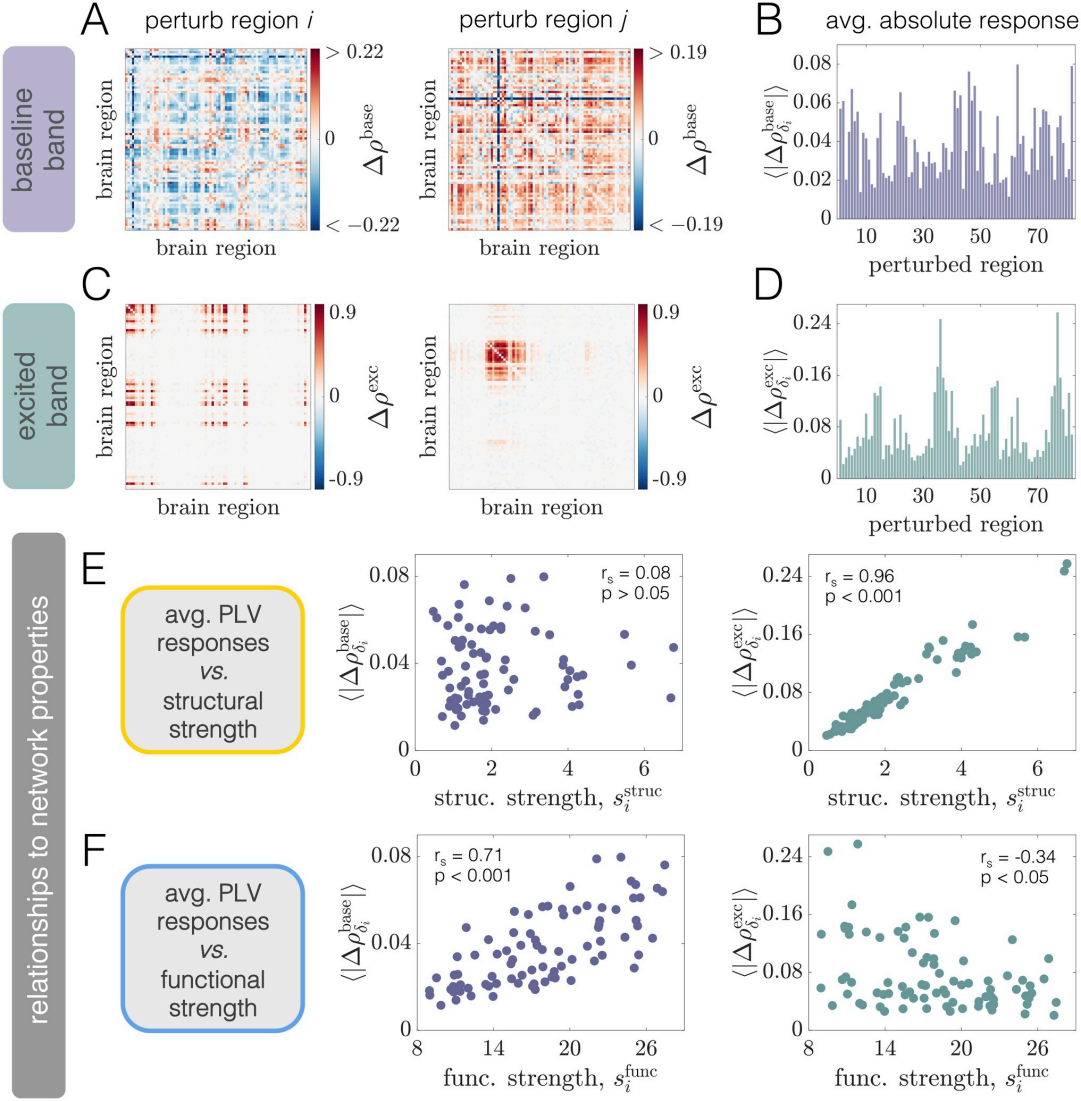
Phase-locking changes at WP1 are driven by local excitations of neural activity, differ between excited and baseline frequency bands, and are differentially related to structural and functional network properties. *(A)* Pairwise changes in the PLV inside the baseline band Δ*ρ*^base^ when region *i* (Left) or region *j* ≠ *i* (Right) is perturbed. In this figure, regions *i* and *j* correspond to regions 4 (R–Medial Orbitofrontal) and 23 (R–Lateral Occipital), respectively. *(B)* Network-averaged absolute PLV changes in the baseline band $\langle |\Delta \rho_{\delta_{i}}^{\text{base}}|\rangle$ caused by stimulation of different brain areas. *(C)* Pairwise changes in the PLV inside the excited band Δ*ρ*^exc^ when region *i* (Left) or region *j* ≠ *i* (Right) is perturbed. *(D)* Network-averaged absolute PLV changes in the excited band $\langle |\Delta \rho_{\delta_{i}}^{\text{exc}}|\rangle$ induced by stimulation of different brain areas. *(E)* The quantity $\langle |\Delta \rho_{\delta_{i}}^{\text{base}}|\rangle$ *vs*. structural node strength $s_{i}^{\text{struc}}$ (Left), and the quantity $\langle |\Delta \rho_{\delta_{i}}^{\text{exc}}|\rangle$ *vs*. structural node strength $s_{i}^{\text{struc}}$ (Right). *(F)* The quantity $\langle |\Delta \rho_{\delta_{i}}^{\text{base}}|\rangle$ *vs*. functional node strength $s_{i}^{\text{func}}$ (Left), and the quantity $\langle |\Delta \rho_{\delta_{i}}^{\text{exc}}|\rangle$ *vs*. functional node strength $s_{i}^{\text{func}}$ (Right). In panels *(E)* and *(F)*, insets indicate Spearman correlation coefficients between the plotted quantities and their associated *p*-values).

To provide intuition about how phase-locking is altered upon a local perturbation, we consider the effect of stimulating two different brain areas (left and right panels in Fig 5A and 5C). These examples show that regional stimulation induces phase-locking at the excited frequency (Fig 5C), but can also cause changes in coherence in the frequency band containing the original oscillatory activity of the system (Fig 5A). Note that the excited band effects are mostly positive (due to the fact that power in the excited band is boosted by stimulation), whereas the baseline band effects can be both positive and negative. Furthermore, the patterns induced in the excited band (Fig 5C) are distinct from the modulations that occur in the baseline band (Fig 5A), and the phase-locking changes are markedly different between perturbation of region *i* (left panels) and perturbation of region *j* ≠ *i* (right panels). Thus, depending on the frequency band considered and the excited area’s location within the large-scale brain network, stimulation induces different responses across the system as a whole.

To summarize the global effect of regional excitation, we calculate the average absolute change in PLV induced by driving each brain area with additional input. We use the notation $\langle |\Delta \rho_{\delta_{i}}^{\text{base}}|\rangle$ and $\langle |\Delta \rho_{\delta_{i}}^{\text{exc}}|\rangle$ to denote the network-average of the absolute PLV changes in the baseline and excited frequency bands, respectively, induced by stimulating region *i*. Note that since the PLV is always between 0 and 1, the maximum possible value of both quantities is 1. Furthermore, we use a phase-randomized null model (described in Sec. SXII of S1 Text) to assess whether pairwise PLV changes are significant. Prior to calculating the network-wide averages $\langle |\Delta \rho_{\delta_{i}}^{\text{base}}|\rangle$ and $\langle |\Delta \rho_{\delta_{i}}^{\text{exc}}|\rangle$, non-signicant changes are set to zero. We observe that the global responses exhibit a large degree of variability across different choices of the stimulated area (Fig 5B and 5D). That is, perturbation of some areas induces larger system-wide modulations of phase-locking than others. Furthermore, regions that induce the largest overall changes in PLV inside the excited frequency band are not necessarily those that cause the largest alterations of PLV in the baseline band. This observation suggests that distinct aspects of the network may be indicative of the overall effects generated at the baseline and excited frequencies.

#### Structural and functional connectivity are linked to different types of phase-locking modulations at WP1

What properties of the system drive or predict the diverse, distributed responses in the baseline and excited frequency bands brought about by focal stimulation? Because the network of anatomical connections couples different brain areas and allows them to directly interact, it is reasonable to hypothesize that the organization of this network should play a role in guiding the influence of a perturbation. To test this hypothesis, we study $\langle |\Delta \rho_{\delta_{i}}^{\text{base}}|\rangle$ and $\langle |\Delta \rho_{\delta_{i}}^{\text{exc}}|\rangle$ as functions of structural node strength $s_{i}^{\text{struc}}$ (Fig 5E). Interestingly, the global PLV modulation induced in the network’s naturally-emergent frequency band is *not* well-predicted by the anatomical strength of the stimulated area (Fig 5E, Left). In contrast, though, we do observe a strong association (Spearman correlation *r*_*s*_ = 0.96, *p* < 0.001) between structural strength and the PLV change elicited in the excited band (Fig 5E, Right). This result indicates that more structurally connected units generate larger overall effects at the enhanced frequency of the directly stimulated area. Because the excited band response is strongly constrained by structure, we also examined whether the effects differed between two broad, anatomically-defined classes of nodes. In particular, we compared the average excited band response for stimulation of cortical *vs*. subcortical areas (both of which are included in the anatomical parcellation). Given this breakdown, we find that the overall effect is significantly higher upon perturbation of subcortical regions (see Fig. E in S1 Text). This result is consistent with the findings of [35, 79], and reflects the notion that subcortical nodes make strong, distributed structural connections that may support large-scale network communication [104].

Although the brain’s structural connectivity plays a crucial role, macroscale activity patterns generally reflect an interplay between connectome architecture and the network’s dynamic regime. Indeed, in Fig 3D we observed that the correlation between structural and functional strength varies in intensity and sign with working point. Importantly, the presence of a functional connection between two brain areas implies an interdependence of their dynamics—enforced by the system’s oscillatory state—that can occur even in the absence of a direct structural connection. Intuitively, we may thus expect the organization of the system’s initial *functional* interactions (which could be non-trivially related to structure), to be indicative of how the coherence pattern is modulated under perturbation. Given this reasoning, we study $\langle |\Delta \rho_{\delta_{i}}^{\text{base}}|\rangle$ *vs*. $s_{i}^{\text{func}}$ (Fig 5F). Relative to structural strength, we observe a strong positive relationship (Spearman correlation *r*_*s*_ = 0.71, *p* < 0.001) between the average absolute change in baseline band coherence and functional strength. Thus, areas that are initially more coherent with other regions in the network tend to yield larger global modulations to baseline band interactions when perturbed. This should be contrasted to the results from structural node strength, for which there was not a strong relationship with absolute coherence changes at the baseline oscillation frequencies. Finally, we consider $\langle |\Delta \rho_{\delta_{i}}^{\text{exc}}|\rangle$ *vs*. $s_{i}^{\text{func}}$ (Fig 5F, Right). Though they are correlated, (*r*_*s*_ = −0.34; *p* < 0.05), the stimulation-induced responses in the excited band are much more strongly predicted by structural rather than functional strength.

The above results emphasize separate consideration of both anatomical network topology and the organization of emergent functional interactions, the latter of which is also driven by the dynamical regime of the system. In particular, for the working point considered here, structural and functional network properties relate to distinct types of perturbation-induced effects. First, phase-locking changes that arise in the excited band reflect the transmission and replication of oscillatory input from the directly excited area to and in downstream regions. If the structural connection between the stimulated site and a downstream area is strong enough, then the drive from the stimulated site will induce a new spectral component in the receiving area (see, e.g., Fig 4C); consequently, the two regions will exhibit phase-locking at the excited frequency. In addition, even two areas that are not directly linked can display a high PLV in the excited band due to strong common input from the stimulated region, or due to the propagation of the stimulated site’s signal along alternative paths in the network. In sum, because spreading of the perturbed area’s activity is highly constrained by the presence of structural connections, regions with stronger anatomical connectivity to other areas more forcefully drive downstream regions and lead to larger excited band effects. Perhaps more interesting are the modulations in coherence that occur in the baseline frequency band. These changes arise not due to a direct transmission of input, but rather via adjustments to the ongoing, mutual entrainment between units’ spontaneous rhythms. For WP1, the resulting alterations to the strength of coherent interactions are more related to the stimulated region’s initial functional connectivity rather than its anatomical strength. Intuitively, this may in part be due to the fact that perturbing a particular area tends to decouple it from other areas at the original oscillation frequencies, such that stimulating regions that are strongly coherent to begin with effectively reconfigures existing functional interactions in the baseline frequency band. Notably, the observed correlation between functional strength and baseline band coherence modulations does not uncover the deeper, precise mechanisms behind the effects. However, because the brain’s collective dynamics can reflect a complex interplay between its oscillatory state and its structural connectivity, the result highlights the importance of considering both aspects when trying to understand network-wide responses to perturbations.

### Working point 3: Post-global coherence peak

Importantly, the collective state of the model can change even when anatomical connectivity is fixed (see Fig 3). To explore how this affects the impacts of regional perturbations on brain network dynamics, we next examine another working point—WP3—located at *C* = 2.5 and *P*_base_ = 0.7. (Note that a working point WP2 between WP1 and WP3 is analyzed carefully in Sec. SIII of S1 Text). In this high background drive state, regional activity is characterized by more regular and higher-amplitude oscillations (see Fig 2G and 2H) relative to both WP1 and WP2. Furthermore, at WP3, the system resides well beyond the point of maximal coherence (Fig 3A), and the baseline PLV matrix is more constrained by anatomical connectivity (Fig 3C, Row 2, Column 4; Fig 3D).

#### Spectral changes are more restrained at the high background drive working point

**Fig 6:**
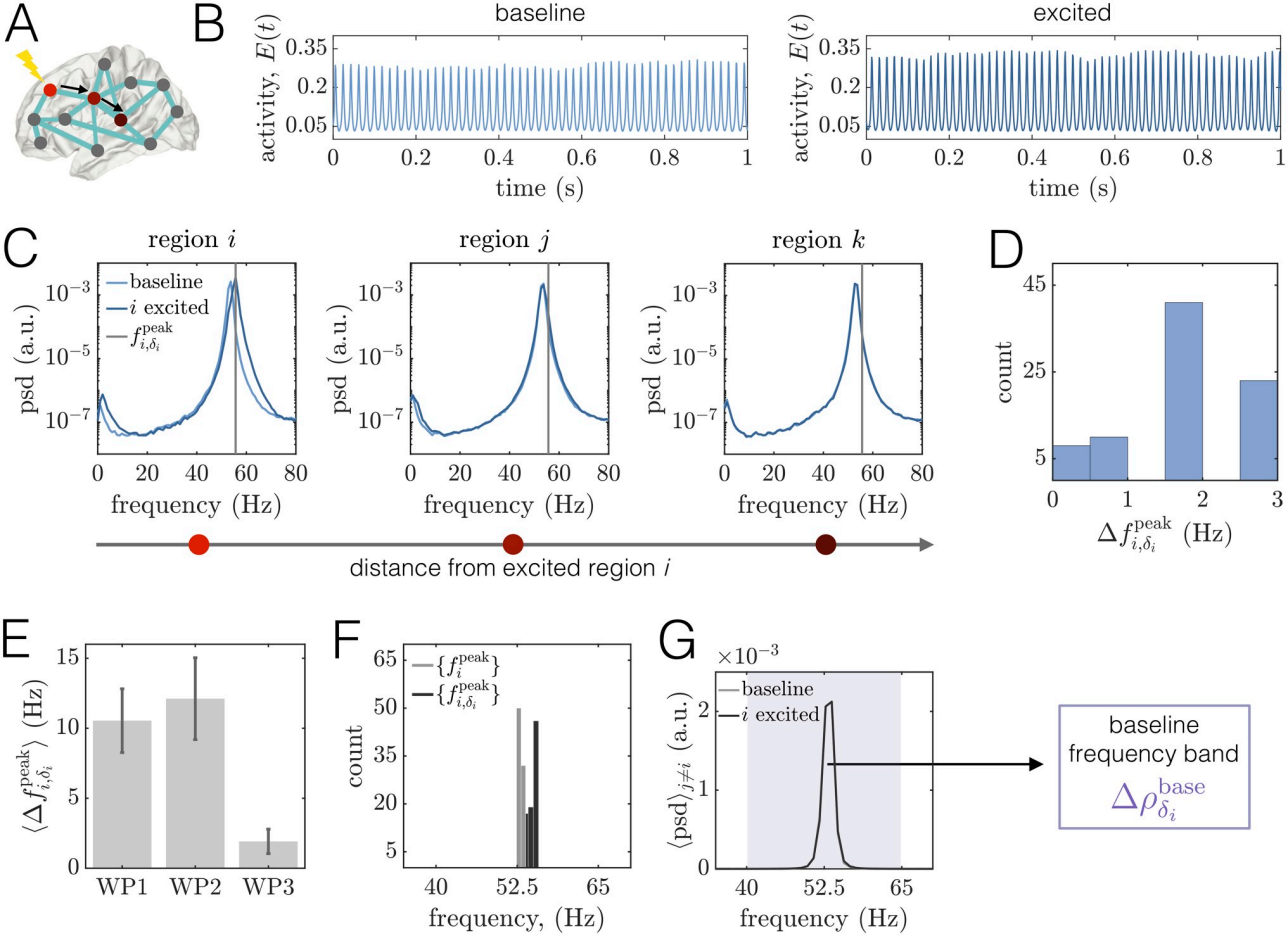
Effects of local excitations on power spectra are more restricted at the high background drive working point (WP3). *(A)* Schematic of a brain network depicting the stimulated site *i* in brightest red. The black arrows point to two other regions *j* and *k* that lie at progressively further topological distances from the perturbed area in the structural network. In this figure, regions *i*, *j*, and *k* correspond to brain areas 1 (R–Lateral Orbitofrontal), 4 (R–Medial Orbitofrontal), and 10 (R–Precentral), respectively. *(B)* Left: A segment of region *i*’s activity time-course in the baseline condition. Right: A segment of region *i*’s activity time-course when it is stimulated. *(C)* Power spectra of area *i* and two other downstream regions *j* and *k*. In all three panels, the lighter curves correspond to the baseline condition, and the darker curves correspond to the state in which *i* is driven with additional input. The gray vertical lines indicate the peak frequency $f_{i,\delta_{i}}^{\text{peak}}$ of region *i* in the excited condition. *(D)* Histogram of the shift in peak frequency $\Delta f_{i,\delta_{i}}^{\text{peak}}$ induced by exciting unit *i*, plotted over all choices of the perturbed area. *(E)* The average shift in the peak frequency of the stimulated region $\langle \Delta f_{i,\delta_{i}}^{\text{peak}}\rangle$ for WP1, WP2, and WP3 (error bars indicate the standard deviation over all choices of the excited unit). *(F)* Distribution of peak frequencies of all units in the baseline condition $\left\{f_{i}^{\text{peak}}\right\}$ (light gray) and distribution of the peak frequency units acquire when directly excited $\left\{f_{i,\delta_{i}}^{\text{peak}}\right\}$ (dark gray). *(G)* Average power spectra 〈psd〉_*j*≠*i*_ over all units *j* ≠ *i* at baseline (light gray) and when unit *i* is perturbed with additional input (dark gray). Because stimulation does not induce a well-separated excited frequency band, we only assess perturbation-induced changes in the PLV between brain areas for a single baseline frequency band (purple area).

Inspection of a region’s activity time-series at baseline and when stimulated with additional input indicates noticeable differences in how perturbations alter local activity at WP3 versus at either WP1 or WP2. Specifically, when the system operates in the high-drive state, a perturbation of the same strength has a much less drastic effect on the stimulated region’s activity, inducing only relatively small changes to its amplitude and frequency (Fig 6B).

Consequences of regions’ enhanced baseline activity for focal stimulation are perhaps more evident from examples of areas’ spectra at baseline and under stimulation (Fig 6C). As for the lower-drive working points, the peak frequency and power of the stimulated region *i* shift to higher values (Fig 6C, Left). However, at WP3, the increase is modest relative to the shifts that occur at either WP1 or WP2, and no modulation sidebands arise in *i*’s spectra under excited conditions. As a result of the more unyielding nature of spontaneous dynamics, stimulation of unit *i* also has relatively little impact on the spectra of downstream regions (Fig 6C, Middle, Right), even if they are positioned topologically close to the perturbed site. To more generally quantify the effects of regional stimulation on areas’ power spectra, we examine the distribution of the shifts in peak frequency $\Delta f_{i,\delta_{i}}^{\text{peak}}$ that occur due to perturbation of each unit. The largest of these shifts is only about 3Hz (Fig 6D). Hence, relative to WP1 and WP2, the average shift in peak frequency $\langle \Delta f_{i,\delta_{i}}^{\text{peak}}\rangle$ is greatly reduced at the high-drive working point (see Fig 6E). Furthermore, unlike the situation in the low-drive state, the distributions of peak frequencies at baseline and under focal stimulation begin to overlap at WP3 (Fig 6F), precluding the notion of separate baseline and excited frequency bands. For this reason, in our subsequent analyses we only consider phase-locking changes inside a single frequency band (Fig 6G). While we refer to this as the “baseline band”, we note that it still contains the peak frequency of the directly excited unit, since its frequency shift is so small.

The results presented in this section indicate that regional dynamics are more robust to perturbations at the high-drive working point. This can in part be understood by considering the effects of the background drive $P_{E}^{\text{base}}$, which is the parameter tuned to move from WP1 → WP2 → WP3. In particular, the increased baseline input level at WP3 means that each unit, if disconnected from the network, would operate closer to the bifurcation separating the quiescent and oscillatory state than would be the case at WP1 or WP2. As a result, stronger oscillations emerge at WP3 when the network coupling is introduced, reflecting the increased influence of recurrent dynamics. The high-amplitude rhythms that arise in the high-drive state are more difficult to disrupt, leading to minimal changes in the power spectra under local perturbations. For the same reasons, it is also more difficult for a local change in activity to propagate and influence the dynamics of remote areas. In contrast, when the system operates at either WP1 or WP2, the baseline oscillations at each brain area are weaker. This lower-amplitude activity is easier to override, yielding the system more plastic and susceptible to local perturbations. This flexibility at WP1 and WP2 is reflected by clear modifications to regional spectra upon local stimulation and the signatures of the stimulation effect in downstream regions (Fig 4 and Fig. C in S1 Text).

#### Focal perturbations yield a distinct and more homogenous set of phase-coherence modulations at the high-drive working point

**Fig 7:**
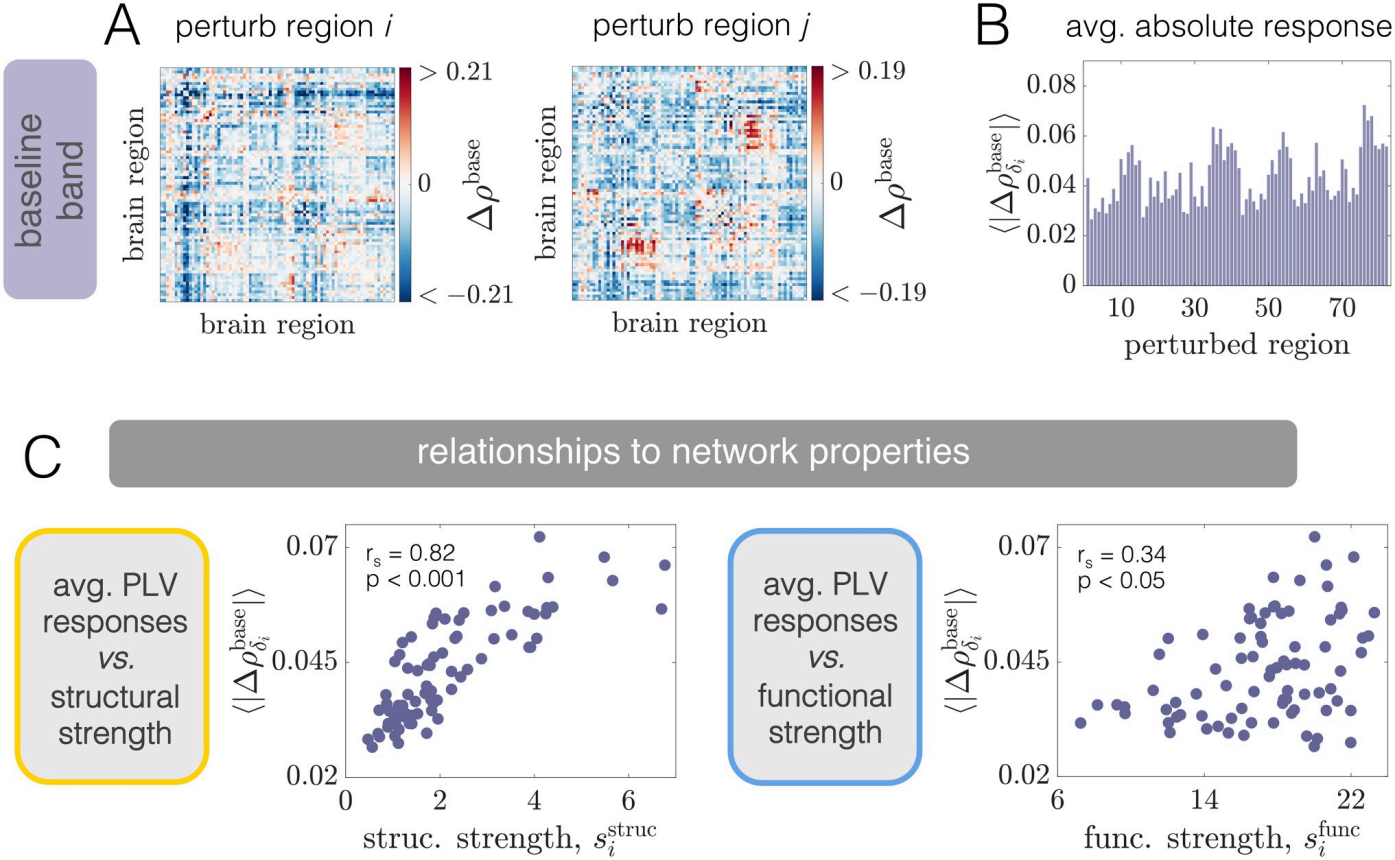
Phase-locking modulations induced by regional stimulation at WP3 and their associations with network properties. *(A)* Pairwise changes in the PLV inside the baseline band Δ*ρ*^base^ when region *i* (Left) or region *j* ≠ *i* (Right) is perturbed. In this figure, regions *i* and *j* correspond to regions 10 (R–Precentral) and 15 (R–Isthmus), respectively. *(B)* Network-averaged absolute PLV changes in the baseline band $\langle |\Delta \rho_{\delta_{i}}^{\text{base}}|\rangle$ induced by stimulation of different brain areas. *(C)* The quantity $\langle |\Delta \rho_{\delta_{i}}^{\text{base}}|\rangle$ *vs*. structural node strength $s_{i}^{\text{struc}}$ (Left) and *vs*. functional node strength $s_{i}^{\text{struc}}$ (Right). Insets indicate Spearman correlation coefficients between the plotted quantities and their associated *p*-values).

While the rigidity of baseline rhythms at WP3 prevents the emergence of a well-defined excited frequency band, we can still assess the effects of selective perturbations on interareal phase-locking in the single, baseline frequency band. We carry out such an analysis in this section, focusing on contrasting the results obtained at WP3 to those obtained previously at WP1 (and WP2). To begin, we show examples of the pairwise changes in PLV induced by stimulation of two different brain areas *i* and *j* (Fig 7A), and the average absolute changes $\langle |\Delta \rho_{\delta_{i}}^{\text{base}}|\rangle$ in phase-locking driven by perturbation of each brain area in the network (Fig 7B). As with WP1, the statistical significance of PLV changes is first assessed with a phase-randomized null model (see Sec. SXII of S1 Text) before computing network averages. Despite differences in the induced average responses across regions, visual comparison of the distribution at WP3 (Fig 7B) and at WP1 (Fig 5B) suggests that there may be less variation across regions when the system operates in the high-drive state. Indeed, although the mean of the average absolute changes across all brain areas is approximately the same at WP1 and WP3, when we compare the coefficient of variation (CoV) of the two distributions of $\{\langle |\Delta \rho_{\delta_{i}}^{\text{base}}\left|\rangle \right\}$, we find that the CoV for WP3 is 0.25, while for WP1 it is 0.45. This indicates that global coherence modulations elicited by regional stimulation are more homogenous across different choices of the stimulated brain area when the system operates in the high background drive regime. Because we are particularly interested in how perturbations may differentially affect network dynamics depending on baseline state, we also investigate if the average absolute PLV modulations are correlated between WP1 and WP3. This relationship is not significant (Fig. G in S1 Text), indicating that the rank-ordering of $\langle |\Delta \rho_{\delta_{i}}^{\text{base}}|\rangle$ is quite different between the low and high drive regimes.

We next considered the associations between structural or functional properties of network nodes and the global phase-locking changes induced by regional stimulation (Fig 7C). At WP3, we find a strong positive correlation between $\langle |\Delta \rho_{\delta_{i}}^{\text{base}}|\rangle$ and structural strength $s_{i}^{\text{struc}}$ (*r*_*s*_ = 0.82, *p* < 0.001; Fig 7C, Left). Thus, larger coherence modulations at the high-drive working point are dependent on the presence of strong anatomical connections emanating from the stimulated region. Note that functional strength $s_{i}^{\text{func}}$ also exhibits a positive correlation with $\langle |\Delta \rho_{\delta_{i}}^{\text{base}}|\rangle$ (*r*_*s*_ = 0.34, *p* < 0.05; Fig 7C, Right), which we might expect given the weak but positive correlation between *s*^struc^ and *s*^func^ at this working point (Fig 3D). However, structural connectivity is undoubtedly a more robust predictor of these effects.

A supplementary analysis (see Fig. H in S1 Text) indicates that the association between $s_{i}^{\text{struc}}$ and $\langle |\Delta \rho_{\delta_{i}}^{\text{base}}|\rangle$ is (largely) explained by a relationship between structural strength and the bulk *decreases* in coherence induced by perturbations. Hence, units with stronger anatomical connectivity to the network as a whole generate larger global breakdowns in coherence when stimulated with constant, additional input. To gain intuition for this result, recall that in the high-drive regime, brain areas exhibit relatively strong and inflexible oscillations at baseline. Furthermore, though not enough to bring about an entirely new excited band, stimulation still slightly accelerates the frequency of the perturbed unit. As a consequence, we may expect stimulation of regions with stronger structural connections—which have more direct influence on other areas—to drive more disruptions in the network’s ongoing dynamics. That is, a given node will tend to be driven out of coherence with other units when stimulated (since it acquires a slightly increased frequency), and high-strength nodes in particular will also be able to decouple other areas from their baseline functional assemblies, in turn causing larger dissociations of functional connectivity.

To conclude this section, we highlight the observation that depending on the oscillatory regime, different aspects of the system best predict global responses to focal stimulation. In the low-drive state, emergent coordination between units activities at baseline is more strongly related to coherence modulations at regions’ spontaneous frequencies, whereas structure is more strongly associated with the effects in the high-drive regime.

### System-wide effects of regional perturbations vary with dynamical state

We have examined distinct operating points characterized by varying regional oscillation strengths, and in turn, different large-scale coordination patterns and relationships to anatomical connectivity. We found that focal perturbations had markedly different effects on brain network dynamics and associations to network connectivity depending on the system’s oscillatory mode. In this section, we more generally examine the effects of perturbations as the nature of regional activity is smoothly varied—by tuning the level of background drive $P_{E}^{\text{base}}$—while holding network structure constant. Throughout this exposition, we set the coupling *C* = 2.5, as before.

**Fig 8:**
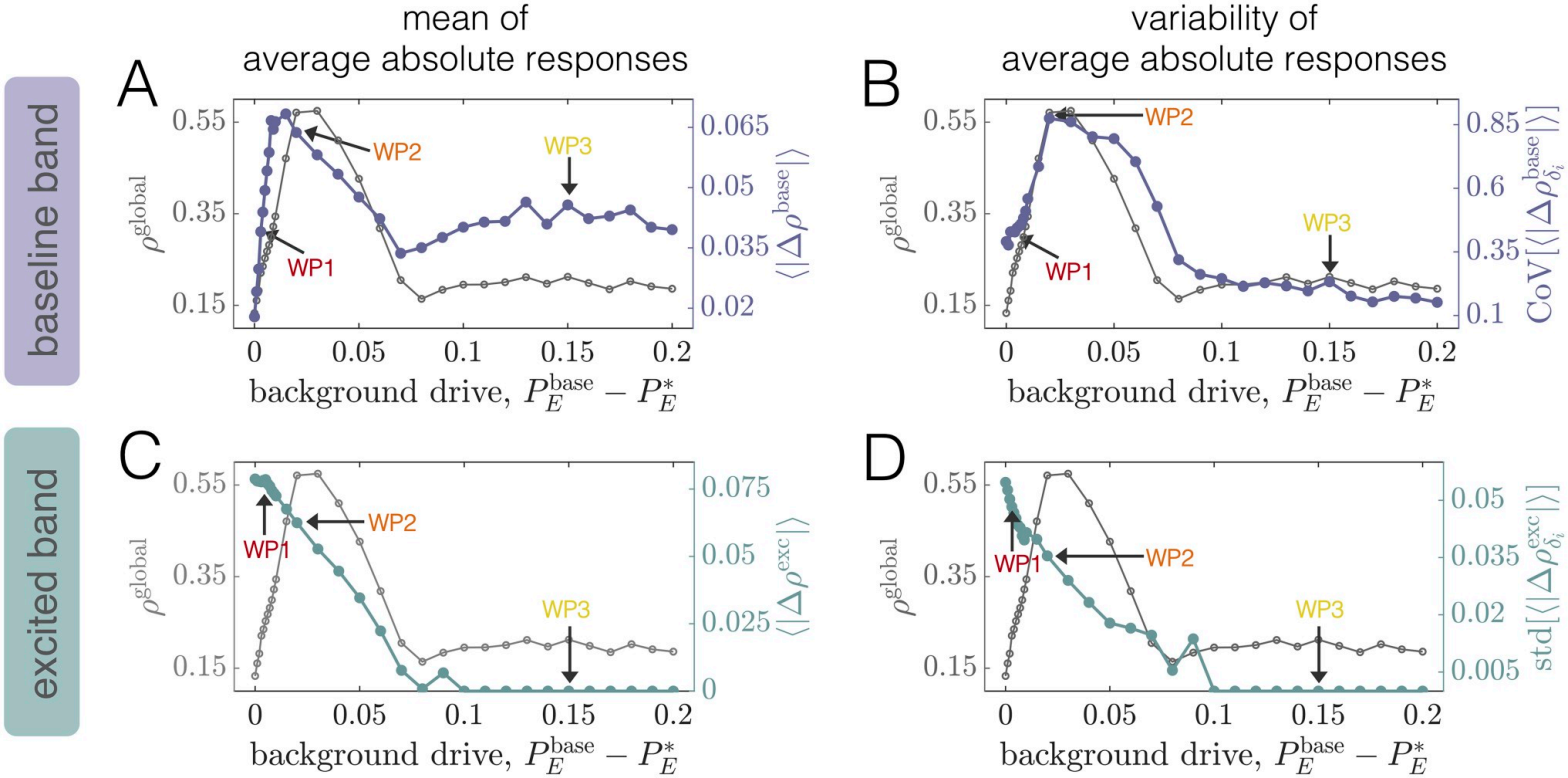
Dependence of global phase-locking changes on the baseline state of the brain network model. *(A)* The left axis (gray) shows the network-averaged baseline PLV *ρ*^global^ as a function of background drive $P_{E}^{\text{base}}-P_{E}^{*}$ for a coupling *C* = 2.5. The right axis (purple) shows the grand average 〈|Δ*ρ*^base^|〉 of the perturbation-induced absolute changes in baseline band PLVs as a function of $P_{E}^{\text{base}}-P_{E}^{*}$. *(B)* The left axis (gray) shows the network-averaged baseline PLV *ρ*^global^ as a function of background drive $P_{E}^{\text{base}}-P_{E}^{*}$ for a coupling *C* = 2.5. The right axis (purple) shows the coefficient of variation of the perturbation-induced average absolute changes in baseline band PLVs, $\text{CoV}[\langle |\Delta \rho_{\delta_{i}}^{\text{base}}\left|\rangle \right]$, as a function of $P_{E}^{\text{base}}-P_{E}^{*}$. *(C)* The left axis (gray) shows the network-averaged baseline PLV *ρ*^global^ as a function of background drive $P_{E}^{\text{base}}-P_{E}^{*}$ for a coupling *C* = 2.5. The right axis (green) shows the grand average 〈|Δ*ρ*^exc^|〉 of the perturbation-induced absolute changes in excited band PLVs as a function of $P_{E}^{\text{base}}-P_{E}^{*}$. *(D)* The left axis (gray) shows the network-averaged baseline PLV *ρ*^global^ as a function of background drive $P_{E}^{\text{base}}-P_{E}^{*}$ for a coupling *C* = 2.5. The right axis (green) shows the standard deviation of the perturbation-induced average absolute changes in excited band PLVs, $\text{std}[\langle |\Delta \rho_{\delta_{i}}^{\text{exc}}\left|\rangle \right]$, as a function of $P_{E}^{\text{base}}-P_{E}^{*}$. (Note that here we consider the standard deviation rather than the coefficient of variation since the mean response in the excited band eventually drops to zero.).

To summarize the response in the baseline band, we first stimulate each region *i* to generate a set of *N* average absolute coherence modulations $\{\langle |\Delta \rho_{\delta_{i}}^{\text{base}}\left|\rangle \right\}$. We then consider the mean of this set across regions, 〈|Δ*ρ*^base^|〉 (which we refer to as the “grand average”), and study its behavior as the background drive is varied (Fig 8A,right axis). We find that the general shape of 〈|Δ*ρ*^base^|〉 as a function of the relative background drive $P_{E}^{\text{base}}-P_{E}^{*}$ tends to mimic that of the global baseline coherence *ρ*^global^ (Fig 8A,left axis), but with a slight shift towards lower $P_{E}^{\text{base}}-P_{E}^{*}$. Importantly, the peak in the PLV modulation curve at intermediate background drive signifies a distinct state at which regional perturbations generate the largest overall changes to coherence in the system’s baseline frequency band. Of note is that this working point occurs just prior to the global PLV peak. Intuitively, the system may exhibit the largest response at this working point because there is heightened potential to both enhance and depress functional interactions when the system is perched at the transition into the maximally ordered state. At lower values of $P_{E}^{\text{base}}-P_{E}^{*}$, stimulation of single brain areas has a smaller average effect, which likely occurs in part because there is less initial coherence for local excitations to disrupt. As *ρ*^global^ begins to decline with increasing $P_{E}^{\text{base}}-P_{E}^{*}$, so too does the overall response to regional perturbation. The grand average of the baseline band PLV modulation approaches a local minimum at $P_{E}^{\text{base}}-P_{E}^{*}=0.07$, after which it settles to intermediate values at high-drive working points well beyond peak *ρ*^global^ (e.g., at WP3).

In addition to the grand average, we also examined the coefficient of variation (CoV) of the distribution of average absolute coherence changes, which we denote as $\text{CoV}[\langle |\Delta \rho_{\delta_{i}}^{\text{base}}\left|\rangle \right]$. In general, $\text{CoV}[\langle |\Delta \rho_{\delta_{i}}^{\text{base}}\left|\rangle \right]$ also exhibits a state dependence (Fig 8B). Specifically, $\text{CoV}[\langle |\Delta \rho_{\delta_{i}}^{\text{base}}\left|\rangle \right]$ begins at an intermediate value for the lowest background drive, increases to a global maximum, and then declines to below its initial value for high-drive states. This behavior indicates that global responses exhibit less variability across different choices of the stimulated site when the system operates in states of strong baseline rhythms. In contrast, the network-wide impacts of regional stimulation display more dispersion in the low-drive regime, with variability peaking near maximum *ρ*^global^ (WP2). For these states, the overall response in the baseline band is thus more dependent on the precise location in the network that is perturbed.

We next assess how the dynamical state influences the global response induced in the excited frequency band. In this case, the grand average 〈|Δ*ρ*^exc^|〉 of the absolute excited-band PLV changes steadily decays and eventually vanishes with increasing $P_{E}^{\text{base}}-P_{E}^{*}$ (Fig 8C), as does the variability of the response distribution (Fig 8D). This behavior is due to the amplification of baseline oscillations with increasing background drive: as regions’ spontaneous dynamics become more difficult for perturbations to override, the emergence of widespread phase-locking at regions’ excited frequencies declines. Eventually, stimulation no longer yields large enough frequency shifts to induce an excited band at all. This result thus indicates that activity from the stimulated area is most effectively propagated to downstream regions when the network operates in a state of weak baseline activity.

**Fig 9:**
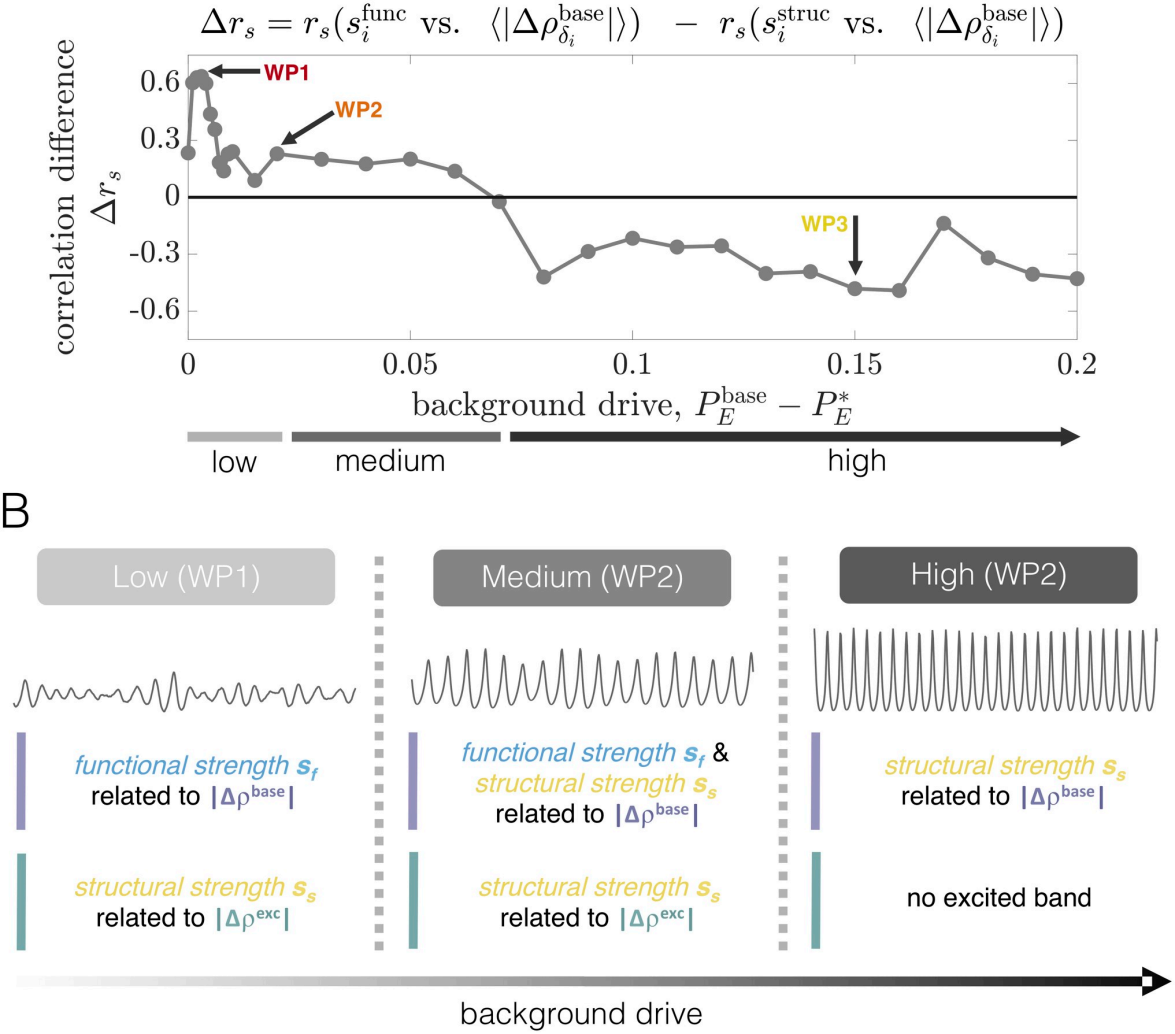
Relationships between phase-locking modulations and the structural or functional connectivity of the stimulated site vary with working point. *(A)* Difference Δ*r*_*s*_ in the strength of the correlation between the average absolute baseline band PLV changes $\langle |\Delta \rho_{\delta_{i}}^{\text{base}}|\rangle$ and structural ($s_{i}^{\text{struc}}$) or functional ($s_{i}^{\text{func}}$) node strength, plotted as a function of the baseline drive $P_{E}^{\text{base}}-P_{E}^{*}$ for a coupling *C* = 2.5. The difference is defined such that when the curve is positive, overall coherence modulations exhibit a stronger correlation with functional rather than structural strength. The arrows mark the locations of the different working points studied in detail in the main or SI Text. *(B)* A schematic summarizing how structural *s*^struc^ or functional *s*^func^ strength are related to either baseline or excited band PLV changes for different dynamical regimes. As the background drive varies from low (WP1) to medium (WP2) to high (WP3), the oscillatory state of the system changes, and so does the association of different phase-locking modulations to structural or functional network properties.

For our final analysis, we examine more generally how the relationships between stimulation-induced PLV modulations and structural or functional network connectivity depend on the system’s operating point. In particular, we consider the correlations between the average absolute baseline band coherence changes $\langle |\Delta \rho_{\delta_{i}}^{\text{base}}|\rangle$ and the structural $s_{i}^{\text{struc}}$ or functional $s_{i}^{\text{func}}$ strength of the perturbed unit. To highlight the differing levels of association between the global responses and either structural or functional connectivity, Fig 9A shows how the difference Δ*r*_*s*_ in the strength of these correlations varies with background drive $P_{E}^{\text{base}}-P_{E}^{*}$ (individual correlations are shown in Fig. J of S1 Text). We observe that the relationships between phase-coherence modulations and structural or functional strength can depend strongly on the system’s baseline state. For the bulk of the “low”-drive regime (working points below peak global coherence), there is a stronger relationship between $\langle |\Delta \rho_{\delta_{i}}^{\text{base}}|\rangle$ and $s_{i}^{\text{func}}$ (rather than $s_{i}^{\text{struc}}$). This enhanced association between a region’s initial functional connectivity and the overall response it induces upon stimulation is largest at dynamical states near WP1, where there is an intermediate level of coherence in the network at baseline. In the “medium”-drive regime, stretching from just prior to peak global coherence up to the beginning of the plateau region in Fig 3A, the correlation between $s_{i}^{\text{struc}}$ and $\langle |\Delta \rho_{\delta_{i}}^{\text{base}}|\rangle$ starts to increase, but functional strength continues to remain more strongly associated with the global modulations elicited in the baseline band. Finally, we observe a clear “switch” at $P_{E}^{\text{base}}-P_{E}^{*}\approx 0.07$, after which structural connectivity becomes relatively better at predicting the overall response to perturbation than functional connectivity (note that this transition occurs in tandem with baseline functional and structural strength becoming positively correlated; see Fig 3D). This behavior marks the onset of the “high”-drive regime, and persists across the remainder of the background drives examined.

To conclude this section, we schematically summarize in Fig 9B the most robust associations between either structural or functional connectivity and the PLV modulations induced by focal perturbations in either the baseline or excited frequency bands. Although we have not comprehensively detailed the effects of perturbations for all possible working points of the model, the results discussed throughout the text highlight the critical influence of a brain network’s collective dynamical state in dictating the outcomes of localized stimulation.

## Discussion

In this study, we set out to explore relations between large-scale brain connectivity, dynamics, and the local and widespread impacts of regional perturbations to neural activity. Following the efforts of past work [35], we built a reduced computational model wherein brain areas were represented as Wilson-Cowan neural masses with long-range coupling between regions constrained by empirical diffusion tractography measurements. We then investigated how stimulation of a particular brain area affected network dynamics, and asked whether and how the collective dynamical state of the system plays a role in modulating such effects. Here, we chose to examine state-dependence by changing the combination of generic background drive and interareal coupling strength, which vary the extent to which local *vs*. network interactions drive regional dynamics, respectively. By tuning these parameters, we identified qualitatively distinct dynamical regimes of the model, and at these different working points, we assessed how local excitations of fixed strength induced or modulated interareal phase-locking. We found that, depending on the baseline regime of the system, the network exhibited different responses to regional perturbations. Furthermore, altering the working point of the model also qualitatively altered the relationships between stimulation-induced effects and properties of structural and functional network connectivity. To the best of our knowledge, these points have yet to be investigated at the whole-brain scale via computational modeling.

Before moving on to a further discussion of this study’s results and limitations, it is critical to state that it is not the first to examine the impacts of perturbations using computational models of brain dynamics. In fact, our work was motivated and inspired by a number of previous large-scale modeling studies that have uncovered key insights into either the network-wide [35–38] or state-dependent [48–50] influences of stimulation. Because our study builds on its methodology, we specifically emphasize the investigation in [35], where (as here) the authors used interconnected Wilson-Cowan units to simulate brain activity, and then utilized recent advances in network control theory [105] to make predictions about the overall functional effects of regional stimulation. We also bring particular attention to the study conducted in [48], where the authors built a simplified model to examine the state-dependent effects of alternating current stimulation on local cortical oscillations. As part of their study, they found that the strength of endogenous oscillations altered the susceptibility of local dynamics to external stimulation. In the present investigation, we vary the background drive (the main tuning parameter in the WC model), which also modulates the amplitude of regional activity. Therefore, although our study is focused on the network-wide effects of focal perturbations, it is necessary to heed and connect our results to those previously reported in [48].

While our work builds upon these and other past efforts, it is important to highlight some key distinctions and extensions of our analysis. First, in contrast to [36], we opted to use the more complex—but in some ways more biophysically-motivated—Wilson-Cowan system as the fundamental dynamical unit in the model, rather than a pure phase oscillator. Studying models that incorporate both phase and amplitude dynamics will likely be critical for a more complete understanding of oscillatory neural activity, since variations in amplitude can affect functional couplings in the brain, are modulated by behavioral conditions, and are also elicited by various forms of stimulation [68, 102, 106–108]. A second key difference between our study and those conducted in [35] and [37] is that the aforementioned studies analyzed a situation in which brain activity was assumed to be in a quiescent state prior to stimulation. Here, however, we were interested particularly in states corresponding to ongoing oscillatory activity, and the interaction between those baseline rhythms and increased excitation to a particular brain area. Finally, in an extension of prior work that has begun to examine the state-dependent impacts of stimulation on single cortical areas [48], we wished to consider a large-scale network model that allowed for an analysis of how stimulation can disseminate to induce or modulate dynamical interactions between widespread brain areas. Hence, the key contribution of this study is its simultaneous investigation of *(1)* not only the focal, but also the *distributed* impacts of regional stimulation, and *(2)* how the *collective* regime of system activity (i.e. that arising from a combination of local dynamical properties and network coupling) influences such network-wide effects. To the best of our knowledge, it remains an open question how the collective state of brain activity alters the way focal stimulation impacts functional relations between distant brain areas.

In the rate model implemented here, the general focal effect of stimulation was to increase the amplitude and frequency of activity in the perturbed area. Such changes in neural activity are broadly consistent with the effects of natural stimulation of certain cortical areas, such as visual stimuli impinging on visual cortex [16, 102] and the effects of increased excitatory drive to neuronal populations generally [101]. However, we also observed that an excitation of fixed strength had significantly stronger effects on local activity (i.e., the induced shift in power and frequency was larger) for states of lower background drive (e.g., at WP1 and WP2). Thus, for these working points, perturbations led to the emergence of a new, “excited” frequency in the system that was well-separated from the main oscillation frequencies at baseline. In contrast, the enhanced regional activity present in the high-drive regime (WP3) was significantly less responsive to perturbations (in that stimulation did not generate a well-separated excited frequency). Though the analogy is not perfect, this behavior is undoubtedly akin to and consistent with the findings reported in [48], wherein alternating current stimulation induced the strongest effects at the stimulation frequency in the absence of strong endogenous oscillations.

Beyond local responses, it is also critical to acknowledge that a given neuronal population exists in the context of a larger network of areas, such that local changes in activity can induce distributed effects [11–14]. Indeed, we found that in states of low background drive, areas topologically close to the stimulation site also developed spectral peaks at the frequency of the stimulated region. These downstream signatures—caused by strong oscillatory drive from the directly excited area—are thus qualitatively similar to those induced by rhythmic stimulation, where activity in the targeted site shows enhanced power at the stimulation frequency [48, 109]. In general, the network’s global response in the excited frequency band was tightly constrained by anatomical connectivity, demonstrating that these effects arise due to a direct propagation of the stimulated area’s strong rhythmic activity along structural pathways. Interestingly, we found that focal perturbations could also modify downstream regions’ spectra at frequencies other than the peak frequency of the directly excited area. Such generic broad-band alterations of spectra can occur in coupled neuronal populations with long-range excitatory interactions [110]. Indeed, in one of only a few possible scenarios, interacting nonlinear oscillators engage first into quasiperiodic dynamics, and then develop chaos, which is associated with spectral changes over continuous ranges [103]. We additionally saw that local excitations could alter levels of temporal coordination between brain areas’ activity in a frequency band containing the system’s spontaneous, “baseline” oscillations. Furthermore, in the low-drive regime, we observed that global absolute modulations in baseline-band coherence were actually more strongly related to the stimulated region’s functional strength than structural strength. This is in contrast to the excited-band responses, which were clearly mediated by anatomical connectivity. Importantly, changes in the baseline band arise from modifications of the dynamic interactions between brain regions’ ongoing rhythms, which can depend on a more complex and intricate interplay between both network structure and the collective oscillatory state of the system. Finally, in the high-drive regime, system-wide changes in phase-locking became more homogenous across different choices of the excited brain area, and were better predicted by the stimulated region’s structural strength. This reflects the idea that in a state of robust regional oscillations, focal stimulation will only yield a large global effect if the perturbed site has strong anatomical connectivity.

Although the field has not reached a clear consensus on the distributed effects of stimulation, the general finding in our study that increased drive to a single brain area can spread and reorganize functional interactions is consistent with previous modeling work [35, 37]. We also make some new, specific predictions about different types of changes that can occur and investigate potential mediators of these effects. Perhaps most interesting is the suggestion that both network structure and coordinated dynamical organization may play a role in guiding the non-local effects of perturbations. While network neuroscience has traditionally focused on how structure can predict function [74, 111–114], it is critical to acknowledge that the behavior of neural systems need not be completely constrained by structure alone [40, 88, 99, 115]. In particular, by modulating intrinsic properties of neural units, a single structural connectome can generate different patterns of coordinated activity [100, 116, 117]. Indeed, we observed that although structural connectivity may partially constrain the organization of functional interactions, the way and the extent to which it does so is dependent on the nature of regional dynamics. Our findings indicate that, as a consequence, the collective oscillatory state of the system may also be important in determining how a perturbation can lead to distributed changes in functional interactions. Specifically, we found that in certain cases, functional connectivity was actually a better predictor of the overall response to perturbations than structural connectivity. Although the presence of an association with functional strength should not be misinterpreted as a mechanistic explanation for how stimulation alters dynamics, such an observation can provide insight, especially when contrasted with the role of structure alone. In total, our results indicate that depending on the collective state of network activity, the widespread impacts of focal perturbations can differ, and may be driven by distinct processes.

Though we examined a simplified model, our results may be relevant to other work and a growing literature on brain state-dependent stimulation [43–50], which recognizes that the effects of exogenous perturbations can be conditional on the endogenous rhythmic or spontaneous activity of the system at the time of stimulation. For example, empirical studies have shown that outcomes of stimulation can differ as a function of cognitive state (i.e., task *vs*. rest) [44–47, 118]. Importantly, a few computational studies have investigated and provided explanations for such state-dependent responses to alternating current stimulation [48–50], though have only considered models of a single or a few coupled cortical and/or subcortical regions and have thus focused on stimulation’s local effects. Our goal here was to begin filling the need for larger-scale models that systematically consider the influence of whole-brain anatomical connectivity in conjunction with the collective dynamics of brain activity in shaping the widespread effects of stimulation. Importantly, stimulation also holds promise as a technique for moving the brain between specific, desired states [41, 42, 105]. The ability to control brain network dynamics has important applications in the treatment of neurological diseases such as Parkinson’s and epilepsy [32–34], in which neural activity goes awry. As they are refined, computational studies that put focus on how stimulation interacts with internal, ongoing dynamics in large-scale brain networks have the potential to inform future control and stimulation-based experiments and therapies [119, 120].

There are also a number of methodological considerations to comment on regarding this work. First, we used a relatively coarse-grained parcellation (*N* = 82 regions) to construct human structural brain networks. This resolution is roughly consistent with several other whole-brain modeling studies [35, 66–69], and is tractable for computationally intensive simulations. However, the employed parcellation is a simplification of the underlying anatomy, since the regions represent large pieces of neural tissue and hence remain agnostic to potentially important structural heterogeneities at finer scales. Moreover, we used Euclidean distances between region centers to approximate structural connection lengths [66, 69], but one could derive perhaps more accurate estimates based on fiber trajectories [82]. It is also important to note that the limitations of human brain imaging and tractography preclude a perfect reconstruction of interareal connections [121]. One primary drawback is that these methods cannot resolve the directedness of interareal connections, which could impact subsequent results [70]. Finally, we used a group-representative connectome in this study. On the one hand, this allowed us to focus on general trends and behaviors, but on the other, it leaves no room for examining how individual differences may affect certain findings. In future work, it will be interesting to explore how various results generalize to both higher-resolution and higher-quality brain data, and to understand how variability in brain structure across different human subjects [78, 122, 123] or even across different species [124] may relate to differences in how perturbations of neural activity are expressed in system dynamics.

Another limitation of this study concerns the model used to simulate brain activity. We opted to use the canonical Wilson-Cowan model [53]—which embodies a tradeoff between biological realism and tractability—but future work could examine other choices for the node dynamics. Futhermore, while the WC model constitutes an arguably realistic improvement over simpler linear models, phase-oscillators, or generic Hopf bifurcation models, it is still phenomenological in nature and our implementation of the model is highly idealized. For example, the simulated oscillations in this work tend to be much more regular than physiological rhythms, which limits the model’s biological plausibility. Similarly, the model yields unrealistically high levels of sustained, network-wide coherence. If observed in brain network dynamics, such states would be much more likely to occur in a transient manner as the brain transitions between segregated and integrated states dynamically [96, 125–127]. Though building whole-brain mean-field models that yield more realistic, transient and time-varying switches between synchronized or desychronized states is difficult, this is an exciting direction for future study.

In general, there are several ways the model could be improved upon to increase its biological validity. For instance, for simplicity and in line with past work [35, 66, 74–77, 81], we only considered long-range couplings between excitatory populations. However, interareal connections likely target both excitatory and inhibitory neurons, and it would therefore be relevant to examine the effects of long-range excitatory-to-inhibitory coupling. We also used a fixed value for the signal propagation velocity. Although we attempted to choose an empirically constrained value, it is known that delays can have significant consequences on brain dynamics [73, 128], and this could be explored further. Moreover, in the WC model, oscillations are generated via the interaction of excitatory and inhibitory neuronal populations, which is considered a biophysically-plausible mechanism underlying rhythmicity in the gamma and beta bands [4, 129]. However, lower frequency oscillations (e.g. alpha band activity), may be generated—at least in part—by thalamocortical loops [130]. Importantly, past models of small thalamocortical circuits (i.e. with only a few coupled regions) have shown that these interactions are important for explaining empirical results regarding the state-dependent responses of cortical alpha oscillations to AC stimulation [48]. Along these lines, an interesting direction for future work would thus be to incorporate more realistic dynamics of subcortical regions and biologically-motivated thalamocortical couplings [131]. Another assumption of the model is that each unit has identical parameters. While this is a reasonable and useful setup to analyze first, there is an important body of literature detailing specific heterogeneities across brain areas. For example, different regions may operate at different intrinsic time-scales and have different intrapopulation architectures (e.g., excitatory or inhibitory coupling strengths) that lead to functional specialization [132]. Recent modeling efforts have begun to incorporate some of these additional complexities, finding that doing so can lead to more realistic baseline dynamics and can explain certain empirically-observed behaviors not accounted for by simpler models [84]. The model implemented here also assumes oscillations in a single gamma frequency range. However, brain areas can exhibit rhythmic activity in multiple frequency bands, which potentially arise from different cortical layers [133]. In future work, it will be important to ask how the impacts of perturbations depend on collective state when additional details about the heterogeneity of the underlying anatomy or dynamics of brain areas are included in the model [36, 89]. Indeed, these steps will allow one to make more concrete and biologically-meaningful statements about how different regions influence large-scale activity patterns. That said, we also stress that the model considered in the present work does not prevent the ensuing dynamics from being complex and rich, and actually allows us to appreciate how dynamical—in addition to structural—complexity can be a key driver of stimulation-induced effects.

With respect to our analysis, it is also important to state that we examined only a few representative working points of the model, out of the many that exist. Though our goal in this work was to illustrate that the collective state of the system can influence the effects of local perturbations, we do not claim to have provided an exhaustive description of all possible behaviors. Furthermore, our analyses of the interareal coherence modulations induced by focal perturbations examined network-averaged responses and absolute changes. In other words, we focused on characterizing aggregate reorganizations in functional couplings. This focus leaves at least two clear and important directions for subsequent work. First, one could try to further disambiguate what leads to strengthening *vs*. weakening of interareal coherence upon focal perturbation. A deeper understanding of when each outcome occurs would likely require significantly more complex analyses of not only the structural and dynamical properties of the stimulated node, but also of other areas. Second, one could attempt to discern how focal perturbations alter dynamic interactions on finer scales, for example, between individual pairs of regions. Again, this would almost certainly require investigating which aspects of non-stimulated areas at baseline allow them to become engaged or disengaged when another region is perturbed. The complexity of both network structure and dynamics make these tasks challenging, but obtaining a more refined understanding of the underlying mechanisms is essential for making useful comparisons against empirical studies.

Perhaps the biggest limitation of the model presented here is that its baseline dynamics were not tuned according to empirical measurements of large-scale brain activity. To address this in future work, model parameters could be fit separately for different empirically-observed brain states [134], such that various aspects of simulated whole-brain activity match the experimental activity patterns in each case. Then, the effects of stimulation could be re-investigated in the context of the empirically-validated large-scale model to make more biologically-informed predictions. Because the current implementation lacks these constraints, it should be interpreted as a canonical, reduced example.

Another interesting direction for future work would be to systematically compare the impacts of perturbations when implemented on empirical brain network architecture versus other canonical network models. This may allow one to test which effects generalize across different network topologies and which are specific to brain network organization. For example, the recent study in [135] examines how human cortical networks may be structured to support ignition dynamics relative to other canonical network models, finding that the well-connected structural core present in actual brain networks plays a critical role. In extending this type of analysis, one could perhaps also consider whether the brain is especially well-organized for oscillatory control via localized perturbations, and how the brain’s controllability depends on its current state. In other words, does the brain exhibit special topological or dynamical features that enhance the ability of focal stimulation to push it into particular collective, oscillatory modes? Importantly, the controllability of large-scale brain networks is often studied using tools from linear network control theory [105]; however, understanding the control of oscillatory activity in the brain will require extending this framework to incorporate non-linear behaviors [136].

In addition to investigating alternative and/or more detailed models, forthcoming studies could also consider different ways of operationalizing perturbations to neural activity. As a first step, here we implemented “stimulation” as a constant increase in excitatory drive [35]. Given the already complex nature of network dynamics in the absence of stimulation, starting with simple, constant perturbations is critical for building intutions. However, it will be important for future whole-brain modeling studies to implement more realistic and experimentally viable forms of perturbations, which are often time-varying and transient in nature. One clear choice would be to model alternating current stimulation, for example. In this case, the stimulated area receives oscillatory input of a particular amplitude and frequency [137], both of which can be independently tuned. While this kind of stimulation has been examined in smaller thalamocortical circuits [48–50], it would be exciting to scale up to systems-level models. One could also investigate alternative ways of modulating brain state. In this study, we varied the baseline dynamical regime in perhaps the most straightforward way possible, by tuning the level of excitatory input globally for all network elements. This variation changed the local dynamics of each brain area, and in turn, the macroscopic state of the system as a whole. However, brain state could also be modulated by tuning a different physiologically-interpretable parameter, such as the gain in the sigmoidal activation functions. Indeed, recent modeling studies have shown that altering neural gain can lead to dynamical regimes in which functional integration and segregation are balanced [138]. It would also be interesting to understand how widespread changes of neural gain affect the way focal perturbations materialize.

In sum, we conducted an idealized investigation into the effects of focal stimulation on brain network dynamics, focusing on how the system’s collective state influences the distributed impacts of such perturbations. To the best of our knowledge, this latter point has only recently begun to be examined in the context of large-scale brain networks, and therefore warrants investigation via simplifed models. However, the results of this study must be interpreted cautiously as they are yet to be validated by data. Comparing conjectures based on reduced models against empirical findings is necessary to substantiate whether or not the model provides biologically meaningful insight. One testable prediction from the model presented here is that focal stimulation propagates to cause downstream modulations of power and phase-locking at both the dominant frequency acquired by the activated area, but also at regions’ baseline frequencies. Investigating this would require an experiment in which stimulation forces the perturbed area to oscillate at a well-defined frequency—which could perhaps be achieved by alternating current stimulation [7]—and a simultaneous measurement of other brain areas’ dynamics. Moreover, testing variations in the global effects of perturbing different regions would require multiple brain areas to be stimulated and responses from distributed regions simultaneously recorded. Recent advances combining non-invasive brain stimulation with measurement modalities like EEG and MEG [26–30] are making this increasingly possible. These types of experiments [26–30] will be crucial in validating results from computational models that posit how stimulation alters functional couplings across different parts of the brain. Furthermore, one could test how widespread changes of power and phase-locking depend on baseline brain activity, for example, during resting vs. task conditions. Notably, some experimental studies have indeed begun to test how brain state affects the local outcomes of stimulation [43–47], which has also been examined in biophysical models [48–50]. In order to use whole-brain simulation studies to better understand how the dynamic regime of the brain as a whole mediates the network-wide effects of stimulation, it will be necessary to inform these models with measurements of large-scale brain activity. Although here we implemented a more abstract model, working towards increasingly realistic and experimentally testable models is an exciting direction for forthcoming studies.

## Supporting information

S1 Text This supplementary file contains additional analyses and figures, further descriptions of methods, a table denoting brain region labels, and a citation diversity statement. (PDF)

## References

[1] Buzsaki G. Rhythms of the Brain. Oxford University Press; 2011.

[2] Varela F, Lachaux JP, Rodriguez E, Martinerie J. The brainweb: Phase synchronization and large-scale integration. Nature Reviews Neuroscience. 2001;2(4):229–239. 10.1038/35067550 11283746

[3] Ward LM. Synchronous neural oscillations and cognitive processes. Trends in Cognitive Sciences. 2003;7(12):553–559. 10.1016/j.tics.2003.10.012 14643372

[4] Wang XJ. Neurophysiological and computational principles of cortical rhythms in cognition. Physiological reviews. 2010;90(3):1195–1268. 10.1152/physrev.00035.2008 20664082 PMC2923921

[5] Salinas E, Sejnowski TJ. Correlated neuronal activity and the flow of neural information. Nature Reviews Neuroscience. 2001;2(8):539–550. 10.1038/35086012 11483997 PMC2868968

[6] Fries P. Neuronal Gamma-Band Synchronization as a Fundamental Process in Cortical Computation. Annual Review of Neuroscience. 2009;32(1):209–224. 10.1146/annurev.neuro.051508.135603 19400723

[7] Thut G, Miniussi C, Gross J. The Functional Importance of Rhythmic Activity in the Brain. Current Biology. 2012;22(16):R658–R663. 10.1016/j.cub.2012.06.061 22917517

[8] Kopell N, Kramer M, Malerba P, Whittington M. Are Different Rhythms Good for Different Functions? Frontiers in Human Neuroscience. 2010;4:187 10.3389/fnhum.2010.00187 21103019 PMC2987659

[9] Cannon J, McCarthy MM, Lee S, Lee J, Börgers C, Whittington MA, et al Neurosystems: brain rhythms and cognitive processing. European Journal of Neuroscience. 2014;39(5):705–719. 10.1111/ejn.12453 24329933 PMC4916881

[10] Fries P. Rhythms for Cognition: Communication through Coherence. Neuron. 2015;88(1):220—235. 10.1016/j.neuron.2015.09.034 26447583 PMC4605134

[11] Shafi MM, Westover MB, Fox MD, Pascual-Leone A. Exploration and modulation of brain network interactions with noninvasive brain stimulation in combination with neuroimaging. The European journal of neuroscience. 2012;35(6):805–825. 10.1111/j.1460-9568.2012.08035.x 22429242 PMC3313459

[12] Polania R, Nitsche MA, Ruff CC. Studying and modifying brain function with non-invasive brain stimulation. Nature Neuroscience. 2018;21(2):174–187. 10.1038/s41593-017-0054-4 29311747

[13] To WT, De Ridder D, Hart J John, Vanneste S. Changing Brain Networks Through Non-invasive Neuromodulation. Frontiers in human neuroscience. 2018;12:128–128. 10.3389/fnhum.2018.00128 29706876 PMC5908883

[14] Luft CDB, Pereda E, Banissy MJ, Bhattacharya J. Best of both worlds: promise of combining brain stimulation and brain connectome. Frontiers in Systems Neuroscience. 2014;8:132 10.3389/fnsys.2014.00132 25126060 PMC4115621

[15] Brosch M, Budinger E, Scheich H. Stimulus-Related Gamma Oscillations in Primate Auditory Cortex. Journal of Neurophysiology. 2002;87(6):2715–2725. 10.1152/jn.2002.87.6.2715 12037173

[16] Henrie AJ, Shapley R. LFP Power Spectra in V1 Cortex: The Graded Effect of Stimulus Contrast. Journal of Neurophysiology. 2005;94(1):479–490. 10.1152/jn.00919.2004 15703230

[17] Jokisch D, Jensen O. Modulation of Gamma and Alpha Activity during a Working Memory Task Engaging the Dorsal or Ventral Stream. The Journal of Neuroscience. 2007;27(12):3244 10.1523/JNEUROSCI.5399-06.2007 17376984 PMC6672464

[18] Kristeva-Feige R, Feige B, Makeig S, Ross B, Elbert T. Oscillatory brain activity during a motor task. Neuroreport. 1992;4(12):1291–1294. 10.1097/00001756-199309150-00001 8260607

[19] Hirata A, Castro-Alamancos MA. Neocortex Network Activation and Deactivation States Controlled by the Thalamus. Journal of Neurophysiology. 2010;103(3):1147–1157. 10.1152/jn.00955.2009 20053845 PMC2887623

[20] Kastner S, Pinsk MA, De Weerd P, Desimone R, Ungerleider LG. Increased Activity in Human Visual Cortex during Directed Attention in the Absence of Visual Stimulation. Neuron. 1999;22(4):751–761. 10.1016/S0896-6273(00)80734-5 10230795

[21] Batista-Brito R, Zagha E, Ratliff JM, Vinck M. Modulation of cortical circuits by top-down processing and arousal state in health and disease. Current Opinion in Neurobiology. 2018;52:172–181. 10.1016/j.conb.2018.06.008 30064117

[22] Gazzaley A, Nobre AC. Top-down modulation: bridging selective attention and working memory. Trends in Cognitive Sciences. 2012;16(2):129–135. 10.1016/j.tics.2011.11.014 22209601 PMC3510782

[23] Hallett M. Transcranial Magnetic Stimulation: A Primer. Neuron. 2007;55(2):187–199. 10.1016/j.neuron.2007.06.026 17640522

[24] Filmer HL, Dux PE, Mattingley JB. Applications of transcranial direct current stimulation for understanding brain function. Trends in Neurosciences. 2014;37(12):742–753. 10.1016/j.tins.2014.08.003 25189102

[25] Vosskuhl J, Strüber D, Herrmann CS. Non-invasive Brain Stimulation: A Paradigm Shift in Understanding Brain Oscillations. Frontiers in Human Neuroscience. 2018;12:211 10.3389/fnhum.2018.00211 29887799 PMC5980979

[26] Thut G, Miniussi C. New insights into rhythmic brain activity from TMS–EEG studies. Trends in Cognitive Sciences. 2009;13(4):182–189. 10.1016/j.tics.2009.01.004 19286414

[27] Witkowski M, Garcia-Cossio E, Chander BS, Braun C, Birbaumer N, Robinson SE, et al Mapping entrained brain oscillations during transcranial alternating current stimulation (tACS). NeuroImage. 2016;140:89–98. 10.1016/j.neuroimage.2015.10.024 26481671

[28] Antal A, Varga ET, Kincses TZ, Nitsche MA, Paulus W. Oscillatory brain activity and transcranial direct current stimulation in humans. NeuroReport. 2004;15(8). 10.1097/01.wnr.0000127460.08361.84 15167555

[29] Neuling T, Ruhnau P, Fuscà M, Demarchi G, Herrmann CS, Weisz N. Friends, not foes: Magnetoencephalography as a tool to uncover brain dynamics during transcranial alternating current stimulation. NeuroImage. 2015;118:406–413. 10.1016/j.neuroimage.2015.06.026 26080310 PMC4686537

[30] Siebner HR, Bergmann TO, Bestmann S, Massimini M, Johansen-Berg H, Mochizuki H, et al Consensus paper: Combining transcranial stimulation with neuroimaging. Brain Stimulation. 2009;2(2):58–80. 10.1016/j.brs.2008.11.002 20633405

[31] Bortoletto M, Veniero D, Thut G, Miniussi C. The contribution of TMS–EEG coregistration in the exploration of the human cortical connectome. Neuroscience & Biobehavioral Reviews. 2015;49:114–124. 10.1016/j.neubiorev.2014.12.014 25541459

[32] Johnson MD, Lim HH, Netoff TI, Connolly AT, Johnson N, Roy A, et al Neuromodulation for Brain Disorders: Challenges and Opportunities. IEEE Transactions on Biomedical Engineering. 2013;60(3):610–624. 10.1109/TBME.2013.2244890 23380851 PMC3724171

[33] Schulz R, Gerloff C, Hummel FC. Non-invasive brain stimulation in neurological diseases. Neuropharmacology. 2013;64:579–587. 10.1016/j.neuropharm.2012.05.016 22687520

[34] Fisher RS, Velasco AL. Electrical brain stimulation for epilepsy. Nature Reviews Neurology. 2014;10(5):261–270. 10.1038/nrneurol.2014.59 24709892

[35] Muldoon SF, Pasqualetti F, Gu S, Cieslak M, Grafton ST, Vettel JM, et al Stimulation-Based Control of Dynamic Brain Networks. PLOS Computational Biology. 2016;12(9):1–23. 10.1371/journal.pcbi.1005076 PMC5017638 27611328

[36] Gollo LL, Roberts JA, Cocchi L. Mapping how local perturbations influence systems-level brain dynamics. NeuroImage. 2017;160:97–112. 10.1016/j.neuroimage.2017.01.057 28126550

[37] Spiegler A, Hansen ECA, Bernard C, McIntosh AR, Jirsa VK. Selective Activation of Resting-State Networks following Focal Stimulation in a Connectome-Based Network Model of the Human Brain. eNeuro. 2016;3(5):ENEURO.0068–16.2016. 10.1523/ENEURO.0068-16.2016 27752540 PMC5052665

[38] Kunze T, Hunold A, Haueisen J, Jirsa V, Spiegler A. Transcranial direct current stimulation changes resting state functional connectivity: A large-scale brain network modeling study. NeuroImage. 2016;140:174–187. 10.1016/j.neuroimage.2016.02.015 26883068

[39] Witt A, Palmigiano A, Neef A, El Hady A, Wolf F, Battaglia D. Controlling the oscillation phase through precisely timed closed-loop optogenetic stimulation: a computational study. Frontiers in Neural Circuits. 2013;7:49 10.3389/fncir.2013.00049 23616748 PMC3627980

[40] Kirst C, Timme M, Battaglia D. Dynamic information routing in complex networks. Nature Communications. 2016;7(1):11061 10.1038/ncomms11061 27067257 PMC4832059

[41] Stiso J, Khambhati AN, Menara T, Kahn AE, Stein JM, Das SR, et al White Matter Network Architecture Guides Direct Electrical Stimulation through Optimal State Transitions. Cell Reports. 2019;28(10):2554–2566.e7. 10.1016/j.celrep.2019.08.008 31484068 PMC6849479

[42] Khambhati AN, Kahn AE, Costantini J, Ezzyat Y, Solomon EA, Gross RE, et al Functional control of electrophysiological network architecture using direct neurostimulation in humans. Network Neuroscience. 2019;3(3):848–877. 10.1162/netn_a_00089 31410383 PMC6663306

[43] Bergmann TO. Brain State-Dependent Brain Stimulation. Frontiers in Psychology. 2018;9:2108 10.3389/fpsyg.2018.02108 30443236 PMC6221926

[44] Thut G, Bergmann TO, Frohlich F, Soekadar SR, Brittain JS, Valero-Cabré A, et al Guiding transcranial brain stimulation by EEG/MEG to interact with ongoing brain activity and associated functions: A position paper. Clinical Neurophysiology. 2017;128(5):843–857. 10.1016/j.clinph.2017.01.003 28233641 PMC5385293

[45] Silvanto J, Pascual-Leone A. State-dependency of transcranial magnetic stimulation. Brain topography. 2008;21(1):1–10. 10.1007/s10548-008-0067-0 18791818 PMC3049188

[46] Neuling T, Rach S, Herrmann C. Orchestrating neuronal networks: sustained after-effects of transcranial alternating current stimulation depend upon brain states. Frontiers in Human Neuroscience. 2013;7:161 10.3389/fnhum.2013.00161 23641206 PMC3639376

[47] Ruhnau P, Neuling T, Fusca M, Herrmann CS, Demarchi G, Weisz N. Eyes wide shut: Transcranial alternating current stimulation drives alpha rhythm in a state dependent manner. Scientific Reports. 2016;6(1):27138 10.1038/srep27138 27252047 PMC4890046

[48] Alagapan S, Schmidt SL, Lefebvre J, Hadar E, Shin HW, Frohlich F. Modulation of Cortical Oscillations by Low-Frequency Direct Cortical Stimulation Is State-Dependent. PLOS Biology. 2016;14(3):e1002424–. 10.1371/journal.pbio.1002424 27023427 PMC4811434

[49] Lefebvre J, Hutt A, Frohlich F, Haegens S. Stochastic resonance mediates the state-dependent effect of periodic stimulation on cortical alpha oscillations. eLife. 2017;6:e32054 10.7554/eLife.32054 29280733 PMC5832422

[50] Li G, Henriquez CS, Frohlich F. Unified thalamic model generates multiple distinct oscillations with state-dependent entrainment by stimulation. PLOS Computational Biology. 2017;13(10):e1005797–. 10.1371/journal.pcbi.1005797 29073146 PMC5675460

[51] Breakspear M. Dynamic models of large-scale brain activity. Nat Neurosci. 2017;20(3):340–352. 10.1038/nn.4497 28230845

[52] Atasoy S, Donnelly I, Pearson J. Human brain networks function in connectome-specific harmonic waves. Nature Communications. 2016;7(1):10340 10.1038/ncomms10340 26792267 PMC4735826

[53] Wilson HR, Cowan JD. Excitatory and Inhibitory Interactions in Localized Populations of Model Neurons. Biophysical Journal. 1972;12(1):1–24. 10.1016/S0006-3495(72)86068-5 4332108 PMC1484078

[54] Betzel RF, Bassett DS. Specificity and robustness of long-distance connections in weighted, interareal connectomes. Proceedings of the National Academy of Sciences. 2018 10.1073/pnas.1720186115 PMC6003515 29739890

[55] Betzel RF, Medaglia JD, Papadopoulos L, Baum GL, Gur R, Gur R, et al The modular organization of human anatomical brain networks: Accounting for the cost of wiring. Network Neuroscience. 2017;1(1):42–68. 10.1162/NETN_a_00002 30793069 PMC6372290

[56] Betzel RF, Medaglia JD, Bassett DS. Diversity of meso-scale architecture in human and non-human connectomes. Nature Communications. 2018;9(1):346 10.1038/s41467-017-02681-z 29367627 PMC5783945

[57] Medaglia JD, Harvey DY, White N, Kelkar A, Zimmerman J, Bassett DS, et al Network Controllability in the Inferior Frontal Gyrus Relates to Controlled Language Variability and Susceptibility to TMS. J Neurosci. 2018;38(28):6399–6410. 10.1523/JNEUROSCI.0092-17.2018 29884739 PMC6041793

[58] Betzel RF, Gu S, Medaglia JD, Pasqualetti F, Bassett DS. Optimally controlling the human connectome: the role of network topology. Scientific Reports. 2016;6(1):30770 10.1038/srep30770 27468904 PMC4965758

[59] Medaglia JD, Huang W, Karuza EA, Kelkar A, Thompson-Schill SL, Ribeiro A, et al Functional alignment with anatomical networks is associated with cognitive flexibility. Nature Human Behaviour. 2018;2(2):156–164. 10.1038/s41562-017-0260-9 30498789 PMC6258039

[60] Yeh FC, Wedeen VJ, Tseng WYI. Estimation of fiber orientation and spin density distribution by diffusion deconvolution. NeuroImage. 2011;55(3):1054—1062. 10.1016/j.neuroimage.2010.11.087 21232611

[61] Frackowiak RSJ, Friston KJ, Frith CD, Dolan RJ, Price CJ, Zeki S, et al In: Chapter 31—Experimental Design and Statistical Parametric Mapping. Burlington: Academic Press; 2004 p. 599–632. Available from: http://www.sciencedirect.com/science/article/pii/B9780122648410500330.

[62] Mori S, van Zijl PCM. Fiber tracking: principles and strategies–a technical review. NMR in Biomedicine. 2002;15(7–8):468–480. 10.1002/nbm.781 12489096

[63] Gu S, Pasqualetti F, Cieslak M, Telesford QK, Yu AB, Kahn AE, et al Controllability of structural brain networks. Nature Communications. 2015;6(1):8414 10.1038/ncomms9414 26423222 PMC4600713

[64] Fischl B. FreeSurfer. NeuroImage. 2012;62(2):774–781. 10.1016/j.neuroimage.2012.01.021 22248573 PMC3685476

[65] Cammoun L, Gigandet X, Meskaldji D, Thiran JP, Sporns O, Do KQ, et al Mapping the human connectome at multiple scales with diffusion spectrum MRI. Journal of Neuroscience Methods. 2012;203(2):386–397. 10.1016/j.jneumeth.2011.09.031 22001222

[66] Abeysuriya RG, Hadida J, Sotiropoulos SN, Jbabdi S, Becker R, Hunt BAE, et al A biophysical model of dynamic balancing of excitation and inhibition in fast oscillatory large-scale networks. PLOS Computational Biology. 2018;14(2):1–27. 10.1371/journal.pcbi.1006007 PMC5841816 29474352

[67] Cabral J, Hugues E, Sporns O, Deco G. Role of local network oscillations in resting-state functional connectivity. NeuroImage. 2011;57(1):130–139. 10.1016/j.neuroimage.2011.04.010 21511044

[68] Tewarie P, Hunt BAE, O’Neill GC, Byrne A, Aquino K, Bauer M, et al Relationships Between Neuronal Oscillatory Amplitude and Dynamic Functional Connectivity. Cerebral Cortex. 2018;29(6):2668–2681. 10.1093/cercor/bhy136 29897408

[69] Cabral J, Luckhoo H, Woolrich M, Joensson M, Mohseni H, Baker A, et al Exploring mechanisms of spontaneous functional connectivity in MEG: How delayed network interactions lead to structured amplitude envelopes of band-pass filtered oscillations. NeuroImage. 2014;90:423–435. 10.1016/j.neuroimage.2013.11.047 24321555

[70] Kale P, Zalesky A, Gollo LL. Estimating the impact of structural directionality: How reliable are undirected connectomes? Network neuroscience (Cambridge, Mass). 2018;2(2):259–284. 10.1162/netn_a_00040 PMC6135560 30234180

[71] Stiso J, Bassett DS. Spatial Embedding Imposes Constraints on Neuronal Network Architectures. Trends in Cognitive Sciences. 2018;22(12):1127–1142. 10.1016/j.tics.2018.09.007 30449318

[72] Ritter P, Schirner M, McIntosh AR, Jirsa VK. The Virtual Brain Integrates Computational Modeling and Multimodal Neuroimaging. Brain Connectivity. 2013;3(2):121–145. 10.1089/brain.2012.0120 23442172 PMC3696923

[73] Deco G, Jirsa V, McIntosh AR, Sporns O, Kötter R. Key role of coupling, delay, and noise in resting brain fluctuations. Proceedings of the National Academy of Sciences. 2009;106(25):10302–10307. 10.1073/pnas.0901831106 PMC2690605 19497858

[74] Honey CJ, Sporns O, Cammoun L, Gigandet X, Thiran JP, Meuli R, et al Predicting human resting-state functional connectivity from structural connectivity. Proceedings of the National Academy of Sciences. 2009;106(6):2035–2040. 10.1073/pnas.0811168106 PMC2634800 19188601

[75] Hlinka J, Coombes S. Using computational models to relate structural and functional brain connectivity. The European Journal of Neuroscience. 2012;36(2):2137–2145. 10.1111/j.1460-9568.2012.08081.x 22805059 PMC3437497

[76] Roberts JA, Gollo LL, Abeysuriya RG, Roberts G, Mitchell PB, Woolrich MW, et al Metastable brain waves. Nature Communications. 2019;10(1):1056 10.1038/s41467-019-08999-0 30837462 PMC6401142

[77] Gollo LL, Zalesky A, Hutchison RM, van den Heuvel M, Breakspear M. Dwelling quietly in the rich club: brain network determinants of slow cortical fluctuations. Philosophical Transactions of the Royal Society of London B: Biological Sciences. 2015;370 (1668). 10.1098/rstb.2014.0165 25823864 PMC4387508

[78] Bansal K, Nakuci J, Muldoon SF. Personalized brain network models for assessing structure–function relationships. Current Opinion in Neurobiology. 2018;52:42–47. 10.1016/j.conb.2018.04.014 29704749

[79] Bansal K, Garcia JO, Tompson SH, Verstynen T, Vettel JM, Muldoon SF. Cognitive chimera states in human brain networks. Science Advances. 2019;5(4). 10.1126/sciadv.aau8535 PMC6447382 30949576

[80] Sanz-Leon P, Knock SA, Spiegler A, Jirsa VK. Mathematical framework for large-scale brain network modeling in The Virtual Brain. NeuroImage. 2015;111:385–430. 10.1016/j.neuroimage.2015.01.002 25592995

[81] Glomb K, Ponce-Alvarez A, Gilson M, Ritter P, Deco G. Resting state networks in empirical and simulated dynamic functional connectivity. NeuroImage. 2017;159:388–402. 10.1016/j.neuroimage.2017.07.065 28782678

[82] Vuksanović V, Hövel P. Dynamic changes in network synchrony reveal resting-state functional networks. Chaos: An Interdisciplinary Journal of Nonlinear Science. 2015;25(2):023116 10.1063/1.4913526 25725652

[83] Murray JD, Demirtas M, Anticevic A. Biophysical Modeling of Large-Scale Brain Dynamics and Applications for Computational Psychiatry. Biological Psychiatry: Cognitive Neuroscience and Neuroimaging. 2018;3(9):777–787. 10.1016/j.bpsc.2018.07.004. 30093344 PMC6537601

[84] Demirtas M, Burt JB, Helmer M, Ji JL, Adkinson BD, Glasser MF, et al Hierarchical heterogeneity across human cortex shapes large-scale neural dynamics. bioRxiv. 2018;. 10.1016/j.neuron.2019.01.017 PMC6447428 30744986

[85] Börgers C, Kopell N. Synchronization in Networks of Excitatory and Inhibitory Neurons with Sparse, Random Connectivity. Neural Computation. 2003;15(3):509–538. 10.1162/089976603321192059 12620157

[86] Kopell N, Börgers C, Pervouchine D, Malerba P, Tort A. In: Cutsuridis V, Graham B, Cobb S, Vida I, editors. Gamma and Theta Rhythms in Biophysical Models of Hippocampal Circuits. New York, NY: Springer New York; 2010 p. 423–457. Available from: 10.1007/978-1-4419-0996-1_15.

[87] Palmigiano A, Geisel T, Wolf F, Battaglia D. Flexible information routing by transient synchrony. Nature Neuroscience. 2017;20:1014 EP–. 10.1038/nn.4569 28530664

[88] Battaglia D, Witt A, Wolf F, Geisel T. Dynamic Effective Connectivity of Inter-Areal Brain Circuits. PLOS Computational Biology. 2012;8(3):1–20. 10.1371/journal.pcbi.1002438 22457614 PMC3310731

[89] Mejias JF, Murray JD, Kennedy H, Wang XJ. Feedforward and feedback frequency-dependent interactions in a large-scale laminar network of the primate cortex. Science Advances. 2016;2(11). 10.1126/sciadv.1601335 28138530 PMC5262462

[90] Lachaux JP, Rodriguez E, Martinerie J, Varela FJ. Measuring phase synchrony in brain signals. Hum Brain Mapp. 1999;8(4):194–208. 10.1002/(SICI)1097-0193(1999)8:4<194::AID-HBM4>3.0.CO;2-C 10619414 PMC6873296

[91] Pikovsky A, Rosenblum M, Kurths J. Synchronization: a universal concept in nonlinear sciences. Cambridge university press; 2003.

[92] Lowet E, Roberts MJ, Bonizzi P, Karel J, De Weerd P. Quantifying Neural Oscillatory Synchronization: A Comparison between Spectral Coherence and Phase-Locking Value Approaches. PLOS ONE. 2016;11(1):e0146443–. 10.1371/journal.pone.0146443 26745498 PMC4706353

[93] Hoppensteadt FC, Izhikevich EM. Weakly connected neural networks. vol. 126 Springer Science and Business Media; 2012.

[94] Lee SH, Dan Y. Neuromodulation of brain states. Neuron. 2012;76(1):209–222. 10.1016/j.neuron.2012.09.012 23040816 PMC3579548

[95] Li M, Han Y, Aburn MJ, Breakspear M, Poldrack RA, Shine JM, et al Transitions in information processing dynamics at the whole-brain network level are driven by alterations in neural gain. PLOS Computational Biology. 2019;15(10):e1006957–. 10.1371/journal.pcbi.1006957 31613882 PMC6793849

[96] Shine JM. Neuromodulatory Influences on Integration and Segregation in the Brain. Trends in Cognitive Sciences. 2019;23(7):572–583. 10.1016/j.tics.2019.04.002 31076192

[97] Aston-Jones G, Cohen JD. AN INTEGRATIVE THEORY OF LOCUS COERULEUS-NOREPINEPHRINE FUNCTION: Adaptive Gain and Optimal Performance. Annual Review of Neuroscience. 2005;28(1):403–450. 10.1146/annurev.neuro.28.061604.135709 16022602

[98] Rubinov M, Sporns O. Complex network measures of brain connectivity: Uses and interpretations. NeuroImage. 2010;52(3):1059–1069. 10.1016/j.neuroimage.2009.10.003 19819337

[99] Kopell NJ, Gritton HJ, Whittington MA, Kramer MA. Beyond the Connectome: The Dynome. Neuron. 2014;83(6):1319–1328. 10.1016/j.neuron.2014.08.016 25233314 PMC4169213

[100] Bargmann CI, Marder E. From the connectome to brain function. Nature Methods. 2013;10(6):483–490. 10.1038/nmeth.2451 23866325

[101] Lowet E, Roberts M, Hadjipapas A, Peter A, van der Eerden J, De Weerd P. Input-Dependent Frequency Modulation of Cortical Gamma Oscillations Shapes Spatial Synchronization and Enables Phase Coding. PLOS Computational Biology. 2015;11(2):1–44. 10.1371/journal.pcbi.1004072 PMC4334551 25679780

[102] Jia X, Xing D, Kohn A. No Consistent Relationship between Gamma Power and Peak Frequency in Macaque Primary Visual Cortex. Journal of Neuroscience. 2013;33(1):17–25. 10.1523/JNEUROSCI.1687-12.2013 23283318 PMC3560843

[103] Schuster HG, Just W. Deterministic chaos: an introduction. John Wiley & Sons; 2006.

[104] Bell PT, Shine JM. Subcortical contributions to large-scale network communication. Neuroscience & Biobehavioral Reviews. 2016;71:313–322. 10.1016/j.neubiorev.2016.08.036 27590830

[105] Tang E, Bassett DS. Colloquium: Control of dynamics in brain networks. Reviews of Modern Physics. 2018;90(3):031003–. 10.1103/RevModPhys.90.031003

[106] Canolty RT, Knight RT. The functional role of cross-frequency coupling. Trends in cognitive sciences. 2010;14(11):506–515. 10.1016/j.tics.2010.09.001 20932795 PMC3359652

[107] Daffertshofer A, van Wijk BCM. On the Influence of Amplitude on the Connectivity between Phases. Frontiers in Neuroinformatics. 2011;5:6 10.3389/fninf.2011.00006 21811452 PMC3139941

[108] Brookes MJ, Wood JR, Stevenson CM, Zumer JM, White TP, Liddle PF, et al Changes in brain network activity during working memory tasks: A magnetoencephalography study. NeuroImage. 2011;55(4):1804–1815. 10.1016/j.neuroimage.2010.10.074 21044687 PMC6485426

[109] Ahrens KF, Levine H, Suhl H, Kleinfeld D. Spectral mixing of rhythmic neuronal signals in sensory cortex. Proceedings of the National Academy of Sciences. 2002;99(23):15176–15181. 10.1073/pnas.222547199 PMC137563 12403828

[110] Battaglia D, Brunel N, Hansel D. Temporal Decorrelation of Collective Oscillations in Neural Networks with Local Inhibition and Long-Range Excitation. Phys Rev Lett. 2007;99(23):238106 10.1103/PhysRevLett.99.238106 18233419

[111] Hermundstad AM, Bassett DS, Brown KS, Aminoff EM, Clewett D, Freeman S, et al Structural foundations of resting-state and task-based functional connectivity in the human brain. Proceedings of the National Academy of Sciences. 2013;110(15):6169–6174. 10.1073/pnas.1219562110 PMC3625268 23530246

[112] Shen K, Hutchison RM, Bezgin G, Everling S, McIntosh AR. Network Structure Shapes Spontaneous Functional Connectivity Dynamics. The Journal of Neuroscience. 2015;35(14):5579 10.1523/JNEUROSCI.4903-14.2015 25855174 PMC6605321

[113] Shen K, Mišić B, Cipollini BN, Bezgin G, Buschkuehl M, Hutchison RM, et al Stable long-range interhemispheric coordination is supported by direct anatomical projections. Proceedings of the National Academy of Sciences. 2015;112(20):6473 10.1073/pnas.1503436112 PMC4443345 25941372

[114] Avena-Koenigsberger A, Misic B, Sporns O. Communication dynamics in complex brain networks. Nature Reviews Neuroscience. 2018;19(1):17–33. 10.1038/nrn.2017.149 29238085

[115] Vázquez-Rodríguez B, Suárez LE, Markello RD, Shafiei G, Paquola C, Hagmann P, et al Gradients of structure–function tethering across neocortex. Proceedings of the National Academy of Sciences. 2019;116(42):21219 10.1073/pnas.1903403116 PMC6800358 31570622

[116] Bargmann CI. Beyond the connectome: How neuromodulators shape neural circuits. BioEssays. 2012;34(6):458–465. 10.1002/bies.201100185 22396302

[117] Gutierrez GJ, Marder E. Modulation of a Single Neuron Has State-Dependent Actions on Circuit Dynamics. eneuro. 2014;1(1):ENEURO.0009–14.2014. 10.1523/ENEURO.0009-14.2014 26457324 PMC4596081

[118] Li LM, Violante IR, Leech R, Ross E, Hampshire A, Opitz A, et al Brain state and polarity dependent modulation of brain networks by transcranial direct current stimulation. Human Brain Mapping. 2019;40(3):904–915. 10.1002/hbm.24420 30378206 PMC6387619

[119] Stefanescu RA, Shivakeshavan RG, Talathi SS. Computational models of epilepsy. Seizure. 2012;21(10):748–759. 10.1016/j.seizure.2012.08.012 22995680

[120] Kameneva T, Ying T, Guo B, Freestone DR. Neural mass models as a tool to investigate neural dynamics during seizures. Journal of Computational Neuroscience. 2017;42(2):203–215. 10.1007/s10827-017-0636-x 28102460

[121] Lazar M. Mapping brain anatomical connectivity using white matter tractography. NMR in biomedicine. 2010;23(7):821–835. 10.1002/nbm.1579 20886567 PMC4503207

[122] Bansal K, Medaglia JD, Bassett DS, Vettel JM, Muldoon SF. Data-driven brain network models differentiate variability across language tasks. PLoS computational biology. 2018;14(10):e1006487–e1006487. 10.1371/journal.pcbi.1006487 30332401 PMC6192563

[123] Triebkorn P, Zimmermann J, Stefanovski L, Roy D, Solodkin A, Jirsa V, et al Identifying optimal working points of individual Virtual Brains: A large-scale brain network modelling study. bioRxiv. 2020.

[124] Schmidt M, Bakker R, Hilgetag CC, Diesmann M, van Albada SJ. Multi-scale account of the network structure of macaque visual cortex. Brain Structure and Function. 2018;223(3):1409–1435. 10.1007/s00429-017-1554-4 29143946 PMC5869897

[125] Honey CJ, Kötter R, Breakspear M, Sporns O. Network structure of cerebral cortex shapes functional connectivity on multiple time scales. Proceedings of the National Academy of Sciences. 2008;104(24):10240–10245. 10.1073/pnas.0701519104 PMC1891224 17548818

[126] Zalesky A, Fornito A, Cocchi L, Gollo LL, Breakspear M. Time-resolved resting-state brain networks. Proceedings of the National Academy of Sciences. 2014;111(28):10341–10346. 10.1073/pnas.1400181111 PMC4104861 24982140

[127] Shine JM, Poldrack RA. Principles of dynamic network reconfiguration across diverse brain states. NeuroImage. 2018;180:396–405. 10.1016/j.neuroimage.2017.08.010 28782684

[128] Petkoski S, Jirsa VK. Transmission time delays organize the brain network synchronization. Philosophical Transactions of the Royal Society A: Mathematical, Physical and Engineering Sciences. 2019;377(2153):20180132 10.1098/rsta.2018.0132 PMC6661323 31329065

[129] Schmidt R, Herrojo Ruiz M, Kilavik BE, Lundqvist M, Starr PA, Aron AR. Beta Oscillations in Working Memory, Executive Control of Movement and Thought, and Sensorimotor Function. Journal of Neuroscience. 2019;39(42):8231–8238. 10.1523/JNEUROSCI.1163-19.2019 31619492 PMC6794925

[130] Hughes SW, Crunelli V. Thalamic mechanisms of EEG alpha rhythms and their pathological implications. Neuroscientist. 2005;11(4):357–372. 10.1177/1073858405277450 16061522

[131] Freyer F, Roberts JA, Becker R, Robinson PA, Ritter P, Breakspear M. Biophysical Mechanisms of Multistability in Resting-State Cortical Rhythms. The Journal of Neuroscience. 2011;31(17):6353 10.1523/JNEUROSCI.6693-10.2011 21525275 PMC6622680

[132] Murray JD, Bernacchia A, Freedman DJ, Romo R, Wallis JD, Cai X, et al A hierarchy of intrinsic timescales across primate cortex. Nature Neuroscience. 2014;17(12):1661–1663. 10.1038/nn.3862 25383900 PMC4241138

[133] Buffalo EA, Fries P, Landman R, Buschman TJ, Desimone R. Laminar differences in gamma and alpha coherence in the ventral stream. Proceedings of the National Academy of Sciences. 2011;108(27):11262 10.1073/pnas.1011284108 PMC3131344 21690410

[134] Deco G, Cruzat J, Cabral J, Tagliazucchi E, Laufs H, Logothetis NK, et al Awakening: Predicting external stimulation to force transitions between different brain states. Proceedings of the National Academy of Sciences. 2019;116(36):18088 10.1073/pnas.1905534116 PMC6731634 31427539

[135] Castro S, El-Deredy W, Battaglia D, Orio P. Cortical ignition dynamics is tightly linked to the core organisation of the human connectome. bioRxiv. 2020. 10.1371/journal.pcbi.1007686 PMC7423150 32735580

[136] Cornelius SP, Kath WL, Motter AE. Realistic control of network dynamics. Nature Communications. 2013;4(1):1942 10.1038/ncomms2939 23803966 PMC3955710

[137] Reato D, Rahman A, Bikson M, Parra L. Effects of weak transcranial alternating current stimulation on brain activity—a review of known mechanisms from animal studies. Frontiers in Human Neuroscience. 2013;7:687 10.3389/fnhum.2013.00687 24167483 PMC3805939

[138] Shine JM, Aburn MJ, Breakspear M, Poldrack RA. The modulation of neural gain facilitates a transition between functional segregation and integration in the brain. eLife. 2018;7:e31130 10.7554/eLife.31130 29376825 PMC5818252

