# Supplementary material for: Relations between large-scale brain connectivity and effects of regional stimulation depend on collective dynamical state
Source: PLoS Comput Biol. 2020 Sep 4;16(9):e1008144. doi: 10.1371/journal.pcbi.1008144 (PMC7537889; doi:10.1371/journal.pcbi.1008144)
Supplement: S1 Text — (PDF) [file pcbi.1008144.s001.pdf]

SUPPLEMENTARY TEXT

"Relations between large-scale brain connectivity and effects of regional stimulation depend on collective dynamical state"

Lia Papadopoulos[1], Christopher W. Lynn[1], Demian Battaglia[7], Danielle S. Bassett[1,2,3,4,5,6*],

1 Department of Physics & Astronomy, University of Pennsylvania, Philadelphia, PA, USA

2 Department of Bioengineering, University of Pennsylvania, Philadelphia, PA, USA

3 Department of Electrical & Systems Engineering, University of Pennsylvania, Philadelphia, PA, USA

4 Department of Neurology, University of Pennsylvania, Philadelphia, PA, USA

5 Department of Psychiatry, University of Pennsylvania, Philadelphia, PA, USA

6 Santa Fe Institute, Santa Fe, NM, USA

7 Institute for Systems Neuroscience, Aix-Marseille University, Marseille, France

* dsb@seas.upenn.edu

SI Dynamics of an isolated Wilson-Cowan unit

In this section we briefly describe the behavior of a single Wilson-Cowan (WC) unit, which forms the fundamental dynamical component of the whole-brain computational model. The excitatory and inhibitory pools of an isolated WC unit evolve according to Eqs. ?? and ??, with the coupling term set to $C = 0$. Here we take all other model parameters to be those displayed in Table ??, with the exception that we consider noiseless simulations for the purpose of demonstration.

A typical bifurcation parameter for the WC model is the drive P_E to the excitatory population; when other parameters are appropriately tuned, varying P_E can induce oscillatory activity. The top row of Fig. A shows phase plane representations of a single WC unit for three different levels of the input P_E, and the bottom row of each panel shows the time-evolution of the excitatory activity $E(t)$ for the given parameter value. In the phase planes, the blue lines correspond to the excitatory variable nullcline ($dE/dt = 0$), the red lines correspond to the inhibitory variable nullcline ($dI/dt = 0$), and the purple lines show an example trajectory that begins at the point denoted by the star. For a low input level of $P_E = 0.6$ (panel A), the system has a single stable fixed-point corresponding to a low activity steady-state. For an intermediate drive of $P_E = 1.25$ (panel B), the system exhibits a stable limit cycle and the firing-rate activity oscillates in time. For a high input of $P_E = 3$ (panel C), the system again exhibits a single stable fixed-point, but corresponding to a high activity steady-state.

To summarize the effect of the drive P_E on the behavior of an isolated WC unit, we first plot the time-average of the excitatory firing-rate $\overline{E(t)}$ as a function of the input P_E (Fig. A, panel D). Note that $\overline{E(t)}$ increases with increasing drive. Second, we consider how the peak frequency f^{peak} of the excitatory activity varies with the input level P_E (Fig. A, panel E), where the peak frequency is that for which the Welch's power spectral density of the excitatory time-series is maximum (see ?? for details). For low inputs, f^{peak} is approximately zero; the system resides in the low activity steady-state and there are no intrinsic oscillations. As the input is increased, though, oscillations emerge with frequencies in the gamma range (30 - 70 Hz). Increasing the input P_E in the oscillatory regime first raises the peak frequency from its initial value up to ∼65Hz. Such an increase in oscillatory frequency with increasing drive has also been found experimentally [1–4]. However, beyond a certain point, further increasing the excitatory drive causes the peak frequency to decline back to zero as the system approaches the high activity fixed point where oscillations again cease completely. In Fig. A, panel F, we show excitatory activity time-series for three different values of the input P_E that place the system in the oscillatory regime. It is clear by eye that for these parameters, increasing the excitatory drive increases the amplitude and frequency of the oscillations.

SII Determining the onset of oscillatory activity in the whole-brain model

In order to systematically determine the boundary marking the onset of oscillatory activity as a function of the background drive P_E^{base} and the coupling C, we examine the network-average of the standard deviation of the excitatory activity across time, $\langle \mathrm{std}[E_i(t)] \rangle$. This quantity measures the strength of fluctuations of the excitatory population activities around their mean values. Thus, by noting when $\langle \mathrm{std}[E_i(t)] \rangle$ jumps from a value near zero to a higher, positive value, we can qualitatively determine the transition from the state of low, non-oscillatory firing-rates to the onset of rhythmic dynamics in regional activity. Here, we are interested in finding the level of background excitation $P_E^*(C)$ that is needed to induce oscillations at each brain area for a given interareal coupling C. To determine these "boundary" points $P_E^*(C)$, we thus hold C fixed, and consider the difference in $\langle \mathrm{std}[E_i(t)] \rangle$ between consecutive values of P_E^{base}. We plot this difference $\Delta \langle \mathrm{std}[E_i(t)] \rangle$ as a function of P_E^{base} and C in Fig. B, where we indeed observe a clear boundary separating the low-activity and oscillatory regimes. In particular, we define the border point for each coupling C as the value $P_E^*(C)$ for which the difference $\Delta \langle \mathrm{std}[E_i(t)] \rangle$ is maximized. We mark the boundary corresponding to the onset of oscillatory activity with red squares in Fig. B.

SIII Working point 2: Global coherence peak

In the main text, we examined in detail the effects of focal perturbations for two distinct working points – WP1 and WP3 – corresponding to low and high background drive states situated below and above peak global coherence, respectively. In this section, we also consider the effects of perturbations at WP2, for which $P_E^{\mathrm{base}} = 0.57$ (the coupling is kept at $C = 2.5$). Here, the background input is intermediately valued, and the system resides at approximately the state of peak ρ^{global} (see Fig. ??A). We thus observe blocks of relatively strong phase-locking in the baseline PLV matrix (Fig. ??C, Row 2, Column 1) at this working point. However, note that the network-averaged PLV is only ≈ 0.57, which is still significantly less than the maximum possible value of $\rho^{\mathrm{global}} = 1$.

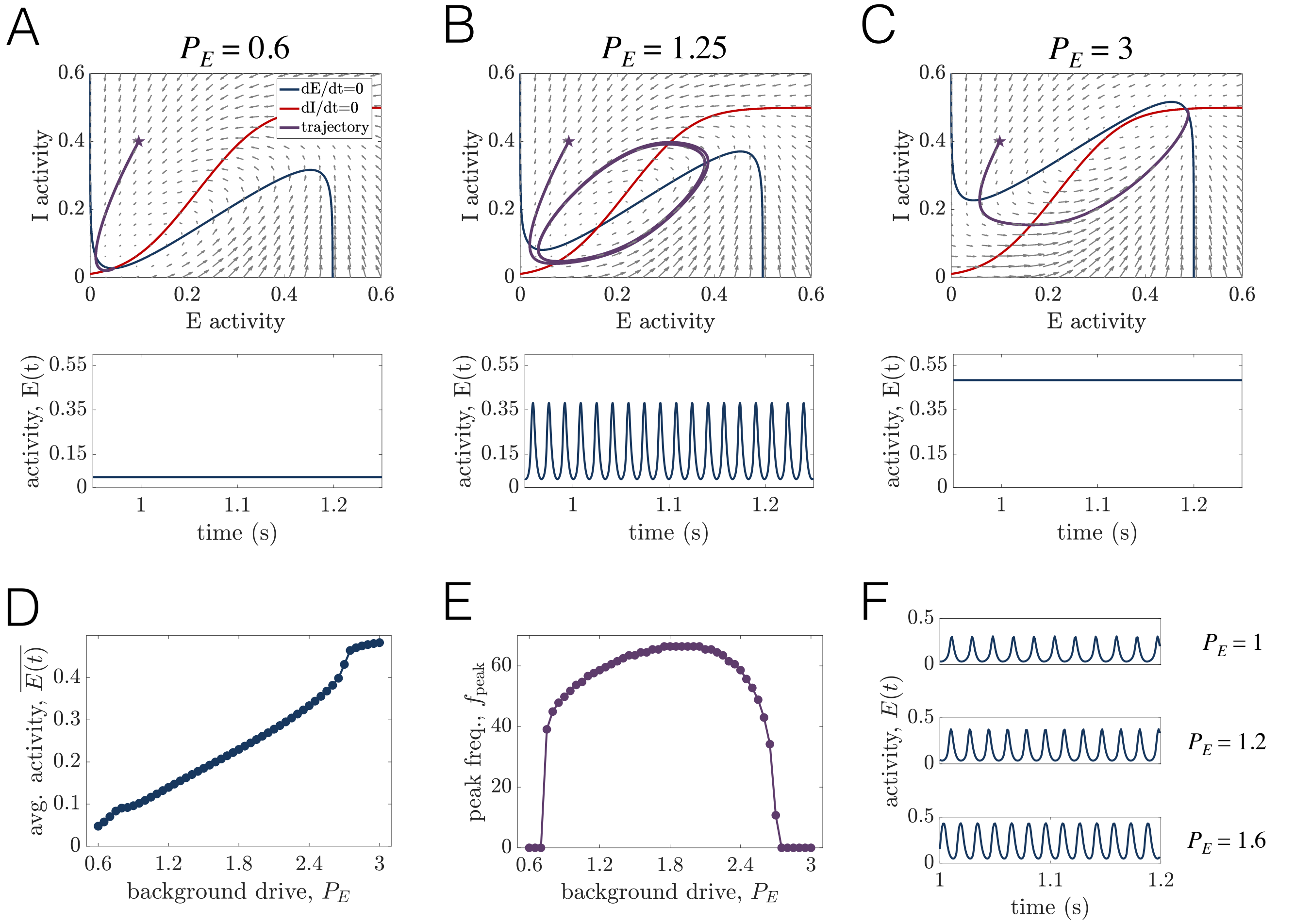

Fig A. Behavior of an isolated Wilson-Cowan unit. *(A-C)* Phase plane representations (top) and excitatory time series (bottom) for an isolated WC unit subject to varying levels of excitatory drive: $P_E = 0.6$ (A), $P_E = 1.25$ (B), and $P_E = 3.0$ (C). *(D)* The time-average of the excitatory firing-rate $\overline{E(t)}$ as a function of the input P_E for a single Wilson-Cowan unit. *(E)* The peak frequency f^{peak} of the excitatory activity as a function of the input P_E for a single Wilson-Cowan unit. *(F)* Examples of excitatory firing-rate activity for three different values of the input ($P_E = \{1.0, 1.2, 1.6\}$ from top to bottom) that place the system in the oscillatory regime.

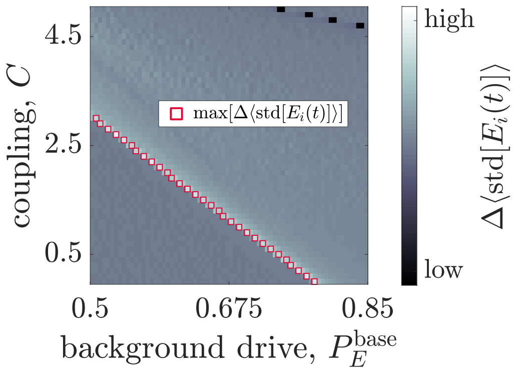

Fig B. The change in the network-average of the standard deviation of excitatory activity across time, $\Delta\langle\text{std}[E_i(t)]\rangle$, as a function of the global coupling C and the non-specific background drive P_E^{base}. We compute the change in $\langle\text{std}[E_i(t)]\rangle$ as P_E^{base} varies by taking the difference of this quantity between consecutive values of the input P_E^{base}, while holding the coupling C fixed. The red squares denote the values $P_E^*(C)$ at which $\Delta\langle\text{std}[E_i(t)]\rangle$ is largest for each coupling C. These points delineate a transition in regional brain dynamics from a quiescent state to a state of oscillatory activity.

SIII.1 Spectral modifications in baseline and excited bands persist at state of peak coherence

Similar to WP1, stimulation of region i causes an increase in the amplitude and frequency of its activity (Fig. C, panel B and Fig. C, panel C, Left). Depending on the stimulated site, the induced shift in peak frequency $\Delta f_{i,\delta_i}^{\text{peak}}$ ranges between approximately 5 and 15 Hz (Fig. C, panel D). Consequently, there is a clear separation between the distribution of units' peak frequencies in the baseline condition and when excited with additional input (Fig. C, panel E), indicating that there are again two frequency ranges of interest for further analysis. Due to interactions with the network, the excited area's spectra also develops sidebands to the left and right of its main peak, and a second bump at a frequency equal to the difference in the sideband and peak frequencies. This latter feature reflects the enhanced amplitude modulations that emerge in the time-series of unit i under stimulation (Fig. C, panel B, Right) and is indicative of quasiperiodic dynamics in the network. We also examine the power spectra of two downstream regions j and k located at increasing topological distances (hence receiving progressively weaker structural input) from the perturbed area i (Fig. C, panel C, Middle, Right). As for WP1, downstream region j, which receives strong input from i, develops a new spectral component at the main frequency of the excited region, and also at a lower frequency approximately equal to the difference of its baseline peak and the excited peak. On the other hand, unit k, which is more weakly connected to i, does not exhibit these same modulations. In Sec. SV of **??**, we show that the relationship between the strength of the power modulation at the peak frequency of the stimulated site i and the topological distance from i to the dowstream unit holds across different choices of the excited area.

To see more generally how focal stimulation can modulate downstream spectra, we compute the average spectra $\langle \text{psd} \rangle_{j \neq i}$ over all units $j \neq i$ in the baseline state and in the state when unit i is selectively excited (Fig. C, panel F) and the average difference $\langle \Delta\text{psd}_{j,\delta_i} \rangle_{j \neq i}$ of the spectra of unit $j \neq i$ between when unit i is stimulated and in the baseline condition (Fig. C, panel G). We again observe power modulations in both the baseline frequency band and in an excited frequency band centered around the peak frequency of the directly stimulated region. However, for the perturbed area studied in the example shown at WP1 (Fig. **??**F-G) and again here at WP2 (Fig. C, panels F-G), the maximum relative power modulations are weaker in the latter case. In general, it is important to note that the strength of the observable changes to the power spectra induced by focal perturbations are dependent on the choice of the excited area and on the system's working point. In the next section, we proceed to examine changes in the PLV in the baseline and excited bands – $\Delta\rho_{\delta_i}^{\text{base}}$ and $\Delta\rho_{\delta_i}^{\text{exc}}$ – induced by regional perturbations.

SIII.2 Structural and functional network connectivity continue to predict overall changes in excited and baseline band phase-locking at the peak-coherence working point

Akin to WP1, local stimulation at WP2 induces phase-locking changes that differ depending on which part of the network is perturbed and which frequency band is examined (Fig. D, panels A–D). To appreciate this fact, it is helpful to study examples that display the changes in the PLV in both the baseline band and the excited band when either region i or region $j \neq i$ is excited (Fig. D, panels A,C). To characterize the network-wide impact of stimulating each region, we set non-significant PLV changes to zero (determined by comparing the observed changes against phase-randomized surrogate data; see Sec. SXII) and then compute network-averages of the absolute PLV modulations. Upon examination of the global responses $\langle |\Delta\rho_{\delta_i}^{\text{base}}| \rangle$ and $\langle |\Delta\rho_{\delta_i}^{\text{exc}}| \rangle$ (Figs. D, panels B,D), we again find significant variability across the choice of the perturbed area. Actually, in comparing the overall baseline band changes at WP1 (Fig. **??**B) to the changes at WP2 (Fig. D, panel B), we find that the dispersion (as quantified by the coefficient of variation of the set of changes $\{\langle |\Delta\rho_{\delta_i}^{\text{base}}| \rangle\}$ and the mean of the global responses are both larger at the second working point. We additionally note, however, that the mean response induced in the excited frequency band, $\langle |\Delta\rho^{\text{exc}}| \rangle$, decreases from WP1 to WP2. Hence, when the system operates around the state of peak ρ^{global}, the coherence modulations in the baseline frequency band are larger – on average – and also more heterogeneously distributed relative to the responses at the working point below the state of peak ρ^{global}, while the average of the global responses in the excited band decreases. We consider these points further in the final section of the **??**, where we more generally examine the state-dependence of perturbation-induced changes in phase-locking.

To conclude this section, we consider the relationships between changes in phase-locking induced by stimulating a given region and the structural or functional node strength of the stimulated area. A number of the associations that exist at WP2 (Fig. D, panels E,F) were also observed at WP1 (see Fig. **??**E,F). For example, the structural strength s_i^{struc} remains strongly positively correlated with the mean absolute PLV change induced in the excited frequency band $\langle |\Delta\rho_{\delta_i}^{\text{exc}}| \rangle$ (Fig. D, panel E, Right). Furthermore, functional strength s_i^{func} retains the strongest positive correlation with the absolute change in baseline band coherence $\langle |\Delta\rho_{\delta_i}^{\text{base}}| \rangle$ (Fig. D, panel F, Left). Thus, global effects in the new, excited frequency band continue to be strongly predicted by anatomical connectivity, whereas fluctuations in

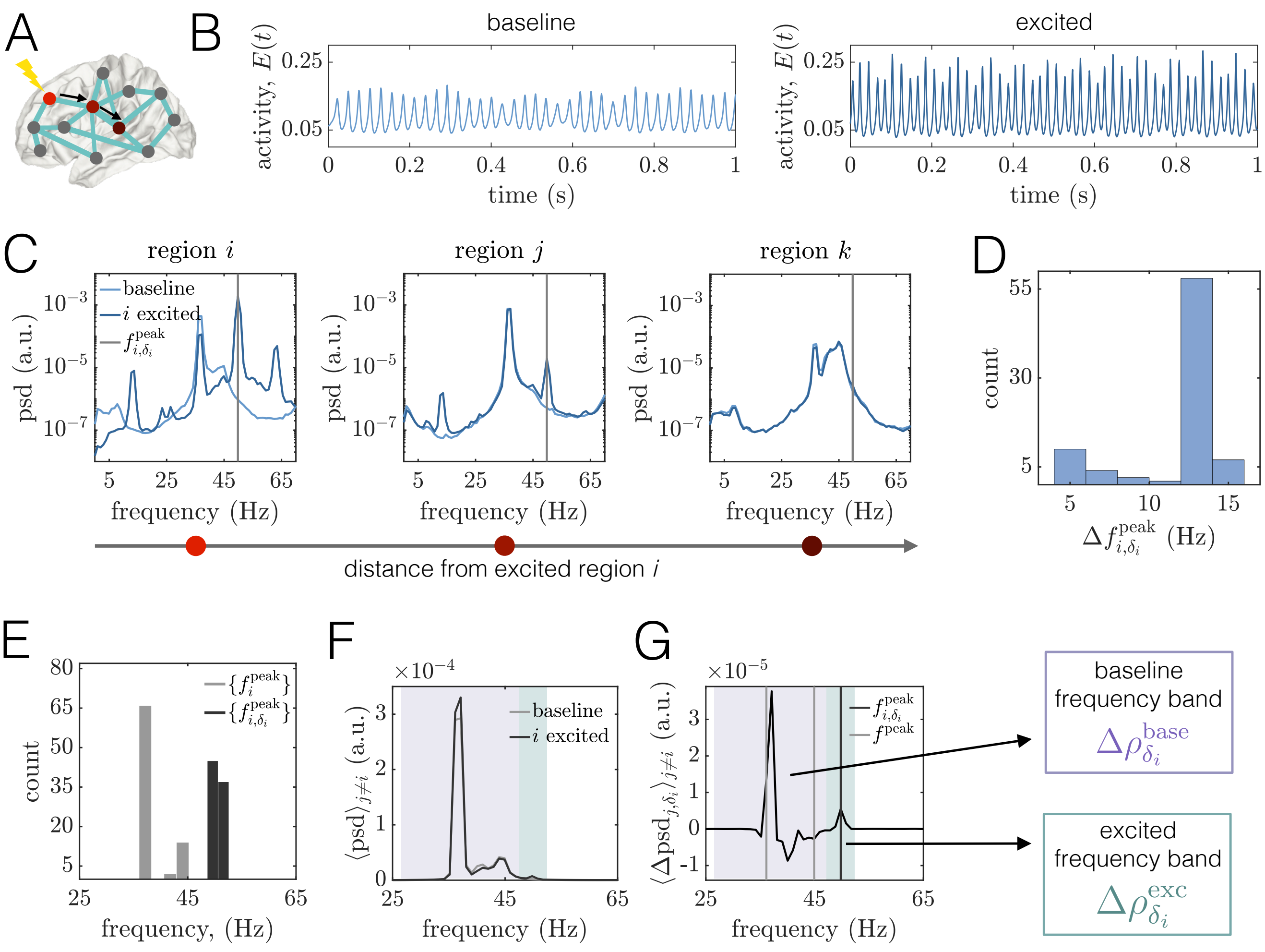

Fig C. Alterations to power spectra induced by focal stimulation at the state near peak baseline coherence (WP2). *(A)* Schematic of a brain network depicting the stimulated site i in brightest red. The black arrows point to two other regions j and k that lie at progressively further topological distances from the perturbed area in the structural network. In this figure, regions i, j, and k correspond to brain areas 1 (R–Lateral Orbitofrontal), 4 (R–Medial Orbitofrontal), and 10 (R–Precentral), respectively. *(B)* Left: A segment of region i's activity time-course in the baseline condition. Right: A segment of region i's activity time-course when it is stimulated. *(C)* Power spectra of area i and two other downstream regions j and k. In all three panels, the lighter curves correspond to the baseline condition, and the darker curves correspond to the state in which i is driven with additional input. The gray vertical lines indicate the peak frequency $f^{\text{peak}}_{i,\delta_i}$ of region i in the excited condition. *(D)* Histogram of the shift in peak frequency $\Delta f^{\text{peak}}_{i,\delta_i}$ induced by stimulating unit i, plotted over all choices of the perturbed area. *(E)* Distribution of peak frequencies of all units in the baseline condition $\{f^{\text{peak}}_i\}$ (light gray) and distribution of the peak frequency units acquire when directly excited $\{f^{\text{peak}}_{i,\delta_i}\}$ (dark gray). *(F)* Average power spectra $\langle \text{psd} \rangle_{j \neq i}$ over all units $j \neq i$ at baseline (light gray) and when unit i is perturbed with additional input (dark gray). *(G)* Average difference $\langle \Delta \text{psd}_{j,\delta_i} \rangle_{j \neq i}$ of the spectra of unit $j \neq i$ when unit i is excited and in the baseline condition, where the average is over all units $j \neq i$. For reference, the light gray vertical lines denote the minimum and maximum peak frequency across units in the baseline state, and the dark gray line indicates the peak frequency acquired by the stimulated region i. Shaded boxes denote two frequency bands of interest: *(1)* the *baseline* band (purple) consisting of the main oscillation frequencies of brain areas under baseline conditions, and *(2)* the *excited* band (green) centered around the peak frequency that the stimulated region inherits. In subsequent analyses, we assess perturbation-induced changes in the PLV between brain areas in the baseline band, $\Delta \rho^{\text{base}}_{\delta_i}$ (purple), and in the excited band $\Delta \rho^{\text{exc}}_{\delta_i}$ (green).

the baseline frequency band continue to be best predicted by the strength of regions' initial coherence with the system as a whole. The main difference between WP1 and WP2 is that for the second working point, a positive correlation

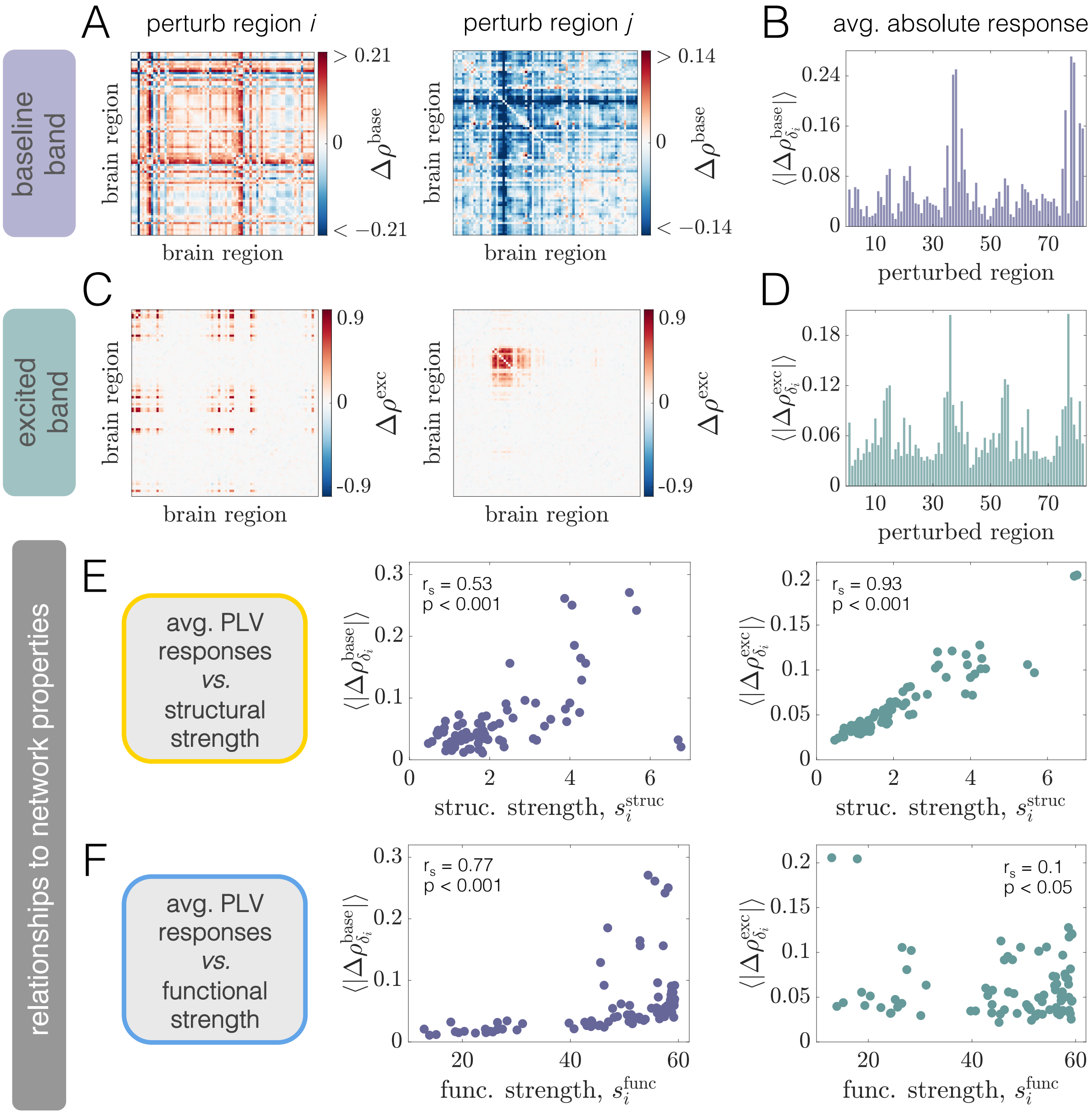

Fig D. Phase-locking changes at WP2 are driven by local excitations of neural activity, differ between excited and baseline frequency bands, and are differentially related to structural and functional network properties. *(A)* Pairwise changes in the PLV inside the baseline band $\Delta\rho^{\text{base}}$ when region i (Left) or region $j \neq i$ (Right) is perturbed. In this figure, regions i and j correspond to regions 4 (R–Medial Orbitofrontal) and 23 (R–Lateral Occipital), respectively. *(B)* Network-averaged absolute PLV changes in the baseline band $\langle|\Delta\rho^{\text{base}}_{\delta_i}|\rangle$ caused by stimulation of different brain areas. *(C)* Pairwise changes in the PLV inside the excited band $\Delta\rho^{\text{exc}}$ when region i (Left) or region $j \neq i$ (Right) is perturbed. *(D)* Network-averaged absolute PLV changes in the excited band $\langle|\Delta\rho^{\text{exc}}_{\delta_i}|\rangle$ induced by stimulation of different brain areas. *(E)* The quantity $\langle|\Delta\rho^{\text{base}}_{\delta_i}|\rangle$ *vs.* structural node strength s_i^{struc} (Left), and the quantity $\langle|\Delta\rho^{\text{exc}}_{\delta_i}|\rangle$ *vs.* structural node strength s_i^{struc} (Right). *(F)* The quantity $\langle|\Delta\rho^{\text{base}}_{\delta_i}|\rangle$ *vs.* functional node strength s_i^{func} (Left), and the quantity $\langle|\Delta\rho^{\text{exc}}_{\delta_i}|\rangle$ *vs.* functional node strength s_i^{func} (Right). In panels *(E)* and *(F)*, insets indicate Spearman correlation coefficients between the plotted quantities and their associated p-values).

also emerges between $\langle|\Delta\rho^{\text{base}}_{\delta_i}|\rangle$ and s_i^{struc} (Fig. D, panel E, Left), indicating that structural connectivity becomes partially indicative of stimulation-induced changes in baseline band phase-locking. In this way, the effects at WP2 have similarities to both WP1 and WP3.

SIV Average excited-band responses differ between stimulation of cortical and subcortical areas

In this section we show that the overall responses induced in the excited frequency band (at WP1 and WP2) are – on average – larger for stimulation of subcortical *vs.* cortical brain areas. To do so, we first compute the average absolute change in excited-band phase-locking $\langle|\Delta\rho^{\text{exc}}_{\delta_i}|\rangle$ for stimulation of each region $i \in \{1, ..., N\}$. Note that for these computations, only statistically significant PLV changes are retained as non-zero (see Sec. SXII). We then collect the sets of responses $\{\langle|\Delta\rho^{\text{exc}}_{\delta_i}|\rangle\}_c$ and $\{\langle|\Delta\rho^{\text{exc}}_{\delta_i}|\rangle\}_s$ corresponding to perturbation of cortical and subcortical areas, respectively. Fig. E shows the mean and spread of these two groups at WP1 (panel A) and at WP2 (panel B), from which we observe that the mean excited-band response is larger for stimulation of subcortical regions at both working points. To determine whether this effect is statistically significant, we perform a non-parametric permutation test of the null hypothesis that there is no difference in the mean excited-band response between cortical and subcortical areas. Using 1000 randomizations, we find that the mean excited-band responses are indeed significantly larger for stimulation of subcortical regions ($p = 0.001$) at both WP1 and WP2.

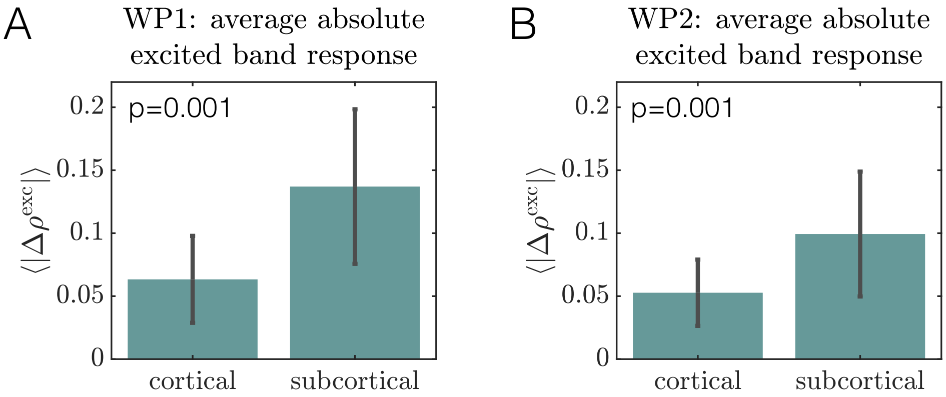

Fig E. Average excited-band responses differ between stimulation of cortical and subcortical areas. *(A)* At WP1, the mean $\langle|\Delta\rho^{\text{exc}}|\rangle$ of the groups $\{\langle\Delta\rho^{\text{exc}}_{\delta_i}|\rangle\}_c$ and $\{\langle|\Delta\rho^{\text{exc}}_{\delta_i}|\rangle\}_s$ corresponding to the average absolute changes in excited-band PLV induced by stimulation of either cortical or subcortical areas, respectively. Error bars indicate ± one standard deviation. The mean subcortical response is significantly greater than the mean cortical response ($p = 0.001$). *(B)* At WP2, the mean $\langle|\Delta\rho^{\text{exc}}|\rangle$ of the sets $\{\langle\Delta\rho^{\text{exc}}_{\delta_i}|\rangle\}_c$ and $\{\langle|\Delta\rho^{\text{exc}}_{\delta_i}|\rangle\}_s$ corresponding to the average absolute changes in excited-band PLV induced by stimulation of either cortical or subcortical areas, respectively. Error bars indicate ± one standard deviation. The mean subcortical response is significantly greater than the mean cortical response ($p = 0.001$).

SV Topological distances in structural network predict power modulations at frequency of stimulated unit

In the main and supplementary text, we show examples of how focal stimulation of one region in the network affects the spectra of downstream areas (see Figs. ??C, ??C, and C, panel C). At WP1 (Fig. ??C) and WP2 (C, panel C), we observed a propagation effect in which a downstream area located at a short topological distance from the perturbed site developed an increase in power at the excited frequency of the directly stimulated area. In constrast, a unit located topologically further from the stimulated area exhibited much weaker power modulations. In this section, we show quantitatively that the topological distance from the perturbed site to downstream regions is a relatively good predictor of the downstream power modulation at the peak frequency of the stimulated unit. Hence, the effects observed in the examples shown in Figs. ??C and C, panel C generalize to other choices of the stimulated unit.

To begin, we more formally define the measure of topological distance that we employ. In general, a topological distance between any two nodes i and j is the length of the shortest path between those nodes, where a path is a non-intersecting sequence of edges that share a common node. Hence, to compute a topological distance, we first need to assign lengths to each edge in the network. Here, we define the length of an edge to be the inverse of the corresponding structural connectivity edge weight [5]. (For this analysis, we use the edge weights from the normalized

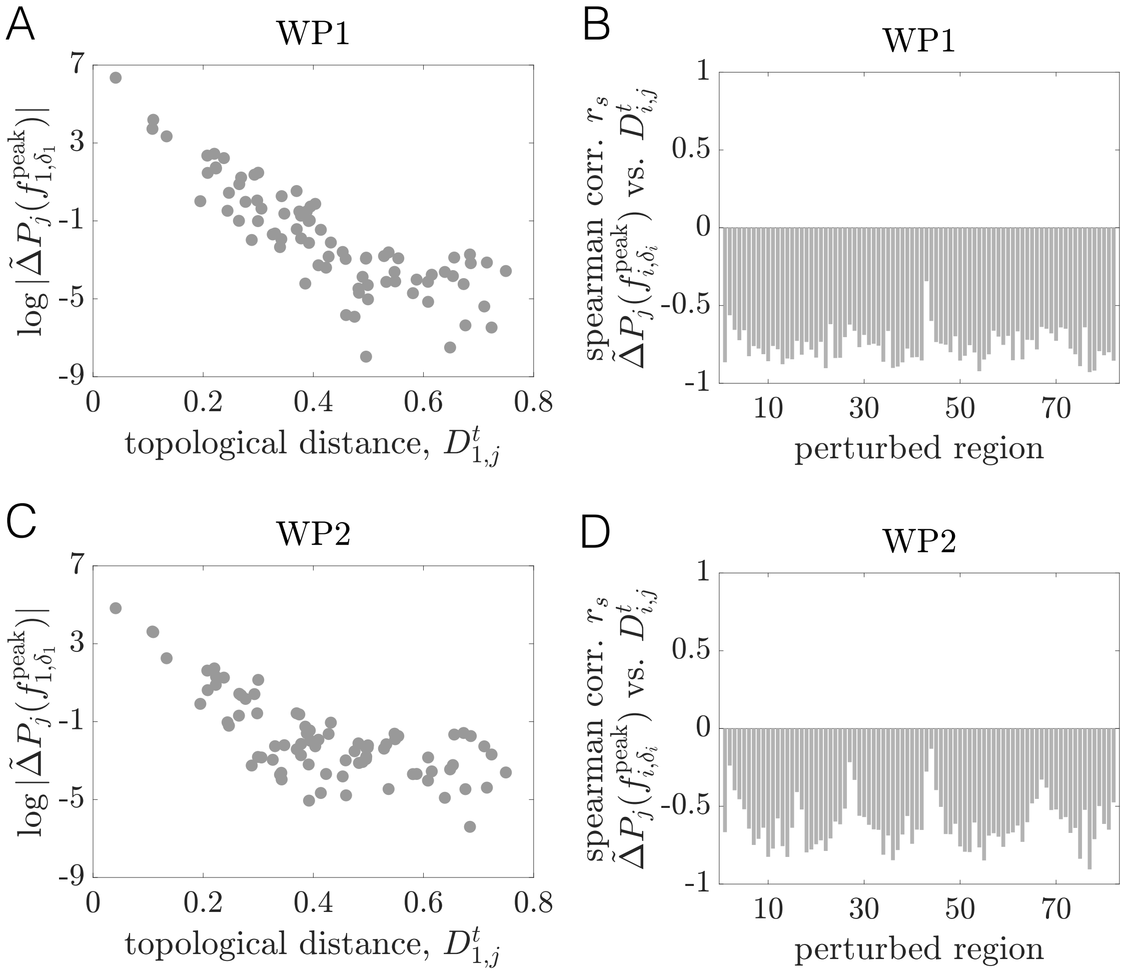

Fig F. Topological distances in structural network predict power modulations at the excited frequency of the stimulated unit. *(A)* WP1: The logarithm of the absolute power modulation $\log|\tilde{\Delta}P_j(f^{\text{peak}}_{1,\delta_1})|$ of downstream regions $j \neq 1$ at the peak frequency of the stimulated area (unit 1) *vs.* the topological distance $D^t_{1,j}$ of unit j from the stimulated site (in the structural network). *(B)* WP1: The Spearman correlation r_s between $\tilde{\Delta}P_j(f^{\text{peak}}_{i,\delta_i})$ and $D^t_{i,j}$ (where $j \neq i$), shown for each choice of the stimulated area i. The correlations are negative and statistically significant in all cases. *(C)* WP2: The logarithm of the absolute power modulation $\log|\tilde{\Delta}P_j(f^{\text{peak}}_{1,\delta_1})|$ of downstream regions $j \neq 1$ at the peak frequency of the stimulated area (unit 1) *vs.* the topological distance $D^t_{1,j}$ of unit j from the stimulated site (in the structural network). *(D)* WP2: The Spearman correlation r_s between $\tilde{\Delta}P_j(f^{\text{peak}}_{i,\delta_i})$ and $D^t_{i,j}$ (where $j \neq i$), shown for each choice of the stimulated area i. The correlations are negative and statistically significant in almost all cases.

adjacency matrix W_{ij}; see **??**). With this definition, the topological distance from node i to node j will thus be shorter when node i links to node j via a path of stronger structural connections.

Now, letting i denote the stimulated region and $j \neq i$ denote a downstream region, we define $D^t_{i,j}$ as the topological distance from node i to node j, and $\tilde{\Delta}P_j(f^{\text{peak}}_{i,\delta_i})$ as the relative change in power of unit j's spectra at the excited peak frequency of the stimulated unit i. In particular, the relative change is computed between baseline and the condition in which i is driven with additional input.

With these quantities defined, we now study their relationship at WP1. Fig. F, panel A shows a plot of $\log|\tilde{\Delta}P_j(f^{\text{peak}}_{1,\delta_1})|$ *vs.* $D^t_{1,j}$ (i.e. we consider the case that the stimulated unit $i = 1$, which was the example in Fig. **??**C.) This scatter plot exhibits a relatively clear negative trend, indicating that units located topologically nearer to the stimulated site (in the structural network) tend to exhibit stronger spectral modulations at the excited peak frequency of the stimulated region, whereas units that are further away (hence more weakly structurally connected) show little change. Furthermore, by examining the Spearman correlation between $\tilde{\Delta}P_j(f^{\text{peak}}_{i,\delta_i})$ and $D^t_{i,j}$ for all choices of the stimulated unit i, we see that this relationship holds more generally, regardless of which site is given the

perturbation (Fig. F, panel B). Specifically, the correlation between the relative power modulation $\tilde{\Delta}P_j(f^{\text{peak}}_{i,\delta_i})$ and the topological distance $D^t_{i,j}$ is consistently negative and statistically significant across all choices of the stimulated area. Fig. F, panel C and Fig. F, panel D illustrate that the same conclusions generally hold for WP2 (although a few correlations lose statistical significance at this working point).

SVI Average responses to perturbations in the baseline frequency band at WP1 vs. WP3

To investigate how the dynamical state of the brain network model influences the effects of focal stimulation, we consider the relationship between the global responses to stimulation at two different working points. In particular, we examine the average absolute change in phase-locking in the baseline frequency band, $\langle|\Delta\rho^{\text{base}}_{\delta_i}|\rangle$, at WP3 *vs.* at WP1 (Fig. G). Recall that WP1 corresponds to a low background drive working point preceding peak baseline coherence, whereas WP3 corresponds to a high background drive working point following peak baseline coherence. It is clear upon visual inspection of Fig. G that there is no consistent relationship between these two quantities. Hence, regions that induce a large response at the system's spontaneous frequencies at WP1 are not necessarily those that induce a large response at WP3.

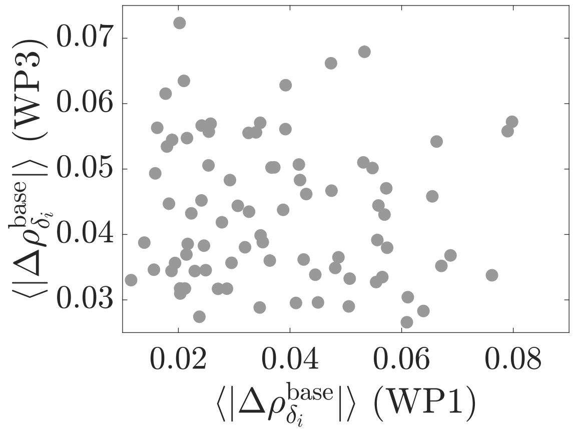

Fig G. Network-averaged absolute change in baseline band phase-coherence $\langle|\Delta\rho^{\text{base}}_{\delta_i}|\rangle$ plotted at WP3 *vs.* WP1. Each point corresponds to a different choice of the stimulated region. Also note that PLV changes that were not statistically significant (see Sec. SXII) were set to zero before computing these network-wide averages.

SVII Negative PLV changes drive correlation with structural connectivity at WP3

In the main text we observed a strong positive correlation between a node's structural strength s^{struc}_i and the absolute coherence modulation $\langle|\Delta\rho^{\text{base}}_{\delta_i}|\rangle$ it induces upon perturbation at WP3 (see Fig. ??). Here, Fig. H demonstrates that this association is largely driven by a strong relationship between s^{struc}_i and the network-averaged absolute decreases $\langle|\downarrow\Delta\rho^{\text{base}}_{\delta_i}|\rangle$ in baseline band coherence induced by the focal stimulation. In particular, s^{struc}_i and $\langle|\downarrow\Delta\rho^{\text{base}}_{\delta_i}|\rangle$ have a Spearman correlation of $r_s = 0.72$, which is almost as strong as the correlation between structural strength s^{struc}_i and the absolute change $\langle|\downarrow\Delta\rho^{\text{base}}_{\delta_i}|\rangle$ ($r_s = 0.82$).

SVIII Verification of relationships between phase-locking modulations and structural or functional connectivity at alternate working points in the low, medium, and high background drive regimes

At WP1, WP2, and WP3, we considered the associations between the average changes in phase-locking induced by regional perturbations (within both the baseline and excited frequency bands) and structural or functional network properties of the stimulated region. We found that, depending on the baseline state of the system, different

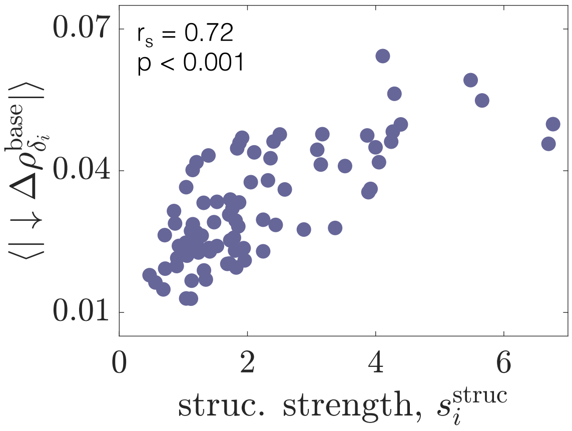

Fig H. The global decrease in baseline band PLV at the high-drive working point (WP3) strongly correlates with the structural strength of the stimulated region. Scatter plot showing the structural strength s_i^{struc} of the stimulated area *vs.* the absolute value of the network-averaged decrease in baseline band coherence $\langle | \downarrow \Delta\rho_{\delta_i}^{\text{base}} | \rangle$. The inset gives the corresonding Spearman correlation and p-value. Also note that only the statistically significant PLV changes (see Sec. SXII) are counted as non-zero when computing the network-wide average.

relationships emerged between the perturbation-induced responses and structural or functional node strengths (see Figs. **??**, D, and **??**). In this section, we verify that qualitatively similar relationships hold for other working points in the immediate vicinity of those studied in the main text. Note that for each alternative working point, we consider the same excitation strength used originally (i.e., $\Delta P_{E,i} = 0.1$). Moreover, throughout this section, we set non-significant PLV changes to zero (determined by comparing the observed changes against phase-randomized surrogate data; see Sec. SXII) prior to computing network-averages of the absolute PLV modulations.

We begin by analyzing an alternative working point near WP1, which we term WP1_{alt}. WP1 was located at $P_E^{\text{base}} = 0.553$ and $C = 2.5$; for WP1_{alt}, we consider parameters $P_E^{\text{base}} = 0.555$ and $C = 2.5$. Note that because peak global baseline coherence ρ^{global} is reached rapidly as a function of P_E^{base} (see Fig. **??**A), in order to consider a second working point located prior to ρ^{global} but still near WP1, we can only shift P_E^{base} slightly from its value at WP1. We find the same set of relationships between phase-locking modulations and structural or functional strength at WP1_{alt} as we did at WP1 (see Fig. I, panels A,D). Specifically: *(1)* the average absolute PLV change induced in the excited frequency band $\langle |\Delta\rho_{\delta_i}^{\text{exc}}| \rangle$ is most strongly associated with the structural strength of the perturbed region s_i^{struc} (Fig. I, panel A,Bottom), and *(2)* the average absolute phase-locking modulation in the baseline frequency band $\langle |\Delta\rho_{\delta_i}^{\text{base}}| \rangle$ is most strongly associated with the functional strength of the perturbed region s_i^{func} (Fig. I, panel D,Top). The relationships between $\langle |\Delta\rho_{\delta_i}^{\text{base}}| \rangle$ and s_i^{struc}, and between $\langle |\Delta\rho_{\delta_i}^{\text{exc}}| \rangle$ and s_i^{func} are either not statistically significant or weaker, respectively (Fig. I, panel A,Top and Fig. I, panel D,Bottom).

We next analyze an alternative working point near WP2, WP2_{alt}. Recall that WP2 was located at $P_E^{\text{base}} = 0.57$ and $C = 2.5$; for WP2_{alt}, we consider $P_E^{\text{base}} = 0.572$ and $C = 2.5$. In order to examine a second working point in close vicinity of the peak in global baseline coherence – which was the condition used to determine parameters for WP2 – we again must consider only a small change in P_E^{base} away from its value at WP2. As before, this is because the dynamical state of the system changes quickly as a function of P_E^{base} in this regime (see Fig. **??**A). Using the specified parameter choices, we find consistent relationships at WP2 and WP2_{alt} in terms of how perturbation-induced phase-locking modulations are related to structural and functional node strength. First, the average absolute PLV changes that arise in the baseline frequency band $\langle |\Delta\rho_{\delta_i}^{\text{base}}| \rangle$ are significantly correlated with the structural strength of the perturbed region s_i^{struc} (Fig. I, panel B,Top), but remain most strongly related to functional strength s_i^{func} (Fig. I, panel E,Top). Second, the average absolute phase-locking modulations in the excited frequency band $\langle |\Delta\rho_{\delta_i}^{\text{exc}}| \rangle$ are strongly associated with the structural strength s_i^{struc} of the perturbed region (Fig. I, panel B,Bottom), and are not significantly correlated with functional strength (Fig. I, panel E,Bottom).

Lastly, we analyze an alternative working point near WP3, WP3_{alt}. WP3 was located at $P_E^{\text{base}} = 0.7$ and $C = 2.5$; for WP3_{alt}, we consider $P_E^{\text{base}} = 0.68$ and $C = 2.5$. We once more find that the relationships between phase-locking modulations induced by regional stimulation and structural or functional node strength are consistent across WP3 and WP3_{alt} (see Fig. I, panel C,F). In particular, there is a strong positive correlation between the average absolute phase-locking modulations induced in the baseline frequency band $\langle |\Delta\rho_{\delta_i}^{\text{base}}| \rangle$ and the structural strength of the perturbed region s_i^{struc} (Fig. I, panel C,Top). The relationship between $\langle |\Delta\rho_{\delta_i}^{\text{base}}| \rangle$ and functional strength s_i^{func} is

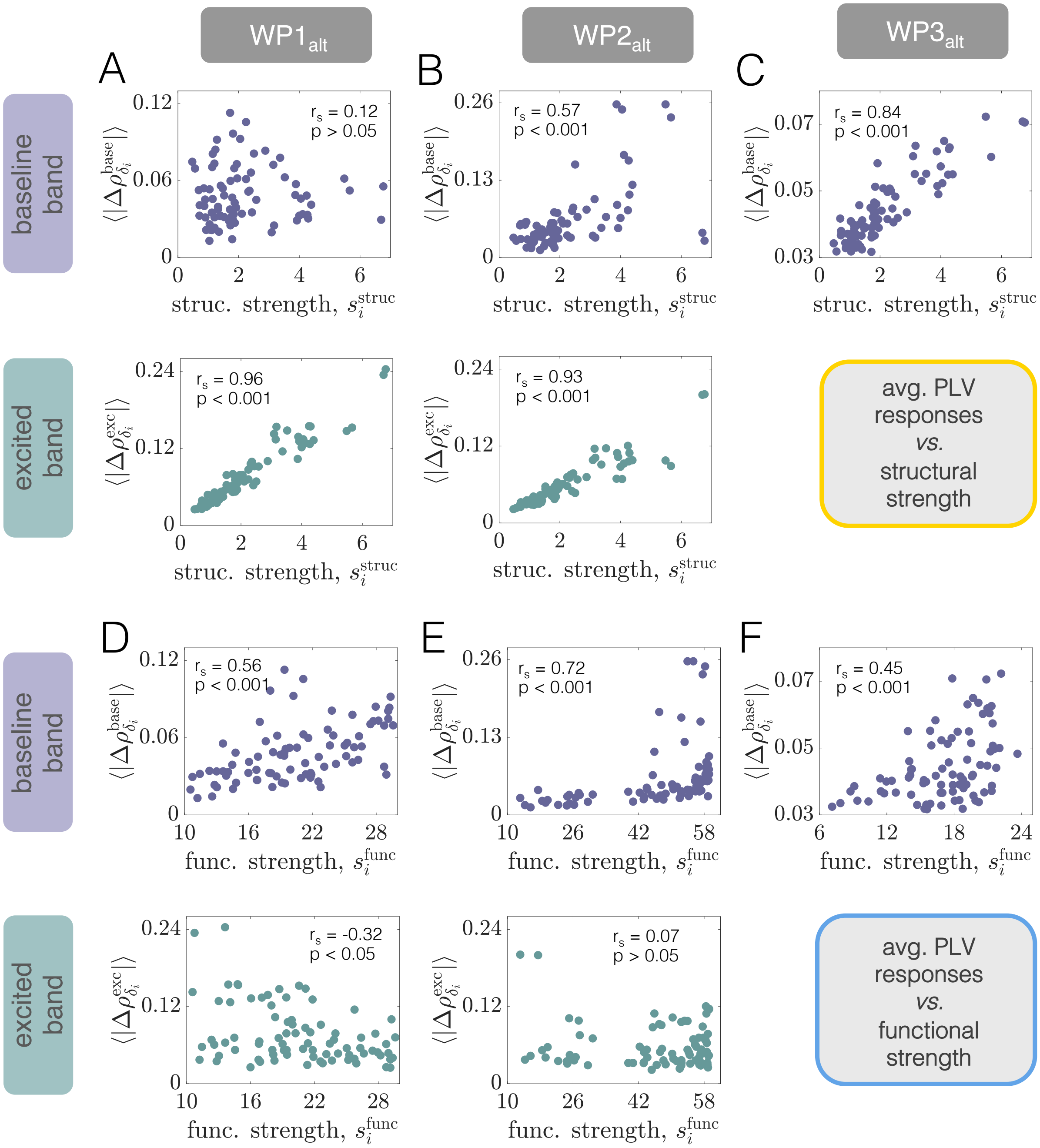

Fig I. Associations between stimulation-induced modulations in phase-locking and structural or functional connectivity hold for alternative working points near WP1, WP2, and WP3. In all panels, $\langle|\Delta\rho_{\delta_i}^{\mathrm{base}}|\rangle$ and $\langle|\Delta\rho_{\delta_i}^{\mathrm{exc}}|\rangle$ correspond to the average absolute PLV changes that arise in the baseline or excited frequency bands, respectively, due to perturbation of unit i. Furthermore, s_i^{struc} and s_i^{func} are the structural and functional node strengths of region i. *(A)* At WP1$_{\mathrm{alt}}$, the quantity $\langle|\Delta\rho_{\delta_i}^{\mathrm{base}}|\rangle$ *vs.* s_i^{struc} (Top) and the quantity $\langle|\Delta\rho_{\delta_i}^{\mathrm{exc}}|\rangle$ *vs.* s_i^{struc} (Bottom). *(B)* At WP2$_{\mathrm{alt}}$, the quantity $\langle|\Delta\rho_{\delta_i}^{\mathrm{base}}|\rangle$ *vs.* s_i^{struc} (Top) and the quantity $\langle|\Delta\rho_{\delta_i}^{\mathrm{exc}}|\rangle$ *vs.* s_i^{struc} (Bottom). *(C)* At WP3$_{\mathrm{alt}}$, the quantity $\langle|\Delta\rho_{\delta_i}^{\mathrm{base}}|\rangle$ *vs.* s_i^{struc} (Top). For this working point, there is no excited frequency band. *(D)* At WP1$_{\mathrm{alt}}$, the quantity $\langle|\Delta\rho_{\delta_i}^{\mathrm{base}}|\rangle$ *vs.* s_i^{func} (Top) and the quantity $\langle|\Delta\rho_{\delta_i}^{\mathrm{exc}}|\rangle$ *vs.* s_i^{func} (Bottom). *(E)* At WP2$_{\mathrm{alt}}$, the quantity $\langle|\Delta\rho_{\delta_i}^{\mathrm{base}}|\rangle$ *vs.* s_i^{func} (Top) and the quantity $\langle|\Delta\rho_{\delta_i}^{\mathrm{exc}}|\rangle$ *vs.* s_i^{func} (Bottom). *(F)* At WP3$_{\mathrm{alt}}$, the quantity $\langle|\Delta\rho_{\delta_i}^{\mathrm{base}}|\rangle$ *vs.* s_j^{func} (Top). For this working point, there is no excited frequency band. In all panels, insets indicate Spearman correlation coefficients between the plotted quantities and their associated p-values.

weaker (Fig. I, panel F,Top). Finally, note that there is no excited frequency band at WP3 or $WP3_{alt}$.

SIX General dependence of associations between phase-locking modulations and structural or functional connectivity as a function of background drive

In Fig. 9A of the main text, we showed the difference Δr_s in the strength of the correlation between the average absolute baseline band PLV changes $\langle|\Delta\rho^{\text{base}}_{\delta_i}|\rangle$ and structural (s^{struc}_i) or functional (s^{func}_i) node strength, plotted as a function of the background drive $P^{\text{base}}_E - P^*_E$ for a coupling $C = 2.5$. Here, we additionally show plots of the individual Spearman correlations for $\langle|\Delta\rho^{\text{base}}_{\delta_i}|\rangle$ *vs.* s^{struc}_i and for $\langle|\Delta\rho^{\text{base}}_{\delta_i}|\rangle$ *vs.* s^{func}_i (Fig. J, panel A). Although the precise values of the correlations can vary in a somewhat complex manner as a function of the baseline input, we reiterate the key point that functional strength is more strongly related to baseline band coherence modulations in the low- and medium-drive regimes, whereas structural strength dominates in the high-drive regime.

We also examine the relationships between structural or functional node strength and the network-averaged absolute change $\langle|\Delta\rho^{\text{exc}}_{\delta_i}|\rangle$ in excited-band PLV induced by focal perturbations. Because stimulation fails to induce an excited band when the background drive is too high, we compute Spearman correlations between these quantities only for values of the drive where at least half of the regions yield an excited band upon perturbation. Furthermore, at each working point, correlations are computed only between regions that induce an excited band. We find that areas' structural node strength robustly predicts the global response in the excited band (see Fig. J, panel B). In particular, the Spearman correlation coefficient r_s between s^{struc}_i and $\langle|\Delta\rho^{\text{exc}}_{\delta_i}|\rangle$ is greater than 0.89 across all baseline inputs for which at least half of the units in the network induce excited frequency bands. Furthermore, the strength of the correlation with structural connectivity is consistently much higher than the strength of the correlation using functional connectivity (compare yellow and blue curves in Fig. J, panel B). These results are consistent with the conclusions drawn in the main text positing that network structure mediates the excited-band effects.

SX Effects of perturbation strength

In the main text we studied a single perturbation strength of $\Delta P_{E,i} = 0.1$. In this section, we assess the dependence of various results on the level of additional excitatory input $\Delta P_{E,i}$ received by the stimulated unit (Fig. K). In particular, for both WP1 and WP3, we vary $\Delta P_{E,i}$ between 0.01 and 0.15 in steps of 0.02.

We first analyze how the perturbation strength affects the shift in the peak frequency of the stimulated area. As a summary measure, we consider the change in peak frequency averaged across all choices of the stimulated region, $\langle\Delta f^{\text{peak}}_{i,\delta_i}\rangle$. As expected, this quantity increases with increasing perturbation strength for both WP1 (Fig. K, panel A) and WP3 (Fig. K, panel B). We next study the stimulation-induced changes in phase-locking in the baseline frequency band as a function of the stimulation strength. In particular, we examine the global response (grand average) $\langle|\Delta\rho^{\text{base}}|\rangle$, where the mean change is computed first over all pairs of brain areas for a given stimulation site, and then across all choices of the perturbed region. For both working points, this measure also increases monotonically as a function of $\Delta P_{E,i}$ (Fig. K, panels C,D). Hence, as the strength of the stimulation increases, so does the overall amount of functional reconfiguration at the system's baseline frequencies. For WP1, we find that the grand average PLV change $\langle|\Delta\rho^{\text{exc}}|\rangle$ in the excited band also grows as a function of the perturbation strength (Fig. K, panel E). Note, however, that at WP3 (Fig. K, panel F), no units generate an excited frequency band for $\Delta P_{E,i} < 0.15$, and for $\Delta P_{\text{E},i} = 0.15$, only about 10% of units do so.

In the main text, we found that at WP1, the absolute change in baseline band phase-coherence induced by stimulating region i, $\langle|\Delta\rho^{\text{base}}_{\delta_i}|\rangle$, was strongly correlated with the functional strength of region i, s^{func}_i (Fig. **??**F, Left). In contrast, there was not a strong association between $\langle|\Delta\rho^{\text{base}}_{\delta_i}|\rangle$ and the structural strength s^{struc}_i at WP1 (Fig. **??**E, Left). Here, we observe that the nature of these two relationships remains qualitatively the same across the considered range of stimulation strengths $\Delta P_{E,i}$ (Fig. K, panel G). A second result from the main text was a strong positive correlation between the PLV change induced in the excited frequency band $\langle|\Delta\rho^{\text{exc}}_{\delta_i}|\rangle$, at WP1, and the structural strength s^{struc}_i of the stimulated unit (Fig. **??**E, Right). The present analysis reveals that the relationship between $\langle|\Delta\rho^{\text{exc}}_{\delta_i}|\rangle$ and s^{struc}_i holds across a range of perturbation strengths $\Delta P_{E,i} > 0.05$ (Fig. K, panel I), for which at least half of the units in the network yield an excited frequency band. Note that for $\Delta P_{E,i} < 0.05$, local excitations do not induce an excited frequency band at all (so the correlations are undefined) and for $\Delta P_{E,i} = 0.05$, fewer than half of the units generate excited frequency bands (so we do not consider correlations with structural or functional strength). Finally, for the case of $\Delta P_{E,i} = 0.1$ studied in the main text, there was a strong positive correlation between $\langle|\Delta\rho^{\text{base}}_{\delta_i}|\rangle$ and s^{struc}_i at WP3 (Fig. **??**C, Left) and a weaker positive correlation between $\langle|\Delta\rho^{\text{base}}_{\delta_i}|\rangle$ and s^{func}_i

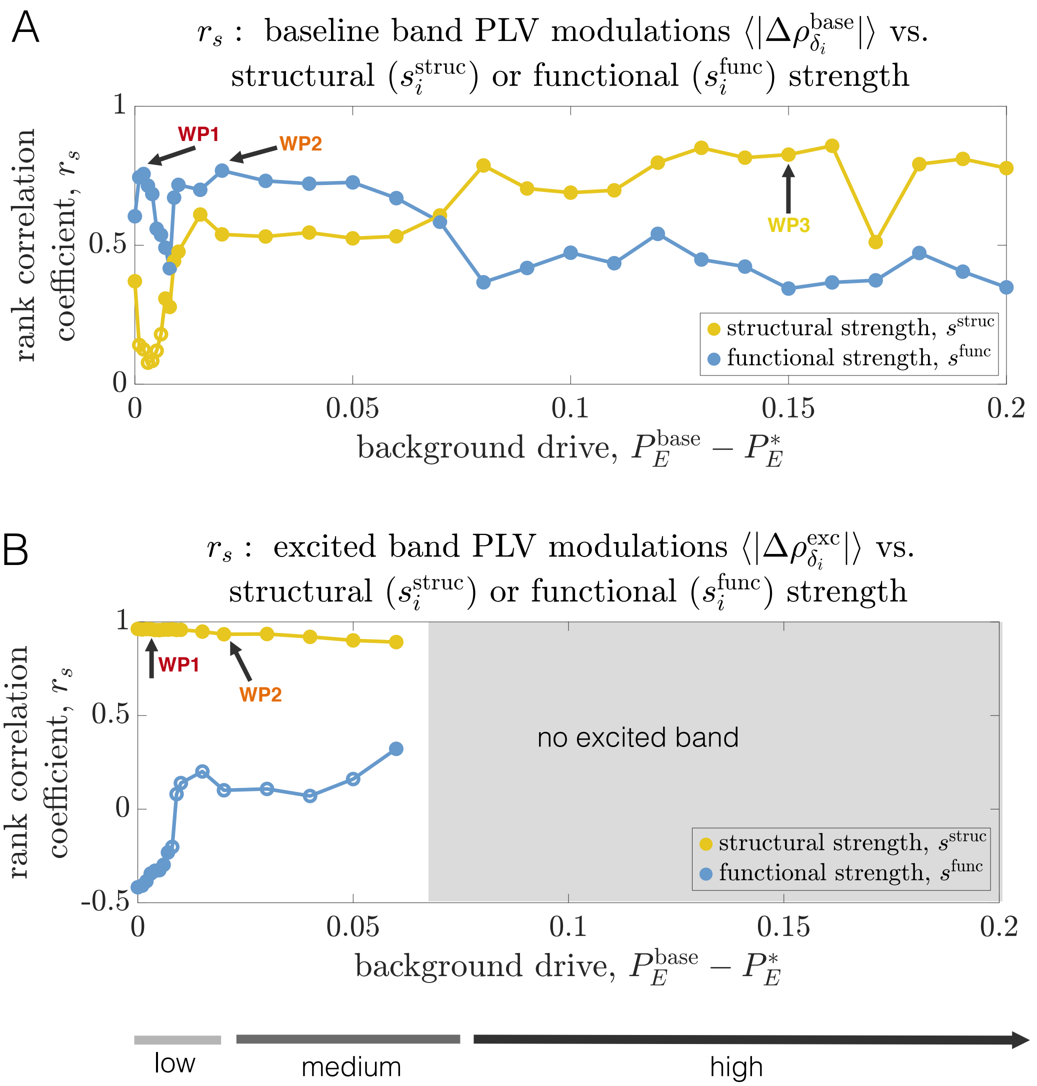

Fig J. Correlations between coherence modulations induced in the baseline band and structural or functional node strength depend on system working point. *(A)* The y-axis shows the Spearman's rank correlation coefficient r_s between network measures of node strength and the average absolute change in baseline band phase-coherence $\langle|\Delta\rho^{\text{base}}_{\delta_i}|\rangle$ induced by stimulation of a single region. The x-axis is the level of background drive $P^{\text{base}}_E - P^*_E$ (for a coupling $C = 2.5$). The yellow curve shows the correlation between structural node strength s^{struc}_i and $\langle|\Delta\rho^{\text{base}}_{\delta_i}|\rangle$, whereas the blue curve shows the correlation between functional node strength s^{func}_i and $\langle|\Delta\rho^{\text{base}}_{\delta_i}|\rangle$. The arrows mark the locations of the working points studied in the main text and in the SI Text. Empty circles indicate that the correlation was not siginficant at the $p = 0.05$ level. *(B)* The y-axis shows the Spearman's rank correlation coefficient r_s between network measures of node strength and the absolute average change in excited band PLV $\langle|\Delta\rho^{\text{exc}}_{\delta_i}|\rangle$ induced by stimulation of a single region. The x-axis is the level of background drive $P^{\text{base}}_E - P^*_E$ (for a coupling $C = 2.5$). The yellow curve shows the correlation between structural node strength s^{struc}_i and $\langle|\Delta\rho^{\text{exc}}_{\delta_i}|\rangle$, whereas the blue curve shows the correlation between functional node strength s^{func}_i and $\langle|\Delta\rho^{\text{exc}}_{\delta_i}|\rangle$. Correlations are computed only between nodes that induce an excited band. The arrows mark the locations of working points studied in the main text and in the SI Text, and the grayed-out portion of the plot denotes baseline inputs at which at least half of the units fail to induce an excited band upon stimulation. Empty circles indicate that the correlation was not siginficant at the $p = 0.05$ level.

(Fig. **??**C, Left). These trends also hold across the range of stimulation strengths examined in this section (Fig. K, panel H). Note that we do not consider correlations between structural and functional node strength and excited band PLV changes, because at WP3, less than half of the network generates an excited frequency band (even for the strongest stimulation strength).

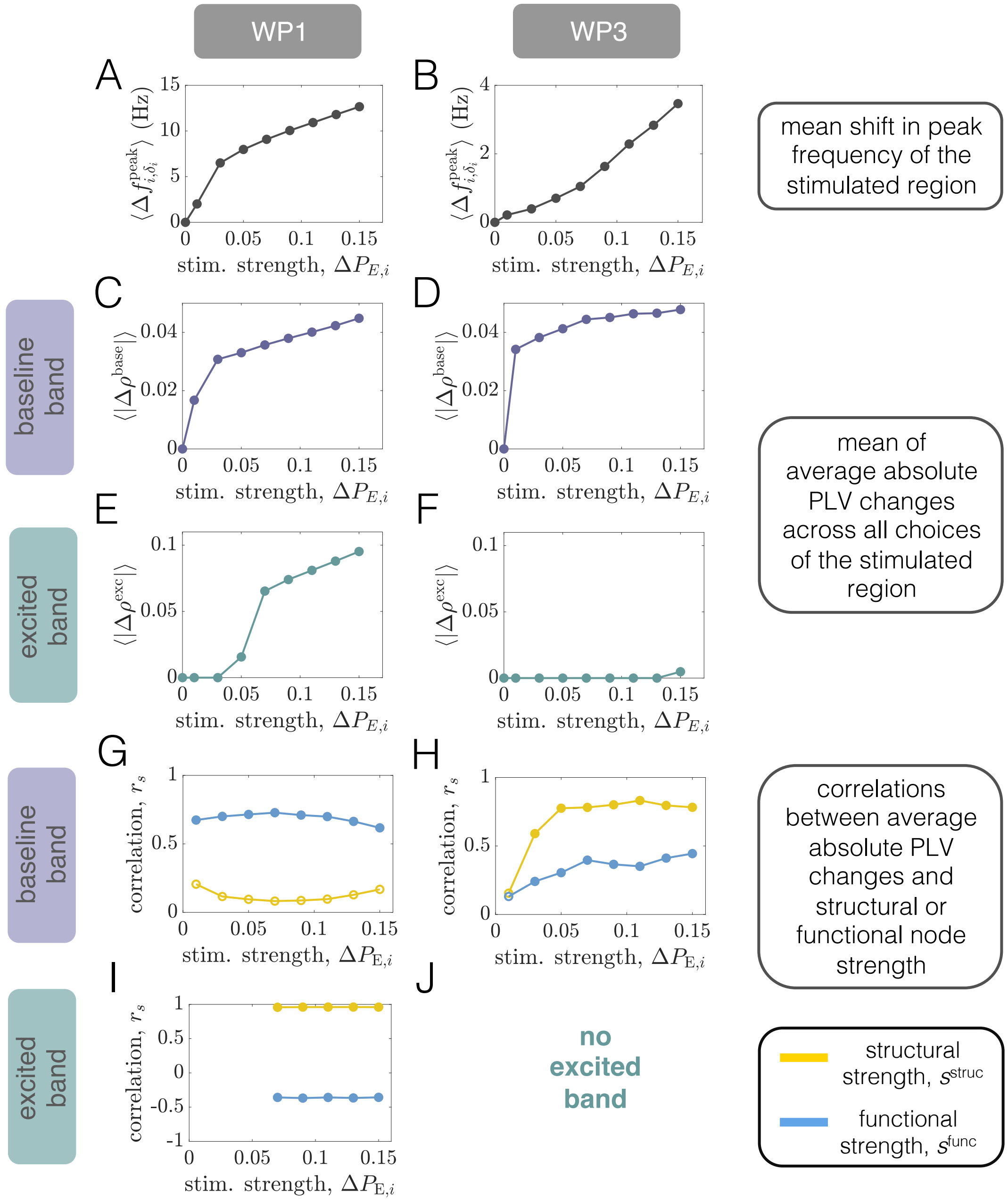

Fig K. Effects of varying the perturbation strength $\Delta P_{E,i}$ at WP1 and WP3. *(A)* The average shift in peak frequency of the stimulated region $\langle \Delta f^{\text{peak}}_{i,\delta_i} \rangle$ *vs.* $\Delta P_{E,i}$ at WP1. *(B)* The average shift in peak frequency of the stimulated region $\langle \Delta f^{\text{peak}}_{i,\delta_i} \rangle$ *vs.* $\Delta P_{E,i}$ at WP3. *(C)* The grand average $\langle |\Delta \rho^{\text{base}}| \rangle$ of the perturbation-induced absolute changes in baseline band PLVs *vs.* $\Delta P_{E,i}$ at WP1. *(D)* The grand average $\langle |\Delta \rho^{\text{base}}| \rangle$ of the perturbation-induced absolute changes in baseline band PLVs *vs.* $\Delta P_{E,i}$ at WP3. *(E)* The grand average $\langle |\Delta \rho^{\text{exc}}| \rangle$ of the perturbation-induced absolute changes in excited band PLVs *vs.* $\Delta P_{E,i}$ at WP1. *(F)* The grand average $\langle |\Delta \rho^{\text{exc}}| \rangle$ of the perturbation-induced absolute changes in excited band PLVs *vs.* $\Delta P_{E,i}$ at WP3. *(G)* At WP1, the Spearman correlation r_s between the network-averaged absolute PLV changes in the baseline band $\langle |\Delta \rho^{\text{base}}_{\delta_i}| \rangle$ and *(1)* structural strength s^{struc}_i (dark gray) or *(2)* functional strength s^{func}_i (light gray), plotted as a function of $\Delta P_{E,i}$. *(H)* At WP3, the Spearman correlation r_s between the network-averaged absolute PLV changes in the baseline band $\langle |\Delta \rho^{\text{base}}_{\delta_i}| \rangle$ and *(1)* structural strength s^{struc}_i (dark gray) or *(2)* functional strength s^{func}_i (light gray), plotted as a function of $\Delta P_{E,i}$. *(I)* At WP1, the Spearman correlation r_s between the network-averaged absolute PLV changes in the excited band $\langle |\Delta \rho^{\text{exc}}_{\delta_i}| \rangle$ and *(1)* structural strength s^{struc}_i (dark gray) or *(2)* functional strength s^{func}_i (light gray), plotted as a function of $\Delta P_{E,i}$. *(J)* Correlations with excited band changes are not considered at WP3 since no or fewer than half of units generate excited bands across all studied values of $\Delta P_{E,i}$. In panels G–I, filled-in circles indicate that the correlation is statistically significant at $p < 0.05$.

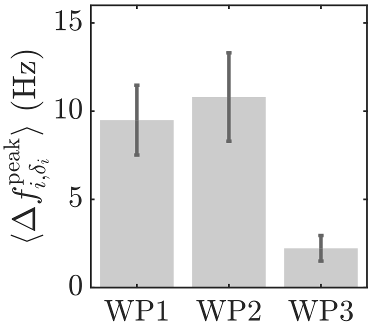

Fig L. Effect of background drive on stimulated regions' power spectra at a coupling of $C = 2$. The average shift in the peak frequency of the perturbed region $\langle \Delta f^{\rm peak}_{i,\delta_i} \rangle$ for WP1, WP2, and WP3 (error bars indicate $\pm$ one standard deviation over all choices of the excited unit).

In conclusion, we note that while an in-depth examination of the effects of the stimulation strength is beyond the scope of the present study, it is important that the main relationships between interareal phase-locking modulations and network properties hold over a range of values for this parameter.

SXI Results for an alternative value of the global coupling

In the main text, we examined the effects of focal excitatory stimulation for a global coupling of $C = 2.5$. Here, we analyze an alternative (but relatively nearby) coupling value of $C = 2.0$, and show that qualitatively similar results are found. As for $C = 2.5$, we consider three different working points by varying the level of background drive $P_E^{\rm base}$, while holding the coupling fixed. Specifically, we consider $P_E^{\rm base} = 0.60$ (WP1), $P_E^{\rm base} = 0.615$ (WP2), and $P_E^{\rm base} = 0.745$ (WP3), which place the system below, at, or above the state of peak global coherence (see Fig. **??**A of the main text), respectively. As before, these working points represent three distinct dynamical states of the system.

We begin by considering the effects of regional stimulation on the power spectra of the perturbed area. To summarize this, we examine the average shift in the peak frequency of the stimulated region, $\langle \Delta f^{\rm peak}_{i,\delta_i} \rangle$, for each of the three working points (Fig. L). For all three states, additional excitation has the effect of increasing the peak frequency of the stimulated region. However, for WP1 and WP2, the peak frequency shifts by a noticeably larger amount ($\langle \Delta f^{\rm peak}_{i,\delta_i} \rangle = 9.5$Hz for WP1 and $\langle \Delta f^{\rm peak}_{i,\delta_i} \rangle = 10.8$Hz for WP2) relative to the more modest effect at WP3 ($\langle \Delta f^{\rm peak}_{i,\delta_i} \rangle = 2.2$Hz). These general trends are consistent with the results in the main text, and again demonstrate that individual areas are most responsive to additional excitation in states of lower background drive (WP1 and WP2). In contrast, given the same excitation strength, regional dynamics are relatively imperturbable when the system operates in the high background drive state (WP3).

We next examine how regional stimulation affects interareal phase-locking at each of the three working points. For WP1 and WP2 we analyze separate "baseline" and "excited" frequency bands, since the peak frequency of the stimulated area becomes separated from the peak frequencies of the system at baseline. For WP3, we consider a single "baseline" band, as the peak frequency of the excited area shifts only slightly and can overlap with the main frequencies at baseline. For the present analysis, we use the same protocol described in the main text to define baseline and excited frequency bands. In general, we refer the reader to the **??** section of the main document for further details and discussion regarding the results presented below.

We first show – for WP1 – examples of the phase-locking modulations within the baseline and excited frequency bands for two different choices of the stimulated area (Figs. M, panels A,B). As in the main text (see Figs. **??**A,C), we see that the network response to a local perturbation differs between the two frequency bands, and for different choices of the stimulated region. We next study the associations between the network-wide average of the phase-locking modulations induced by regional stimulation and structural or functional strength (Figs. M, panels C,D). To characterize the network-wide impact of stimulating each region, we set non-significant PLV changes to zero (determined by comparing the observed changes against phase-randomized surrogate data; see Sec. SXII) and then compute network-averages of the absolute PLV modulations. In comparing the results presented here for a coupling of $C = 2$ to those in Fig. **??**E,F of the primary text for $C = 2.5$, we find similar relations. Specifically, the average absolute change in the PLV for the baseline frequency band $\langle |\Delta\rho^{\rm base}_{\delta_i}| \rangle$ is most strongly related to the baseline functional strength of the stimulated area $s_i^{\rm func}$ (Fig. M, panel D, Left). In contrast, the network-average of the absolute changes in excited band PLV $\langle |\Delta\rho^{\rm exc}_{\delta_i}| \rangle$ is most strongly related to the structural strength of the stimulated region $s_i^{\rm struc}$ (Fig. M, panel C, Right). The other relationships are either weaker or not statistically significant.

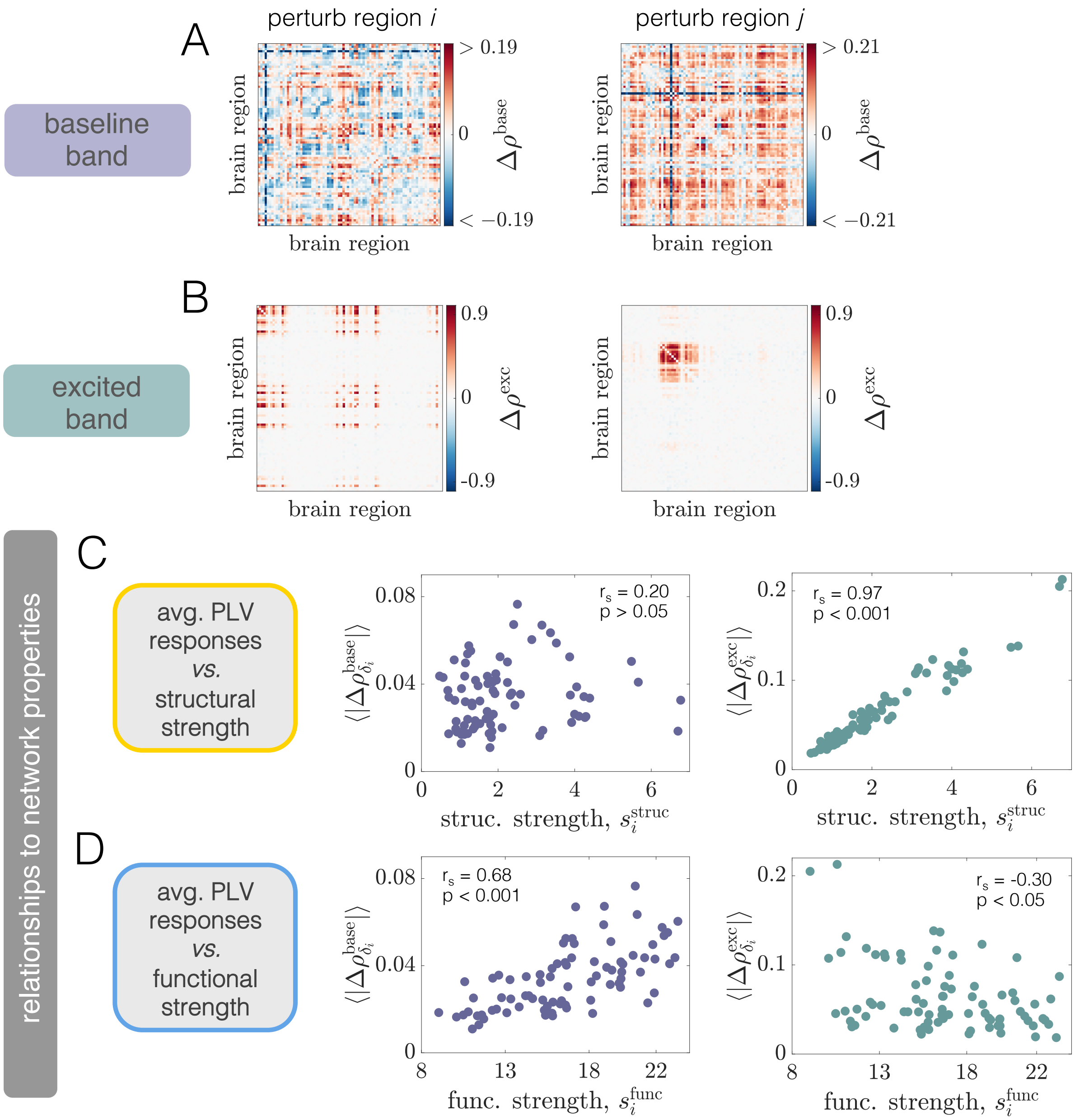

Fig M. Phase-locking modulations induced by regional stimulation at WP1 for a coupling of $C = 2$. *(A)* Pairwise changes in the PLV inside the baseline band $\Delta\rho^{\mathrm{base}}$ when region i (Left) or region $j \neq i$ (Right) is perturbed. In this figure, regions i and j correspond to regions 4 (R–Medial Orbitofrontal) and 23 (R–Lateral Occipital), respectively. *(B)* Pairwise changes in the PLV inside the excited band $\Delta\rho^{\mathrm{exc}}$ when region i (Left) or region $j \neq i$ (Right) is perturbed. *(C)* The quantity $\langle|\Delta\rho^{\mathrm{base}}_{\delta_i}|\rangle$ *vs.* structural node strength s_i^{struc} (Left), and the quantity $\langle|\Delta\rho^{\mathrm{exc}}_{\delta_i}|\rangle$ *vs.* structural node strength s_i^{struc} (Right). *(D)* The quantity $\langle|\Delta\rho^{\mathrm{base}}_{\delta_i}|\rangle$ *vs.* functional node strength s_i^{func} (Left), and the quantity $\langle|\Delta\rho^{\mathrm{exc}}_{\delta_i}|\rangle$ *vs.* functional node strength s_i^{func} (Right). In panels *(C)* and *(D)*, insets indicate Spearman correlation coefficients between the plotted quantities and their associated p-values.

We next conduct the same analyses regarding changes to interareal phase-locking, but for WP2. Here, Figs. N, panels A,B show examples of the phase-locking modulations within the baseline and excited frequency bands for two different choices of the stimulated area, and Figs. N, panels C,D depict relationships between the global PLV changes induced by regional stimulation and structural or functional node strength. We again find qualitatively similar behavior between the results shown here and those depicted in the main text. Note that for both values of the coupling ($C = 2$ here and $C = 2.5$), the main difference between WP1 and WP2 is that structural strength s_i^{struc} also exhibits a positive correlation with the average absolute change in phase-coherence for the baseline frequency band $\langle|\Delta\rho^{\mathrm{base}}_{\delta_i}|\rangle$ (Fig. N, panel C, Left). However, for both values of C, phase-locking modulations at the system's

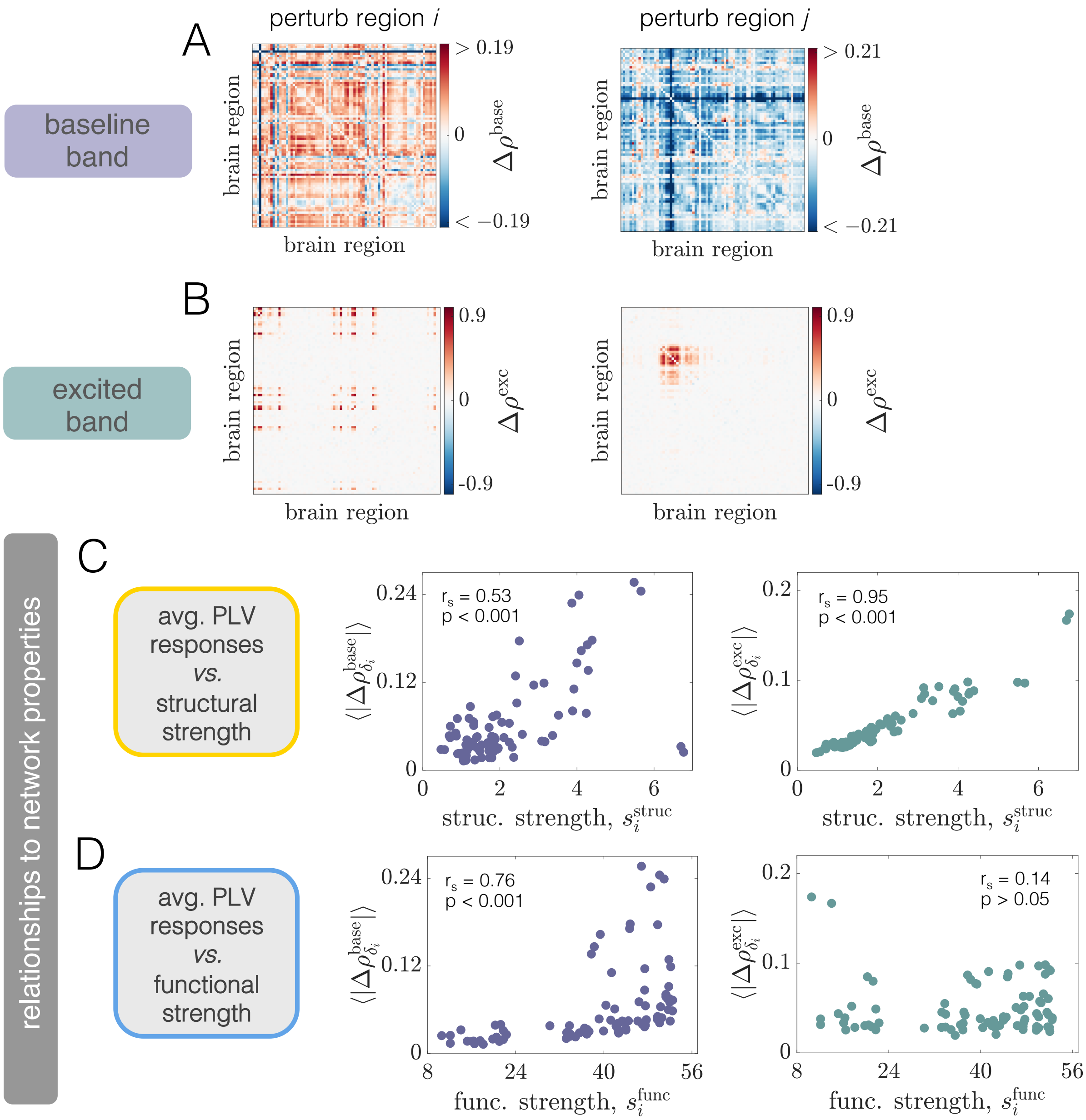

Fig N. Phase-locking modulations induced by regional stimulation for WP2 at a coupling of $C = 2$. *(A)* Pairwise changes in the PLV inside the baseline band $\Delta\rho^{\text{base}}$ when region i (Left) or region $j \neq i$ (Right) is perturbed. In this figure, regions i and j correspond to regions 4 (R–Medial Orbitofrontal) and 23 (R–Lateral Occipital), respectively. *(B)* Pairwise changes in the PLV inside the excited band $\Delta\rho^{\text{exc}}$ when region i (Left) or region $j \neq i$ (Right) is perturbed. *(C)* The quantity $\langle|\Delta\rho^{\text{base}}_{\delta_i}|\rangle$ *vs.* structural node strength s_i^{struc} (Left), and the quantity $\langle|\Delta\rho^{\text{exc}}_{\delta_i}|\rangle$ *vs.* structural node strength s_i^{struc} (Right). *(D)* The quantity $\langle|\Delta\rho^{\text{base}}_{\delta_i}|\rangle$ *vs.* functional node strength s_i^{func} (Left), and the quantity $\langle|\Delta\rho^{\text{exc}}_{\delta_i}|\rangle$ *vs.* functional node strength s_i^{func} (Right). In panels *(C)* and *(D)*, insets indicate Spearman correlation coefficients between the plotted quantities and their associated p-values.

spontaneous frequencies continue to be most strongly associated with the stimulated region's baseline functional strength (Fig. N, panel D, Left).

For completeness, we lastly consider phase-locking changes induced by regional perturbations at WP3. Fig. O, panel A shows the change in PLV between each pair of regions (for the single, baseline frequency band) for two different choices of the stimulated area. As found in the main paper (e.g., Fig. **??**A), the response, in general, differs across the choice of the excited region. The relationships between the phase-coherence modulations and structural or functional network strength found here for $C = 2$ (Fig. O, panel B) are also consistent with the analysis performed in the primary text for $C = 2.5$ (Fig. **??**C). Namely, there is a strong, positive correlation between $\langle|\Delta\rho^{\text{base}}_{\delta_i}|\rangle$ and

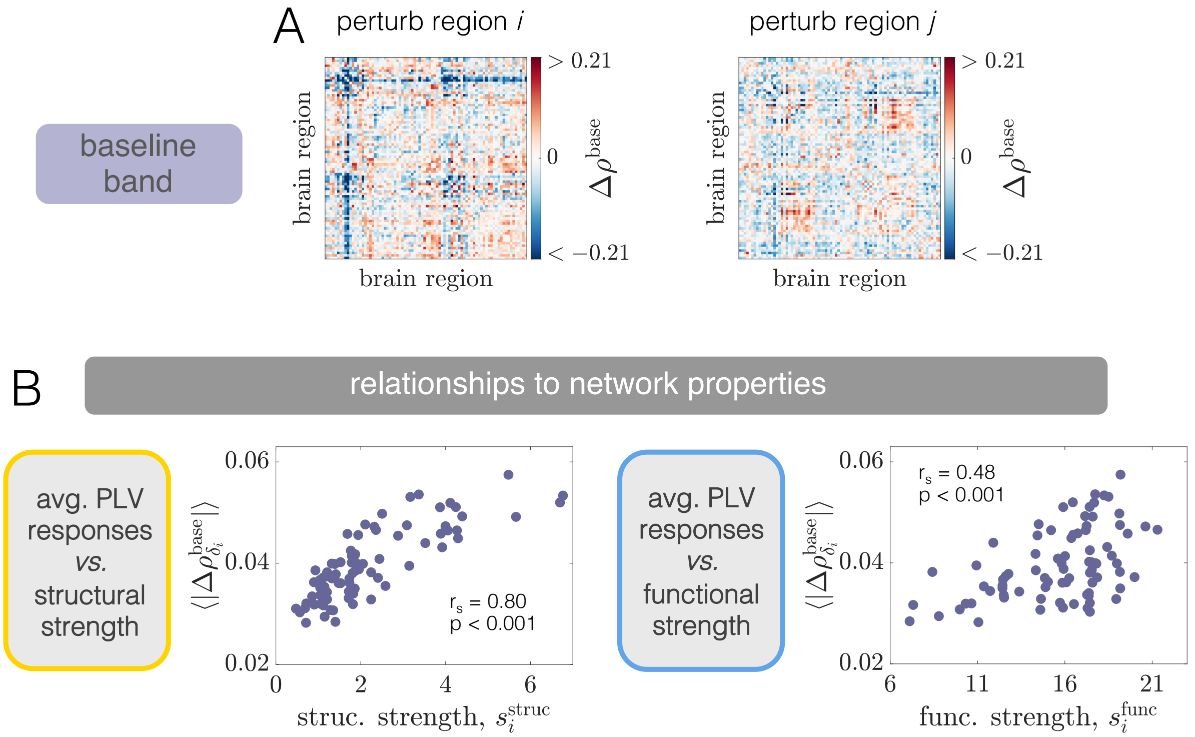

Fig O. Phase-locking modulations induced by regional stimulation for WP2 at a coupling of $C = 2$. *(A)* Pairwise changes in the PLV inside the baseline band $\Delta\rho^{\text{base}}$ when region i (Left) or region $j \neq i$ (Right) is perturbed. Note that in this figure, regions i and i correspond to regions 10 (R–Precentral) and 15 (R–Isthmus), respectively. *(B)* The quantity $\langle|\Delta\rho^{\text{base}}_{\delta_i}|\rangle$ *vs.* structural node strength s_i^{struc} (Left), and the quantity $\langle|\Delta\rho^{\text{base}}_{\delta_i}|\rangle$ *vs.* functional node strength s_i^{func} (Right). Insets indicate Spearman correlation coefficients between the plotted quantities and their associated p-values.

s_i^{struc} and a weaker but still significant correlation between $\langle|\Delta\rho^{\text{base}}_{\delta_i}|\rangle$ and s_i^{func}.

SXII Determining statistical significance of PLV changes using phase-randomized surrogates

Throughout the text, we compute changes in interareal phase-locking between baseline conditions and the case of focal stimulation. In particular, we subtract the PLV matrix computed from baseline simulations from the PLV matrix computed from simulations corresponding to regional stimulation. Of note is that this method does not account for the possibility that observed changes in phase-locking arise only from differences in the autocorrelation structures of units' time-series in the baseline *vs.* stimulation conditions, which could affect results in the case of finite sample sizes. To determine when observed changes in phase-locking are different than the changes expected from a potential change in the autocorrelations of units' dynamics alone, we can conduct statistical significance testing using autocorrelation-preserving surrogate data. We describe this methodology in what follows.

To begin, we will use the terminology $\Delta\rho^{\text{base}}_{jk,\delta_i}$ and $\Delta\rho^{\text{exc}}_{jk,\delta_i}$ to denote the change in baseline band and excited band PLV, respectively, between regions j and k induced by perturbation of unit i. Note that i, j, k are all $\in \{1, ..., N\}$. To determine whether these changes are statistically significant, we need to build null distributions $\{\tilde{\Delta}\rho^{\text{base}}_{jk,\delta_i}\}$ and $\{\tilde{\Delta}\rho^{\text{exc}}_{jk,\delta_i}\}$ against which the observed changes are compared to. Furthermore, we want these null distributions to represent the expected changes in phase-locking for time-series that have the same autocorrelations as those from the baseline- and stimulation- condition simulations, but constructed such that the dependencies between surrogate time-series from different units are destroyed. One well-known autocorrelation-preserving surrogate method is that of phase-randomization [6]. This surrogate method maintains the power spectrum of an original signal $s(t)$, but randomizes its Fourier phases. In short, this is accomplished by computing the discrete Fourier transform of the original signal, adding phases drawn independently and at random from the interval $[0, 2\pi]$ to the phase for each frequency (while preserving the fact that the signal must be real), and then transforming back to the time-

domain to obtain a "phase-randomized" surrogate $\tilde{s}(t)$ [7]. If a different phase-randomization is applied to the surrogates corresponding to different units in the network, then each unit's autocorrelation will be preserved, but interdependencies between different units will be destroyed. In this way, we can test the null hypothesis that the observed PLV changes $\Delta\rho^{\mathrm{base}}_{jk,\delta_i}$ or $\Delta\rho^{\mathrm{exc}}_{jk,\delta_i}$ are due only to finite sample size bias and the differing autocorrelation structures of units' time-series between the baseline and stimulation simulations. If we can reject this null hypothesis, then we conclude that the observed modulations in phase-locking reflect actual changes in interareal coherence, beyond what is expected due to differing autocorrelations.

To generate the null distributions $\{\tilde{\Delta}\rho^{\mathrm{base}}_{jk,\delta_i}\}$ and $\{\tilde{\Delta}\rho^{\mathrm{exc}}_{jk,\delta_i}\}$, we follow the same steps used to generate the true changes $\Delta\rho^{\mathrm{base}}_{jk,\delta_i}$ or $\Delta\rho^{\mathrm{exc}}_{jk,\delta_i}$, with one exception: instead of using units' actual excitatory time-series in the PLV calculations, we use corresponding phase-randomized surrogates. In particular, to generate one instance of $\{\tilde{\Delta}\rho^{\mathrm{base}}_{jk,\delta_i}\}$ or $\{\tilde{\Delta}\rho^{\mathrm{exc}}_{jk,\delta_i}\}$, we generate a phase-randomized surrogate from each unit's activity in every trial, using different randomizations for all units and trials. Then, using the surrogate time-series, we compute one instance of $\tilde{\Delta}\rho^{\mathrm{base}}_{jk,\delta_i}$ and $\tilde{\Delta}\rho^{\mathrm{base}}_{jk,\delta_i}$ following the same steps used to compute the original PLV changes in the baseline and excited frequency bands (see the **??** section of the main text). This process is then repeated 50 times – each time using a different set of surrogate realizations – to generate null distributions $\{\tilde{\Delta}\rho^{\mathrm{base}}_{jk,\delta_i}\}$ and $\{\tilde{\Delta}\rho^{\mathrm{exc}}_{jk,\delta_i}\}$.

Once we have generated null distributions $\{\tilde{\Delta}\rho^{\mathrm{base}}_{jk,\delta_i}\}$ and $\{\tilde{\Delta}\rho^{\mathrm{exc}}_{jk,\delta_i}\}$ for a given choice of the excited unit i, the next step is to compare them to the observed differences $\Delta\rho^{\mathrm{base}}_{jk,\delta_i}$ and $\Delta\rho^{\mathrm{exc}}_{jk,\delta_i}$. To determine if an observed $\Delta\rho_{jk}$ is statistically different from a null distribution $\{\tilde{\Delta}\rho_{jk}\}$, we first check if $\Delta\rho_{jk}$ is positive or negative. If $\Delta\rho_{jk} > 0$, then we compute the fraction of surrogates p_+ for which $\tilde{\Delta}\rho_{jk} > \Delta\rho_{jk}$. If $p_+ < 0.05$, then we conclude that $\Delta\rho_{jk}$ is statistically greater than expected under the null distribution, and the increase in phase-locking is significant. If $\Delta\rho_{jk} < 0$, then we compute the fraction of surrogates p_- for which $\tilde{\Delta}\rho_{jk} < \Delta\rho_{jk}$. If $p_- < 0.05$, then we conclude that $\Delta\rho_{jk}$ is statistically less than expected under the null distribution, and the decrease in phase-locking is significant. If the change in phase-locking $\Delta\rho_{jk}$ is not found to be statistically different from the null distribution, then we set its value to zero prior to computing network-averaged changes in the PLV. Using this procedure, we can check the statistical significance of all PLV changes in the baseline and excited frequency bands, and for each choice of the stimulated node.

The statistical significance testing described above is performed for WP1, WP2, and WP3 in the analyses where we consider network-averaged absolute modulations in phase-locking and their relationships to the structural or functional strength of the perturbed node (e.g., Fig. **??**B,D–F, Fig. D, panels B–F, and Fig. **??**B,C). In particular, at these working points, PLV changes that are not significant (relative to the phase-randomized null model) are set to zero before computing the network-wide averages of PLV modulations in the baseline $\langle|\Delta\rho^{\mathrm{base}}_{\delta_i}|\rangle$ and excited $\langle|\Delta\rho^{\mathrm{exc}}_{\delta_i}|\rangle$ frequency bands. We do not repeat these comparisons against surrogate data for the parameter sweeps over the background drive and stimulation strength. However, findings from WP1, WP2, and WP3 (as well as $\mathrm{WP1_{alt}}$, $\mathrm{WP2_{alt}}$, and $\mathrm{WP3_{alt}}$) indicate that results are largely unaffected by whether or not one sets non-significant PLV changes to zero prior to computing the global response. Throughout the text, we indicate when the statistical significance testing was performed on the PLV changes.

SXIII Details on the Hilbert Transform

A common way to extract an instantaneous phase variable from a real-valued, oscillatory signal is with the Hilbert transform. To begin, one writes the analytic (complex-valued) signal representation $X_{\mathrm{A}}(t)$ of the real-valued time-series $X(t)$ as

$$X_{\mathrm{A}}(t) = X(t) + iX_{\mathrm{H}}(t) = A(t)e^{i\theta(t)}, \tag{1}$$

where $X_{\mathrm{H}}(t)$ is the Hilbert transform of $X(t)$, $A(t)$ is the instantaneous amplitude of $X(t)$, and $\theta(t)$ is the instantaneous phase of $X(t)$. Once one has computed $X_{\mathrm{H}}(t)$ and thus $X_{\mathrm{A}}(t)$, it is apparent from Eq. 1 that the phase $\theta(t)$ can be computed as

$$\theta(t) = \arg[X_{\mathrm{A}}(t)]. \tag{2}$$

The Hilbert transform of a signal $X(t)$ is defined as

$$X_{\mathrm{H}}(t) = \frac{1}{\pi}\int_{-\infty}^{\infty}\frac{X(t')}{t-t'}dt', \tag{3}$$

where the integral is evaluated as a Cauchy principal value. From Eq. 3, one observes that the Hilbert transform is the convolution of $X(t)$ and $1/\pi t$: $X_{\mathrm{H}}(t) = X(t) * 1/\pi t$, so the Fourier transform (FT) of $X_{\mathrm{H}}(t)$, $\tilde{X}_{\mathrm{H}}(f)$, is just the product of the FTs of $X(t)$ and $1/\pi t$. For frequencies $f > 0$, we thus have that $\tilde{X}_{\mathrm{H}}(f) = -i\tilde{X}(f)$, from which it becomes clear that the Hilbert transform just induces a phase shift of $\pi/2$ to each frequency component in the signal.

In this study, we computed Hilbert transforms of the simulated neural activity using the 'hilbert' function in MATLAB. As described in the **??** section, the Hilbert Transform was applied after first filtering the raw time-series in a specified frequency band, in order to ensure that the corresponding phase variable is well-defined [8].

SXIV Brain region identification numbers and labels

Table A. Brain region ID numbers with their corresponding hemisphere (L = left hemisphere, R = right hemisphere) and anatomical labels. The ID numbers match the node numbering used in the adjacency matrix (see Fig. ??C), which also applies to all other region-by-region figures in the main text and in the supplementary text.

Region ID	Hemisphere–Label	Region ID	Hemisphere–Label
1	R–Lateral Orbitofrontal	42	L–Lateral Orbitofrontal
2	R–Pars Orbitalis	43	L–Pars Orbitalis
3	R–Frontal Pole	44	L–Frontal Pole
4	R–Medial Orbitofrontal	45	L–Medial Orbitofrontal
5	R–Pars Triangularis	46	L–Pars Triangularis
6	R–Pars Opercularis	47	L–Pars Opercularis
7	R–Rostral Middle Frontal	48	L–Rostral Middle Frontal
8	R–Superior Frontal	49	L–Superior Frontal
9	R–Caudal Middle Frontal	50	L–Caudal Middle Frontal
10	R–Precentral	51	L–Precentral
11	R–Paracentral	52	L–Paracentral
12	R–Rostral Anterior Cingulate	53	L–Rostral Anterior Cingulate
13	R–Caudal Anterior Cingulate	54	L–Caudal Anterior Cingulate
14	R–Posterior Cingulate	55	L–Posterior Cingulate
15	R–Isthmus	56	L–Isthmus
16	R–Postcentral	57	L–Postcentral
17	R–Supramarginal	58	L–Supramarginal
18	R–Superior Parietal	59	L–Superior Parietal
19	R–Inferior Parietal	60	L–Inferior Parietal
20	R–Precuneus	61	L–Precuneus
21	R–Cuneus	62	L–Cuneus
22	R–Pericalcarine	63	L–Pericalcarine
23	R–Lateral Occipital	64	L–Lateral Occipital
24	R–Lingual	65	L–Lingual
25	R–Fusiform	66	L–Fusiform
26	R–Parahippocampal	67	L–Parahippocampal
27	R–Entorhinal	68	L–Entorhinal
28	R–Temporal Pole	69	L–Temporal Pole
29	R–Inferior Temporal	70	L–Inferior Temporal
30	R–Middle Temporal	71	L–Middle Temporal
31	R–Banks of Superior Temporal Sulcus	72	L–Banks of Superior Temporal Sulcus
32	R–Superior Temporal	73	L–Superior Temporal
33	R–Transverse Temporal	74	L–Transverse Temporal
34	R–Insula	75	L–Insula
35	R–Thalamus Proper	76	L–Thalamus Proper
36	R–Caudate	77	L–Caudate
37	R–Putamen	78	L–Putamen
38	R–Pallidum	79	L–Pallidum
39	R–Accumbens	80	L–Accumbens
40	R–Hippocampus	81	L–Hippocampus
41	R–Amygdala	82	L–Amygdala

SXV Citation diversity statement

Recent work in neuroscience and other fields has identified a bias in citation practices such that papers from women and other minorities are under-cited relative to the number of such papers in the field [9–14]. Here we sought to proactively consider choosing references that reflect the diversity of the field in thought, form of contribution, gender, race, geographic location, and other factors. We used automatic classification of gender based on the first names of the first and last authors [9], with possible combinations including male/male, male/female, female/male, female/female. Excluding self-citations to the senior authors of our current paper, the main text references contain 61% male/male, 8% male/female, 24% female/male, 7% female/female. We look forward to future work that could help us to better understand how to support equitable practices in science.

References

[1] J. Andrew Henrie and Robert Shapley. Lfp power spectra in v1 cortex: The graded effect of stimulus contrast. *Journal of Neurophysiology*, 94(1):479–490, 2005.

[2] Eric Lowet, Mark Roberts, Avgis Hadjipapas, Alina Peter, Jan van der Eerden, and Peter De Weerd. Input-dependent frequency modulation of cortical gamma oscillations shapes spatial synchronization and enables phase coding. *PLOS Computational Biology*, 11(2):1–44, 02 2015.

[3] Mark J. Roberts, Eric Lowet, Nicolas M. Brunet, Marije Ter Wal, Paul Tiesinga, Pascal Fries, and Peter De Weerd. Robust gamma coherence between macaque v1 and v2 by dynamic frequency matching. *Neuron*, 78(3):523–536, 2013.

[4] Xiaoxuan Jia, Dajun Xing, and Adam Kohn. No consistent relationship between gamma power and peak frequency in macaque primary visual cortex. *Journal of Neuroscience*, 33(1):17–25, 2013.

[5] Mikail Rubinov and Olaf Sporns. Complex network measures of brain connectivity: Uses and interpretations. *NeuroImage*, 52(3):1059–1069, 2010.

[6] Gemma Lancaster, Dmytro Iatsenko, Aleksandra Pidde, Valentina Ticcinelli, and Aneta Stefanovska. Surrogate data for hypothesis testing of physical systems. *Physics Reports*, 748:1–60, 2018.

[7] James Theiler, Stephen Eubank, André Longtin, Bryan Galdrikian, and J. Doyne Farmer. Testing for nonlinearity in time series: the method of surrogate data. *Physica D: Nonlinear Phenomena*, 58(1):77–94, 1992.

[8] Arkady Pikovsky, Michael Rosenblum, and Jürgen Kurths. *Synchronization: a universal concept in nonlinear sciences*. Cambridge university press, 2003.

[9] Jordan D. Dworkin, Kristin A. Linn, Erin G. Teich, Perry Zurn, Russell T. Shinohara, and Danielle S. Bassett. The extent and drivers of gender imbalance in neuroscience reference lists. *bioRxiv*, 2020.

[10] Daniel Maliniak, Ryan Powers, and Barbara F. Walter. The gender citation gap in international relations. *International Organization*, 67(4):889–922, 2013.

[11] Neven Caplar, Sandro Tacchella, and Simon Birrer. Quantitative evaluation of gender bias in astronomical publications from citation counts. *Nature Astronomy*, 1(6):0141, 2017.

[12] Paula Chakravartty, Rachel Kuo, Victoria Grubbs, and Charlton McIlwain. #communicationsowhite. *Journal of Communication*, 68(2):254–266, 1/18/2020 2018.

[13] Michelle L. Dion, Jane Lawrence Sumner, and Sara McLaughlin Mitchell. Gendered citation patterns across political science and social science methodology fields. 26(3):312–327, 2018.

[14] Y. Thiem, K. F. Sealey, A. E. Ferrer, A. M. Trott, and R. Kennison. Just ideas? the status and future of publication ethics in philosophy: A white paper. Technical report, 2018.
